# Deubiquitination of CDC6 by OTUD6A promotes tumour progression and chemoresistance

**DOI:** 10.1186/s12943-024-01996-y

**Published:** 2024-04-29

**Authors:** Jianfeng Cui, Xiaochen Liu, Qinghong Shang, Shuna Sun, Shouzhen Chen, Jianping Dong, Yaofeng Zhu, Lei Liu, Yangyang Xia, Yong Wang, Lu Xiang, Bowen Fan, Jiafeng Zhan, Yadi Zhou, Pengxiang Chen, Renchang Zhao, Xiaofei Liu, Nianzeng Xing, Dalei Wu, Benkang Shi, Yongxin Zou

**Affiliations:** 1 Department of Urology, Qilu Hospital, Department of Molecular Medicine and Genetics, School of Basic Medical Sciences, Shandong University, Jinan, Shandong 250012 China; 2 The Key Laboratory of Experimental Teratology, Ministry of Education and Department of Molecular Medicine and Genetics, School of Basic Medical Sciences, Qilu Hospital, Shandong University, Jinan, Shandong 250012 China; 3 Department of Clinical laboratory, Qilu Hospital, Shandong University, Jinan, Shandong 250012 China; 4 Helmholtz International Lab, State Key Laboratory of Microbial Technology, Shandong University, Qingdao, Shandong 266237 China; 5 Department of Dermatology, The Affiliated Hospital of Shandong University of Traditional Chinese Medicine, Shandong Provincial Hospital of Traditional Chinese Medicine, Jinan, Shandong 250011 China; 6 Department of Urology, Shouguang People’s Hospital, Weifang, Shandong 262750 China; 7 Department of Radiation Oncology, Qilu Hospital, Shandong University, Jinan, Shandong 250012 China; 8 Department of Thoracic Surgery, Qilu Hospital, Shandong University, Jinan, Shandong 250012 China; 9 Departement of Breast and Thyroid Surgery, The Affiliated Hospital of Shandong University of Traditional Chinese Medicine, Shandong Provincial Hospital of Traditional Chinese Medicine, Jinan, Shandong 250011 China; 10 Department of Urology, National Cancer Center/National Clinical Research Center for Cancer/Cancer Hospital, Chinese Academy of Medical Sciences and Peking Union Medical College, Beijing, 100021 China

**Keywords:** CDC6, OTUD6A, Deubiquitinating enzyme, Tumorigenesis

## Abstract

**Background:**

CDC6 is an oncogenic protein whose expression level fluctuates during the cell cycle. Although several E3 ubiquitin ligases responsible for the ubiquitin-mediated proteolysis of CDC6 have been identified, the deubiquitination pathway for CDC6 has not been investigated.

**Methods:**

The proteome-wide deubiquitinase (DUB) screening was used to identify the potential regulator of CDC6. Immunofluorescence, protein half-life and deubiquitination assays were performed to determine the protein stability of CDC6. Gain- and loss-of-function experiments were implemented to analyse the impacts of OUTD6A-CDC6 axis on tumour growth and chemosensitivity in vitro. N-butyl-N-(4-hydroxybutyl) nitrosamine (BBN)-induced conditional *Otud6a* knockout (CKO) mouse model and tumour xenograft model were performed to analyse the role of OTUD6A-CDC6 axis in vivo. Tissue specimens were used to determine the association between OTUD6A and CDC6.

**Results:**

OTUD6A interacts with, depolyubiquitinates and stabilizes CDC6 by removing K6-, K33-, and K48-linked polyubiquitination. Moreover, OTUD6A promotes cell proliferation and decreases sensitivity to chemotherapy by upregulating CDC6. CKO mice are less prone to BCa tumorigenesis induced by BBN, and knockdown of OTUD6A inhibits tumour progression in vivo. Furthermore, OTUD6A protein level has a positive correlation with CDC6 protein level, and high protein levels of OTUD6A and CDC6 are associated with poor prognosis in patients with bladder cancer.

**Conclusions:**

We reveal an important yet missing piece of novel DUB governing CDC6 stability. In addition, our findings propose a model for the OTUD6A-CDC6 axis that provides novel insights into cell cycle and chemosensitivity regulation, which may become a potential biomarker and promising drug target for cancer treatment.

**Supplementary Information:**

The online version contains supplementary material available at 10.1186/s12943-024-01996-y.

## Background

Genomic DNA needs to be replicated no more than once per cell cycle [1]. In addition, the genome is vulnerable to endogenous and exogenous damaging insults, and the persistent replicative stress caused by stalled and collapsed replication forks leads to induction of the DNA damage response (DDR) [2]. Therefore, complex mechanisms are needed to monitor and regulate DNA replication to preserve cellular genomic stability [3]. Dysregulation of DNA replication and the DDR are associated with various diseases, including tumorigenesis, and potentially underlie the process of aging [4]. In eukaryotes, replication origins of DNA are directly recognized and bound by the origin recognition complex (ORC). Subsequently, cell division cycle 6 (CDC6) and chromatin licensing and DNA replication factor 1 (CDT1) are recruited, and the minichromosome maintenance protein (MCM) 2–7 complex is subsequently loaded onto the replication origin to form the pre-replicative complex (pre-RC) [5]. As CDC6 is one of the core proteins of the pre-RC, its mutation or absence prevents pre-RC assembly and origin licensing [6]. In addition to its function in pre-RC formation, CDC6 localizes to the centrosome and is required for proper centrosome assembly and duplication [7]. Moreover, CDC6 is required for ataxia telangiectasia and Rad3-related protein (ATR)-dependent activation of the DDR induced by replication stress [8]. Inhibition of CDC6 expression together with a Chk1/2 inhibitor, could reduce TopBP1 protein levels and ATR S428 and Cdc25C S216 phosphorylation, which results in inhibiting ATR-Chk1 signalling and synergistically increasing treatment efficacy in prostate cancer [9]. Due to its integral role in cell cycle progression, aberrations in CDC6 lead to various physiological and pathological changes. Recessive mutation of CDC6 is associated with Meier-Gorlin syndrome (MGS), a rare congenital anomaly syndrome characterized by impaired pre- and postnatal growth, short stature, microcephaly, microtia and absent or small patellae [10]. Aberrant upregulation of CDC6 has been found in a broad range of human cancers, including lung cancer, colon cancer, and breast cancer, and correlates with poor prognosis [9, 11, 12].

The expression of CDC6 needs to be tightly controlled during the cell cycle. CDC6 is transcriptionally regulated by early region 2 binding factor (E2F) transcription factors, the androgen receptor (AR) and forkhead box M1 (FOXM1) [13–15]. However, it is noteworthy that the protein levels of CDC6 are not consistent with the trends in its mRNA levels throughout the cell cycle. The CDC6 mRNA level is relatively high in G1/S phase, when its protein level is low, suggesting that posttranslational regulation may participate in controlling the CDC6 protein level [16]. The ubiquitin‒proteasome system (UPS) is one of the major pathways regulating gene expression at the posttranscriptional level. Dysregulation of ubiquitination plays an important role in various pathological processes, including cancer and the DDR [17, 18]. CDC6 is regulated by several E3 ubiquitin ligase complexes under different conditions [16, 19–21]. CDC6 is targeted for ubiquitin-mediated degradation in early G1 phase by APC/C-CDH1 [16], SCF-CDC4 targets Cdc6 for proteolysis in late G1 and early S phase [19], CRL4-Cdt2 ubiquitinates CDC6 at the G1-S transition [20], and SCF-Cyclin F modulates the CDC6 level in the mitosis phase [21]. Protein ubiquitination is a reversible reaction and can be reversed by catalytically active deubiquitinases (DUBs). In the human proteome, there are almost 100 DUBs, consisting of six families [22]. However, whether CDC6 is also directly regulated by DUBs is unknown.

We herein performed a proteome-wide DUB screening to identify the regulator of CDC6 and identified OTU domain-containing 6 A (OTUD6A) as the first potent DUB for CDC6 depolyubiquitination. We found that OTUD6A can directly interact with CDC6 and reverse its ubiquitination. We further characterized the pivotal role of OTUD6A in tumour progression and chemoresistance via upregulation of CDC6. Together, these results reveal an important yet missing piece comprising a novel DUB that controls CDC6 stability and demonstrate the regulatory function of OTUD6A under both physiological and pathological conditions.

## Methods

### Cell culture and reagents

HEK293T, HEK293, UMUC3 and U2OS cells were obtained from the American Type Culture Collection (ATCC) and cultured in DMEM (Gibco, 11,995,065). HeLa, T24, 5637, 786-O, H1299, and KYSE150 cells were obtained from ATCC and cultured in RPMI-1640 medium (Gibco, 11,875,093). SV-HUC-1 cells were obtained from ATCC and cultured in F12K medium (Macgene, CM10025). RT4 cells were obtained from ATCC and cultured in McCoy’s 5 A medium (Sigma-Aldrich, M4892). The medium was supplemented with 10% fetal bovine serum (Gibco, 10,099,141 C). The cells were maintained at 37 ℃ in an incubator with 5% CO_2_.

MG132 (HY-13,259), cycloheximide (HY-12,320), hydroxyurea (HU, HY-B0313), gemcitabine (HY-17,026), methotrexate (HY-14,519), chloroquine (HY-17,589 A), CVT-313 (HY-15,339), VE-821 (HY-14,731) and GDC-0575 (HY-112,167) were purchased from MedChemExpress. Thymidine (T1895) and nocodazole (M1404) were purchased from Sigma–Aldrich. N-butyl-N-(4-hydroxybutyl) nitrosamine (BBN, B0938) was purchased from TCI.

### Plasmids

The human DUB plasmid library and the HA-Ub WT, K11R, K27R, K29R, K33R, K48R and K63R plasmids were provided by Pro. CJ Gao [23]. The pCGN.CSH.FL42 plasmid encoding HA-tagged CDC6 was a gift from Pro. L. Drury (Clare Hall Laboratories, Cancer Research UK, London, England). The plasmids encoding Flag-YFP-N terminus (Flag-YN), HA-YFP-C terminus (HA-YC), Flag-YFP-N terminus-OTUD6A (YN-OTUD6A) or HA-YFP-C terminus-CDC6 (YC-CDC6) were purchase from GeneChem Inc. (Shanghai, China). The plasmids encoding Flag-tagged OTUD6A-N-terminal (1-145 aa) and OTUD6A-C-terminal (129–288 aa) and the plasmids encoding GST-tagged full-length OTUD6A, OTUD6A-N-terminal (1-145 aa) and OTUD6A-C-terminal (129–288 aa) were gifts from Pro. LY Huang [24]. The plasmids encoding the HA-tagged CDC6 AAA (S54A, S74A and S106A) and CDC6 DDD (S54D, S74D and S106D) mutants were gifts from Pro. JF Diffley [25]. The Myc-CDC6, Myc-CDH1 and Myc-Cyclin F plasmids were purchased from GeneCopoeia (Guangzhou, China). The OTUD6A catalytic site mutation (C152A) was generated by site-directed mutagenesis (QuickMutation™ Site-Directed Mutagenesis Kit, Beyotime) according to the manufacturer’s protocol. The primers used for the construction of mutant vectors are shown in Supplementary Table 1.

### Total RNA extraction, reverse transcription PCR and qPCR

Extraction of total RNA, reverse transcription PCR, and qRT-PCR (qPCR) were performed as described previously [26]. Briefly, total RNA was isolated using TRIzol reagent (Invitrogen, 15,596,026) according to the manufacturer’s protocol. One microgram of RNA was reverse transcribed into cDNA using the PrimeScript RT Reagent Kit (Accurate Biotechnology, AG11706). qPCR was performed using the LightCycler 480 system (Roche, Mannheim, Germany). The qPCR primers used to detect the indicated gene products were purchased from Sangon Biotech and are described in Supplementary Table 2.

### Western blot analysis

Western blotting was performed as described previously [26]. In brief, proteins in samples were separated on SDS polyacrylamide gels by electrophoresis and transferred to PVDF membranes (Millipore, IPVH00010). Then, the membranes were blocked with 5% skim milk for 1 h and incubated with the indicated primary antibodies at 4 ℃ overnight. The membranes were incubated with HRP-conjugated secondary antibodies and visualized in an Amersham™ ImageQuant™ 800 instrument (GE Healthcare, Fairfield, USA) with an ECL kit (Beyotime, P0018FM). The primary antibodies are listed in Supplementary Table 3. Band intensities were quantified with ImageJ software (version 1.6.0.32, National Institutes of Health, USA). The original WB figures were shown in Supplementary Figs. 14–19.

### Immunofluorescence

Cellular immunofluorescence staining was performed as described previously [27]. Briefly, cells were grown on cover slips and harvested at the indicated times. The cover slips were washed with PBS three times and fixed with 4% paraformaldehyde for 20 min. The cells were permeabilized with 0.2% Triton X-100 in PBS for 20 min and blocked with 5% goat serum in PBS for 1 h at room temperature. The cover slips were incubated with the appropriate primary antibodies overnight. The cover slips were incubated with secondary antibodies conjugated to Alexa Fluor 488 (Abcam, ab150113) or 594 (Abcam, ab150080). The cells were further stained with DAPI (Sigma-Aldrich, F6057) and imaged with a fluorescence microscope (BX51, Olympus Life Science, Tokyo, Japan). To avoid bleed-through effects in double-staining experiments, each dye was analysed independently in multitracking mode, and images were merged with ImageJ. To analyse the colocalization status of OTUD6A and CDC6, we used the Coloc2 plugin function of calculating the M1 and M2 Manders’ coefficients and Pearson’s coefficients by ImageJ. M1 represents the proportion of overlapping pixel intensity of CDC6 and OTUD6A in CDC6 pixel intensity. M2 represents the proportion of overlapping pixel intensity of CDC6 and OTUD6A in OTUD6A pixel intensity.

For immunofluorescence staining of embryos, paraffin sections were baked for 1 h at 65 ℃, dewaxed in dimethylbenzene, and hydrated in a decreasing ethanol series. Sections were immersed in Tris-EDTA antigen retrieval buffer (Proteintech, PR30002), boiled in a microwave at 95–100 ℃ for 17 min, and cooled naturally to room temperature. Tissues were blocked in 5% bovine serum albumin (BSA) for 1 h before incubation with the primary antibody overnight at 4 ℃. Then, the tissues were incubated with the indicated secondary antibody at room temperature for 1 h and stained with DAPI. The tissues were imaged with a fluorescence microscope (VS120, Olympus Life Science, Tokyo, Japan).

### Immunoprecipitation

Immunoprecipitation (IP) was performed by using a Catch and Release® v2.0 Reversible Immunoprecipitation System Kit (Millipore, 17–500) following the manufacturer’s protocol. In brief, cell lysates were prepared by incubating cells in Western and IP buffer (Beyotime, P0013) with protease inhibitors (NCM Biotech, P001) on ice for 20 min, followed by sonication (5 s, 15 cycles). This was followed by centrifugation at 15,000 rpm for 15 min at 4 ℃. Two thousand micrograms of protein was added to the affinity column along with 1 µg of the indicated antibody and 10 µL of antibody capture affinity ligand (total 500 µL) and incubated on a rotary shaker at 4 ℃ overnight. The column was centrifuged and washed 3 times, and proteins were then eluted with 70 µL of elution buffer. The eluates were boiled with SDS-PAGE loading buffer at 99 ℃ for 7 min and analysed by Western blotting.

### Haematoxylin and eosin (H&E) staining and immunohistochemistry

H&E staining and immunohistochemistry were performed as described previously [28]. In brief, paraffin sections were dewaxed and hydrated, and the tissues were stained with haematoxylin solution for 1 min and rinsed in diluted water for 3 min. Then, the tissues were stained with eosin solution for 2 min and rinsed in diluted water for 3 min prior to dehydration with an increasing ethanol series and clearing in dimethylbenzene. The sections were sealed with Neutral Balsam. IHC staining was performed using a PV-9001 kit (ZSGB-BIO) following the manufacturer’s protocol. The sections were imaged with a microscope (BX51, Olympus Life Science, Tokyo, Japan). The staining intensity was defined as follows: 0 (negative), 1 (weak), 2 (moderate), and 3 (strong). The IHC-profiler with ImageJ was used to digitally scoring the positive staining percentage of tumour cells for each intensity according to the previous study [29]. The protein expression was quantified using the H-score, The H-score is calculated as follows: (1 × percentage of weak staining) + (2 × percentage of moderate staining) + (3 × percentage of strong staining), ranging from 0 to 300. In order to evaluate the accuracy of the computer-assisted measurement, the computerized images and the computer-assisted measurements were verified with two pathologist-based scoring results. The receiver operating curve (ROC) analysis was used to determine the probable cutoff value level, and the BCa patients were divided into high and low expression groups according to the optimal cutoff value of OTUD6A (H-score: 177) and CDC6 (H-score: 132).

### Mouse models

The *Otud6a*^*flox/flox*^ mouse (C57BL/6 N) model was established by CRISPR/Cas-mediated genome engineering by Cyagen Biosciences (Suzhou, China). *Dppa3*^*em1(IRES−Cre)*^ mice (C57BL/6J) were purchased from Shanghai Model Organism Center, Inc. (Shanghai, China). To generate *Otud6a* knockout mice, exons 1 of the *Otud6a* were flanked with CRISPR/Cas9 mediated insertion of LoxP sites (Supplementary Fig. 1o), which the frameshift caused by deletion of exon 1 eliminated the gene product prematurely, and the deletion region contained no other known gene. Then *Otud6a*^*flox/flox*^ mice were crossed with *Dppa3-Cre* (Dppa3-Cre can exert efficient Cre recombination enzyme activity during the early stage of embryonic development and in germ cell line [30]) mice to obtain *Dppa3‐Cre; Otud6a*^*null/Y*^ and *Dppa3‐Cre; Otud6a*^*null/null*^ mice, designated as *Otud6a*‐CKO mice in the study. *Otud6a*^*flox/flox*^ mice and CKO mice were identified by PCR analysis of tail tip genomic DNA. Amplification of DNA from the *Otud6a*-targeted and wild-type mice resulted in PCR products of 203 bp and 123 bp, respectively, and amplification of DNA from CKO mice produced a PCR product of 187 bp. The PCR primers for genotyping are listed in Supplementary Table 4.

The tumour xenograft model was established as previously described [26]. In brief, four-week-old female BALB/c (nu/nu) mice were purchased from Vital River Laboratory Animal Technology Co. Ltd (Beijing, China). The indicated cells were subcutaneously implanted into the dorsal flank of each mouse. Tumour volumes were calculated every 4 days after a 7-day adaptation period, and mice were sacrificed 31 days after implantation. For gemcitabine treatment assays, fourteen days after tumour inoculation, the mice were randomly divided into two groups. The mice were injected intraperitoneally with gemcitabine (50 mg/kg in DMSO) or DMSO once every 7 days, and the mice were sacrificed 4 weeks post implantation. Tumours were measured with a Vernier calliper, and tumour volumes were calculated with the following formula: V = (a × b^2^) / 2, where a and b represent the longest and shortest diameters, respectively. The tumours were harvested, weighed, and embedded in paraffin for IHC staining, and protein and RNA were extracted for Western blotting and qPCR, respectively.

For the BBN‑induced mouse model of BCa, eight-week-old indicated male mice were provided with unrestricted access to drinking sterile tap water containing 0.05% BBN for 12 weeks and then were replaced with sterile tap water until the end of the experiment. The control group of mice was given sterile tap water. To analyse the progress of bladder tumorigenesis induced by BBN and the protein levels of OTUD6A and CDC6 during the progress, randomly selected mice were sacrificed at the indicated time. For analysing the role of OTUD6A in regulating bladder tumorigenesis, all mice were sacrificed at week 20 after BBN treatment. The urinary bladder was removed and embedded in paraffin for H&E and IHC staining. All experiments were approved by the Shandong University Animal Care Committee, and all procedures were performed in compliance with the institutional guidelines.

### Statistical analysis

All data were statistically analysed using GraphPad Prism (version 8.0.2, GraphPad Software, CA, USA). The data are presented as the mean ± standard deviation (SD) values as indicated in the figure legends. Two-tailed unpaired Student’s *t* test was used to compare two groups of data. Two-tailed paired Student’s *t* test was used to compare data for matched BCa tissues. The chi-square test was used for comparison of categorical data, and Spearman correlation analysis was used for comparison of continuous variables. Survival curves were generated using Kaplan-Meier estimates, and differences between the survival curves were compared using the log-rank test. *P* values < 0.05 were considered to be statistically significant.

See supplementary materials for additional methods.

## Results

### Identification of OTUD6A as a positive regulator of CDC6

**Fig. 1:**
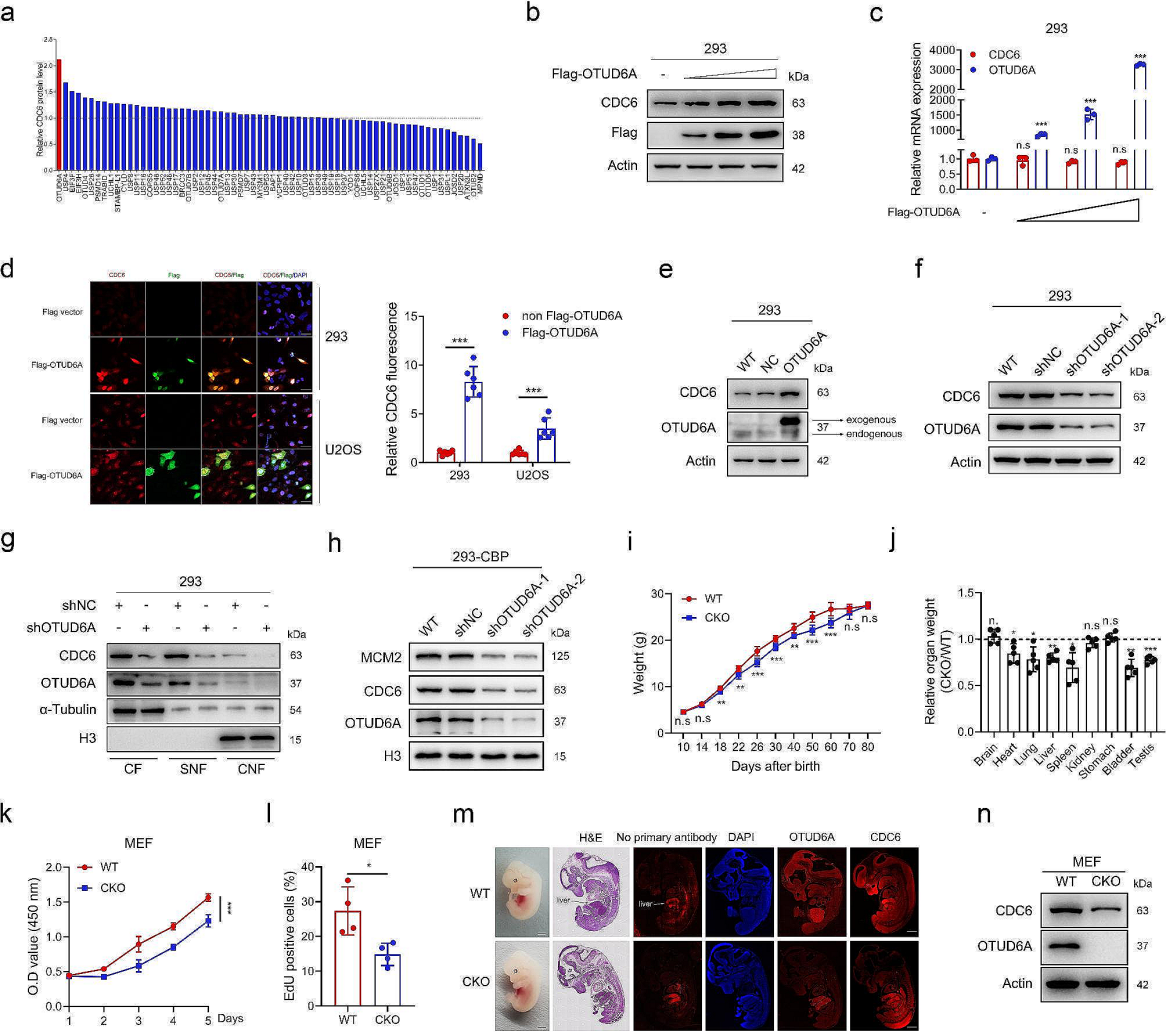
OTUD6A upregulates CDC6 protein level. **a**, Quantitative analysis of CDC6 protein levels shown in Supplementary Fig. 1a. **b**, **c**, Increasing amounts of Flag-OTUD6A plasmids were transfected into HEK293 (293) cells, and the protein levels of endogenous CDC6 and exogenous OTUD6A were determined by Western blotting (**b**). The mRNA levels of CDC6 and OTUD6A were determined by qPCR (**c**), and the levels in empty Flag vector cells were set as 1. **d**, 293 and U2OS cells were transfected with the empty Flag vector or Flag-OTUD6A plasmid. An additional 24 h later, the cells were fixed and incubated with the Flag and CDC6 antibodies. Representative immunofluorescence images are shown (left). Scale bars, 20 μm. Quantification of the relative fluorescence intensity of CDC6 is shown (right), and the fluorescence intensity of CDC6 in Flag-OTUD6A untransfected or empty Flag vector-transfected cells was set as 1.**e, f**, CDC6 and OTUD6A expression levels were measured in the indicated 293 cells by Western blotting. **g**, Cytoplasmic, soluble nuclear and chromatin-bound nuclear fractions were extracted from the indicated cells using subcellular fractionation assay and detected by Western blotting. CF, cytoplasmic fractions; SNF, soluble nuclear fractions; CNF, chromatin-bound nuclear fractions. **h**, Chromatin-bound proteins (CBP) were extracted from the indicated 293 cells and analysed by Western blotting. **i**, Weight curves of WT and OTUD6A knockout (CKO) mice are shown (WT, *n* = 7; CKO, *n* = 7). **j**, Relative quantification of tissue weights from 8-week-old WT (*n* = 5) and CKO (*n* = 5) mice. **k, l**, The proliferation of the indicated mouse embryonic fibroblasts (MEFs) was measured by CCK8 assays (**k**) and EdU incorporation assays (**l**). **m**, Representative bright-field, H&E and immunofluorescence images of WT and CKO embryos at embryonic day (E) 13.5. The liver tissue is autofluorescent. Scale bar, 1 mm. **n**, CDC6 and OTUD6A expression levels were measured in WT and CKO mouse-derived MEFs by Western blotting. All quantitative analyses were based on three independent experiments. The error bars indicate the SDs. *P < 0.05, **P < 0.01, ***P < 0.001, n.s. not significant, based on two-tailed Student’s *t* test

To systematically identify the potential DUBs responsible for CDC6 regulation, we performed a proteome-wide DUB screening by transiently transfecting 66 DUB-encoding plasmids into HEK293 (293) cells individually and measuring endogenous CDC6 protein levels. Among the DUBs tested, OTUD6A emerged as the DUB with the strongest upregulating effect on the CDC6 protein level (Fig. 1a and Supplementary Fig. 1a). Ectopic expression of OTUD6A resulted in an elevation of the endogenous CDC6 protein but not mRNA level in a dose-dependent manner (Fig. 1b, c). Immunofluorescence (IF) staining further confirmed that overexpression of the OTUD6A upregulated the endogenous CDC6 protein level, compared to those transfected with empty Flag vector and Flag-OTUD6A untransfected cells (Fig. 1d), and transient transfection with empty Flag vector or Flag-OTUD6A plasmid didn’t change the cell cycle distribution (Supplementary Fig. 1b). Consistent with the results of transient transfection, stable overexpression of OTUD6A in 293 and HeLa cells also increased the protein level but not the mRNA level of CDC6 (Fig. 1e and Supplementary Fig. 1c-e). In contrast, stable knockdown of OTUD6A in 293, HeLa and U2OS cells using short hairpin RNAs (shRNAs) markedly decreased the CDC6 protein level but had no effect on the CDC6 mRNA level (Fig. 1f and Supplementary Fig. 1f-j). The upregulation of CDC6 by OTUD6A was not due to changes in the cell cycle as neither stable overexpression nor knockdown of OTUD6A influenced the cell cycle distribution in asynchronous cells (Supplementary Fig. 1k, l).

The subcellular location of CDC6 protein changes in a cell cycle-dependent manner which is important for its function [7, 31–33]. Thus we fractionated cell lysates into cytoplasmic, soluble and chromatin-bound fractions, and found that overexpression of OTUD6A increased while knockdown of OTUD6A decreased the CDC6 protein level in all three fractions (Fig. 1g and Supplementary Fig. 1m, n). Moreover, knockdown of OTUD6A was accompanied by decreased loading of MCM2, a subunit of pre-RC, onto chromatin (Fig. 1h), suggesting that knockdown of OTUD6A prevents pre-RC assembly.

To confirm the role of OTUD6A in CDC6 regulation in vivo, we crossed *Otud6a*^*flox/flox*^ mice with *Dppa3-Cre* (which exert efficient Cre recombination enzyme activity during the early stage of embryonic development and in germ cell line [30]) mice to generate conditional *Otud6a* knockout (CKO) mice (Supplementary Fig. 1o, p). The abrogation of OTUD6A expression was confirmed by Western blot analyses (Supplementary Fig. 1q). *Otud6a* CKO mice were born at the expected Mendelian ratio (Supplementary Fig. 1r), and the body weight showed no significant difference between CKO and WT newborn mice (Supplementary Fig. 1s, t), suggesting OTUD6A deficiency did not result in growth abnormalities during embryogenesis or fetal development. However, the postnatal growth curves of wild-type and CKO mice started to diverge significantly 18 days after birth (Fig. 1i and Supplementary Fig. 1u). The decrease in body size is also reflected in the weight and size of most of organs examined at adult stage (Fig. 1j and Supplementary Fig. 1v). The decreased body and organ weight in CKO mice were not due to less food intake, which was comparable to control mice (Supplementary Fig. 1w). To investigate whether the growth retardation of *Otud6a* CKO mice reflected a defect in cellular proliferation, we examined the growth properties of *Otud6a* knockout mouse embryonic fibroblasts (MEFs), and the results showed that the cell proliferation of *Otud6a* knockout MEFs was considerably slower than that of wild-type MEFs (Fig. 1k, l and Supplementary Fig. 1x). Together, these results indicate that *Otud6a* knockout resulted in a general growth deficit.

We then determine the effect of OTUD6A knockout on CDC6 expression in vivo. Most of the tissues from CKO mice with successful deletion of OTUD6A exhibited lower expression of the CDC6 protein (Supplementary Fig. 1q, y). Similar results were observed in mouse embryos (Fig. 1m). Moreover, the CDC6 protein level but not mRNA level was reduced in CKO mouse MEFs (Fig. 1n and Supplementary Fig. 1z). Collectively, these results demonstrate that OTUD6A positively regulates the CDC6 protein level both in vivo and in vitro.

### OTUD6A directly interacts with CDC6

**Fig. 2:**
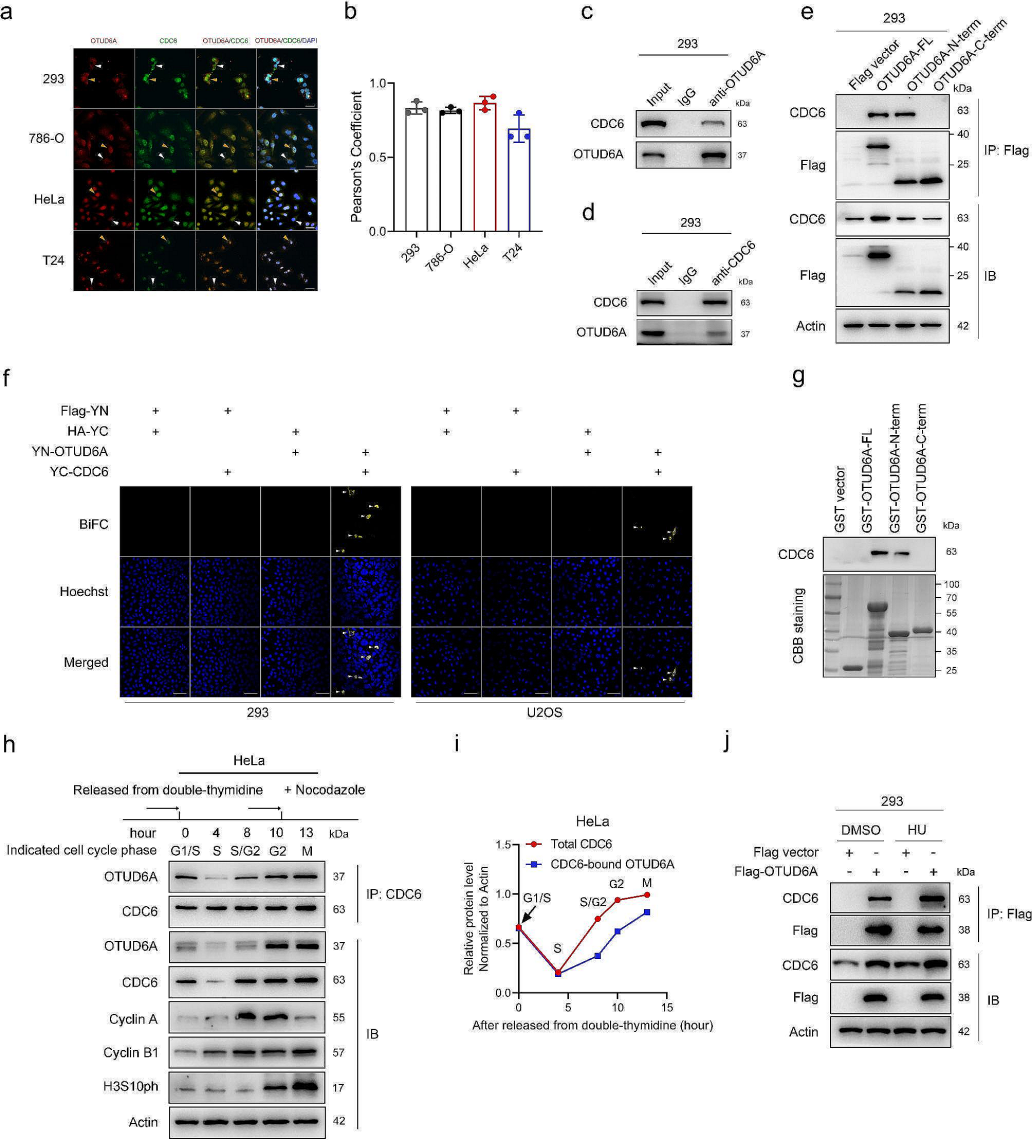
OTUD6A interacts with CDC6. **a**, Representative immunofluorescence images of endogenous OTUD6A (red) and CDC6 (green) in the indicated cells are shown. Scale bars, 20 μm. **b**, Pearson’s coefficient analysis was used to analyse the colocalization of OTUD6A and CDC6. **c**, 293 cell lysates were prepared and subjected to IP with control IgG or an anti-OTUD6A antibody. The immunoprecipitates were analysed by Western blotting. **d**, 293 cell lysates were prepared and subjected to IP with control IgG or an anti-CDC6 antibody. The immunoprecipitates were analysed by Western blotting. **e**, The indicated OTUD6A constructs were cotransfected with HA-CDC6 into 293 cells for 24 h. Whole-cell lysates were prepared and subjected to IP with an anti-Flag antibody. The immunoprecipitates were analysed by Western blotting. **f**, Representative confocal images of bimolecular fluorescent complimentary (BiFC) experiment are shown. White arrows represent that OTUD6A interacts with CDC6. Scale bars, 50 μm. **g**, A GST pull-down assay was performed to indicate the direct interaction between OTUD6A and CDC6. CBB staining, Coomassie brilliant blue staining. **h**, HeLa cells were synchronized at the G1/S boundary using a double-thymidine block. Whole-cell lysates were collected at the indicated time points after release into fresh medium and subjected to IP with an anti-CDC6 antibody. The immunoprecipitates were analysed by Western blotting. **i**, The intensities of the CDC6-bound OTUD6A (IP) and total CDC6 (IB) bands were quantified. **j**, 293 cells transfected with the indicated vectors were treated with HU (2.5 mM) or DMSO for 24 h. Whole-cell lysates were prepared and subjected to IP with an anti-Flag antibody. The immunoprecipitates were analysed by Western blotting

We next examined whether OTUD6A physically interacts with CDC6. Immunofluorescence staining revealed that OTUD6A colocalized with CDC6 in the nuclei and cytoplasm (Fig. 2a, b and Supplementary Fig. 2a-d). Coimmunoprecipitation (co-IP) assays confirmed both endogenous and exogenous CDC6 and OTUD6A proteins were coimmunoprecipitated with each other from whole-cell lysates (Fig. 2c, d and Supplementary Fig. 2e, f). Consistently, mass spectrometry analysis also demonstrated that CDC6 was the intracellular binding partner of OTUD6A (Supplementary Fig. 2g). To identify the regions of OTUD6A mediating its interaction with CDC6, a series of vectors encoding Flag-tagged OTUD6A deletion mutants were transfected into 293 cells (Supplementary Fig. 2h). Co-IP assays revealed that the N-terminal region (amino acids 1-145) of OTUD6A mediated its physical interaction with CDC6 (Fig. 2e). Bimolecular fluorescent complimentary (BiFC) assays confirmed that OTUD6A directly interacts with CDC6 (Fig. 2f). GST pulldown and assays confirmed the direct interaction between the N-terminal region of OTUD6A and CDC6 (Fig. 2g).

Given that CDC6 has important functions in cell cycle regulation and that its expression fluctuates periodically during the cell cycle, we sought to determine whether the binding capacity between CDC6 and OTUD6A varies during cell cycle progression. As shown in Fig. 2h and Supplementary Fig. 2i, CDC6 protein level was low in S phase, and it began to increase in late S phase until its maximal level was reached in G2/M phase. Importantly, the interaction between OTUD6A and CDC6 was detected in late S phase and occurred predominantly in G2/M phase, following the same trend as CDC6 protein level (Fig. 2h, i). We then treated cells with the replication-damaging agent hydroxyurea (HU), which inhibits ribonucleotide reductase and leads to replication stress by fork stalling/collapse. HU treatment, which increased CDC6 expression, effectively promoted the interaction between OTUD6A and CDC6, indicating that replication stress enhanced the binding capacity between OTUD6A and CDC6 (Fig. 2j). Phosphorylation by cyclin E-CDK2 has been reported to prevent CDC6 degradation by APC/C and thus increase the stability of CDC6 [25]. However, the interaction between CDC6 and OTUD6A was not changed in cells treated with the CDK2 inhibitor CVT-313 compared with that in control cells (Supplementary Fig. 2j).

### OTUD6A depolyubiquitinates CDC6 and maintains CDC6 stability

**Fig. 3:**
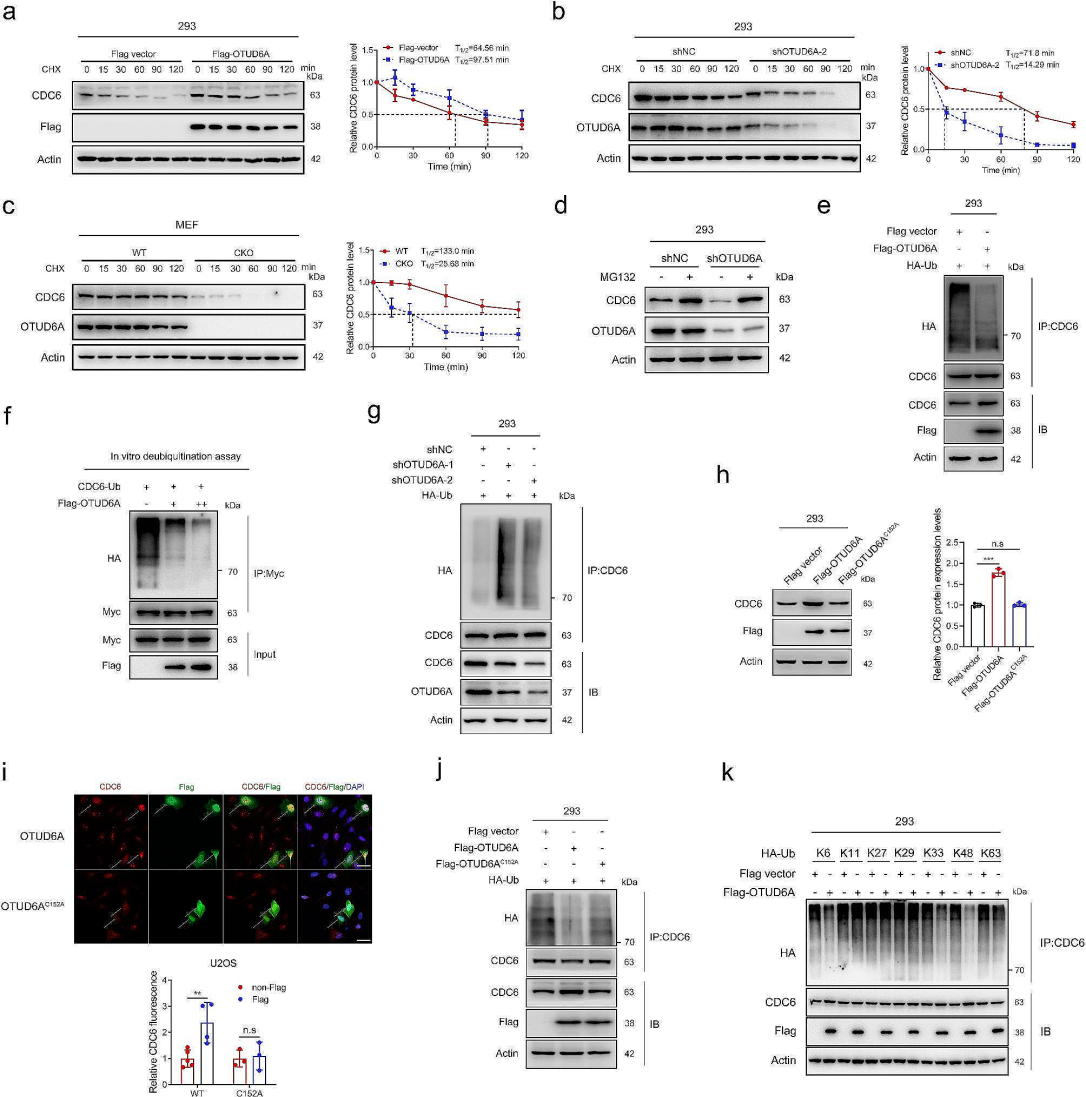
OTUD6A deubiquitinates CDC6 and promotes CDC6 stability. **a**, 293 cells transfected with the indicated vectors were treated with CHX (20 µg/mL) and harvested at the indicated time points prior to Western blotting (left). The intensities of the CDC6 bands were quantified from three independent repeated Western blotting analysis (right); the intensity at 0 min was set as 1. **b**, 293 cells with stable OTUD6A knockdown were treated with CHX and harvested at the indicated time points prior to Western blotting (left). The intensities of the CDC6 bands were quantified from three independent repeated Western blotting analysis (right), and the level at 0 min was set as 1. **c**, WT and CKO mouse-derived MEFs were treated with CHX and harvested at the indicated time points prior to Western blotting (left). The intensities of the CDC6 bands were quantified from three independent repeated Western blotting analysis (right), and the level at 0 min was set as 1. **d**, Western blot analysis of cell lysates from the indicated 293 cells treated with MG132 (20 µM) or DMSO for 6 h. **e**, 293 cells transfected with the indicated vectors were treated with MG132 (20 µM) for 6 h. Whole-cell lysates were prepared and subjected to IP with an anti-CDC6 antibody. The immunoprecipitates were analysed by Western blotting. **f**, In vitro deubiquitination assay with Flag-OTUD6A and Myc-CDC6-Ub purified from 293 cells. **g**, 293 cells with stable OTUD6A knockdown and transfected with HA-Ub were treated with MG132 for 6 h. Whole-cell lysates were prepared and subjected to IP with an anti-CDC6 antibody. The immunoprecipitates were analysed by Western blotting. **h**, Western blot analysis of proteins extracted from 293 cells transfected with the indicated vectors (left). The intensities of the CDC6 bands were quantified (right), and the level in empty Flag vector cells was set as 1. **i**, U2OS cells transfected with the indicated vectors were fixed and stained as indicated. Representative immunofluorescence images are shown (top). Scale bars, 20 μm. Quantification of the relative fluorescence intensity of CDC6 is shown (bottom), and the fluorescence intensity of CDC6 in Flag-OTUD6A untransfected cells was set as 1. **j**, 293 cells transfected with the indicated vectors were treated with MG132 for 6 h. Whole-cell lysates were prepared and subjected to IP with an anti-CDC6 antibody. The immunoprecipitates were analysed by Western blotting. **k**, Flag-OTUD6A was cotransfected with wild-type HA-Ub or its lysine residue mutants (for example, K6 indicates that all lysines except for K6 were mutated to arginine) into 293 cells for 24 h. The cells were treated with MG132 for 6 h. Whole-cell lysates were prepared and subjected to IP with an anti-CDC6 antibody. The immunoprecipitates were analysed by Western blotting. All quantitative analyses were based on three independent experiments. The error bars indicate the SDs. **P < 0.01, ***P < 0.001, n.s. not significant, based on two-tailed Student’s *t* test

The finding that OTUD6A increases the CDC6 protein level but does not affect the CDC6 mRNA level suggests that OTUD6A regulates CDC6 expression at the posttranscriptional level. Therefore, we first examined the effect of OTUD6A on CDC6 protein stability. The half-life of the endogenous CDC6 protein was prolonged in OTUD6A-overexpressing cells (Fig. 3a), whereas knockdown or knockout of OTUD6A led to a shortened half-life (Fig. 3b, c and Supplementary Fig. 3a), suggesting that OTUD6A inhibits CDC6 degradation. CDC6 protein stability is known to be associated with its phosphorylation status [25]. To explore whether CDC6 phosphorylation affects the regulation of CDC6 expression by OTUD6A, three CDK-mediated phosphorylation-related residues (S54, S74 and S106) in CDC6 were mutated to alanine (AAA) to mimic the unphosphorylated status or to aspartic acid (DDD) to mimic the phosphorylated status. Consistent with a previous report [25], the DDD mutant was expressed at higher levels but the AAA mutant was expressed at lower levels than WT CDC6 (Supplementary Fig. 3b). Overexpression of OTUD6A upregulated CDC6 protein level and prolonged the half-life of the CDC6 protein, independent of its phosphorylation status (Supplementary Fig. 3c-e). Ubiquitin-proteasome system and the autophagy-lysosome pathway are the two main mechanisms responsible for intracellular protein degradation [34], we next clarified the pathway involved in OTUD6A-mediated CDC6 regulation. The reduced CDC6 protein level caused by OTUD6A knockdown was effectively restored by treatment with the proteasome inhibitor MG132 (Fig. 3d) but not with the lysosome inhibitor chloroquine (Supplementary Fig. 3f), indicating that OTUD6A likely maintains CDC6 protein stability through the proteasomal pathway.

Deubiquitinating enzymes stabilize their substrates by removing ubiquitin chains. Indeed, overexpression of OTUD6A markedly reduced endogenous and exogenous CDC6 polyubiquitination in cells (Fig. 3e and Supplementary Fig. 3g). In vitro deubiquitination assays confirmed that OTUD6A could remove polyubiquitin chains from CDC6 (Fig. 3f). Conversely, knockdown of OTUD6A increased the polyubiquitination of CDC6 (Fig. 3g). Then, the plasmid encoding the deubiquitinase-dead mutant of OTUD6A (C152A mutant, which cysteine 152 was mutated to alanine, cysteine 152 is highly conserved site among species) was constructed. Although mutation of OTUD6A did not affect the interaction between OTUD6A and CDC6 (Supplementary Fig. 3h), the C152A mutant of OTUD6A lost the ability to upregulate CDC6 (Fig. 3h, i and Supplementary Fig. 3i-m). Consistent with this effect, this mutant also failed to prolong the half-life of CDC6 and to decrease the polyubiquitination of endogenous and exogenous CDC6 (Fig. 3j and Supplementary Fig. 3n, o), indicating that OTUD6A regulates CDC6 in a manner dependent on its DUB activity. We further investigated the role of interaction between CDC6 and OTUD6A in CDC6 protein regulation. Transient transfection with full length but not the C-terminal containing OTUD6A plasmid significantly increased the protein levels of CDC6 (Supplementary Fig. 3p). Consistently, overexpression of OTUD6A-C-terminal did not prolong the half-life of CDC6 (Supplementary Fig. 3q), indicating the direct interaction with CDC6 is required for OTUD6A to exert its function on regulating CDC6.

To extend our findings, a series of ubiquitin mutants were cotransfected with OTUD6A into cells. The results showed that OTUD6A removed K6-, K33-, and K48-linked polyubiquitin chains from CDC6 (Fig. 3k). CDC6 is targeted for proteasomal degradation by the APC/C-CDH1 and SCF-Cyclin F E3 ubiquitin ligase complex [16, 21]. We found that the downregulation and polyubiquitination of CDC6 by APC/C-CDH1 and SCF-Cyclin F could be completely reversed by OTUD6A (Supplementary Fig. 3r-u). Taken together, these data demonstrated that OTUD6A maintains CDC6 stability, removes K6-, K33- and K48-linked polyubiquitin chains, and reverses CDC6 degradation caused by APC/C-CDH1 and SCF-Cyclin F.

### OTUD6A participates in cell cycle progression by regulating CDC6

We proposed that OTUD6A, like CDC6, might function in cell cycle regulation. 4D label-free quantitative proteomic analysis was performed to explore the function of OTUD6A. A total of 6073 proteins were identified, of which 5050 were quantifiable. Overexpression of OTUD6A resulted in a total of 84 differentially expressed proteins (with a fold change of > 1.5), namely, 41 upregulated proteins and 43 downregulated proteins (Supplementary Fig. 4a and Supplementary Table 5). Notably, CDC6 was identified as one of the significantly upregulated proteins in OTUD6A-overexpressing cells (Supplementary Fig. 4a and Supplementary Table 5). By EuKaryotic Orthologous Groups (KOG) and Gene Ontology (GO) analysis of all 41 differentially upregulated proteins, we found that OTUD6A overexpression was closely associated with processes related to cell cycle progression, such as “DNA replication”, “mitotic cell cycle”, and “cell division” (Supplementary Fig. 4b-e).

**Fig. 4:**
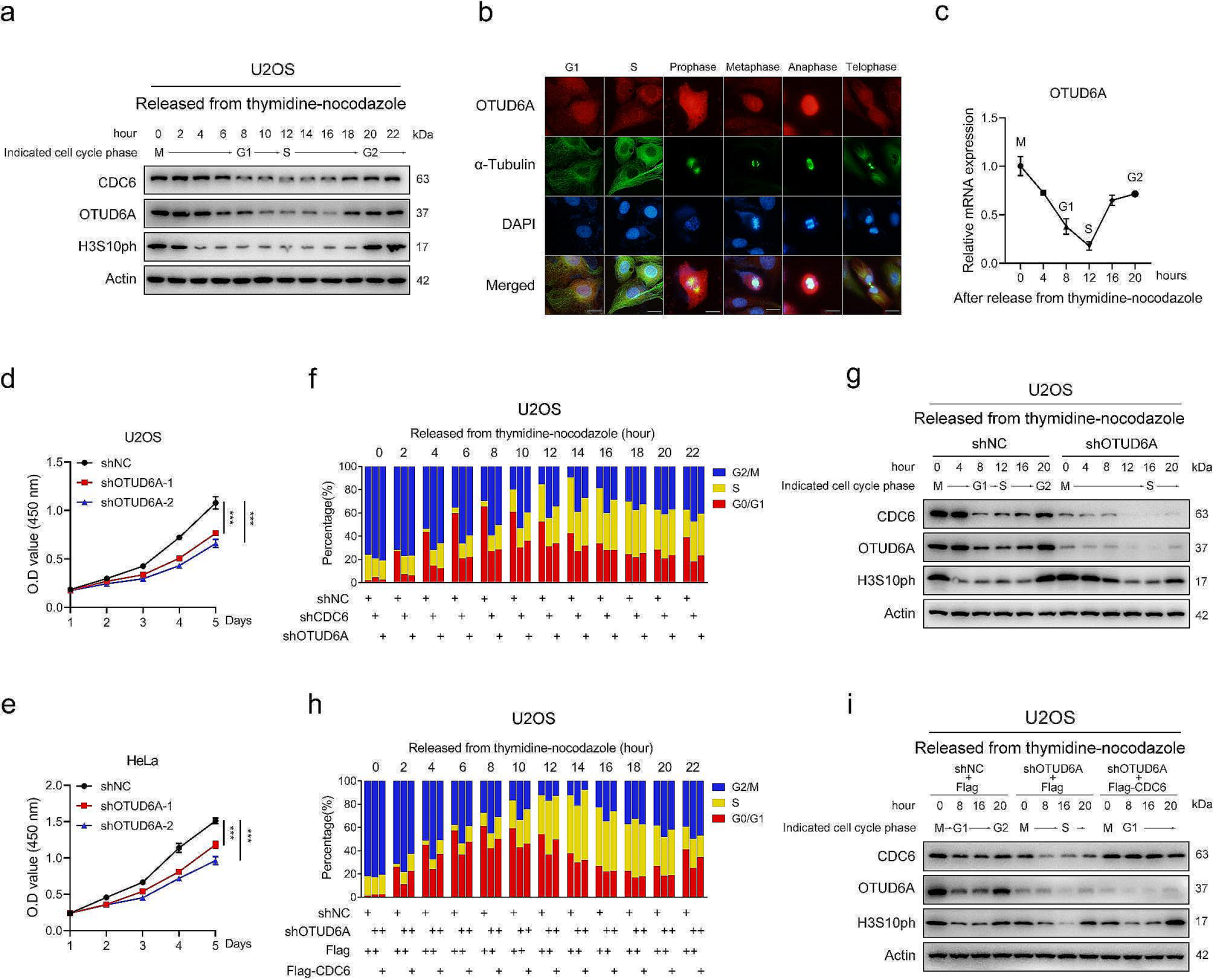
OTUD6A expression fluctuates during the cell cycle and regulates cell cycle progression. **a**, U2OS cells were synchronized in prometaphase and released into fresh medium. Cells were analysed at the indicated time points by Western blotting. **b**, Representative immunofluorescence images of U2OS cells stained with an anti-OTUD6A antibody (red), α-tubulin antibody (green), and DAPI (blue). Scale bars, 10 μm. **c**, U2OS cells were synchronized in prometaphase and released into fresh medium. mRNA level in cells was analysed at the indicated time points by qPCR. The levels at 0 h were set as 1. **d**, **e**, The proliferation of the indicated U2OS (**d**) and HeLa (**e**) cells was determined by CCK8 assays. **f-i**, U2OS cells transfected with the indicated vectors were synchronized in prometaphase and released into fresh medium. Cells were analysed at the indicated time points by flow cytometry (**f, h**) and Western blotting (**g, i**). The levels at 0 h were set as 1. All quantitative analyses were based on three independent experiments. The error bars indicate the SDs. *P < 0.05, ***P < 0.001, based on two-tailed Student’s *t* test

We next analysed OTUD6A expression during cell cycle progression. The OTUD6A protein level in U2OS cells fluctuated in a cell cycle-dependent manner, peaking strongly in the G2/M phase and then decreasing during progression to the G1 and S phases (Fig. 4a and Supplementary Fig. 4f, g). Similar results were observed in HeLa cells (Supplementary Fig. 4h-j). Notably, a fairly strong concordance of endogenous protein level of OTUD6A with that of CDC6 was observed during cell cycle progression (Fig. 4a and Supplementary Fig. 4j-l). Immunofluorescence staining confirmed that the OTUD6A abundance was elevated in mitotic cells (Fig. 4b and Supplementary Fig. 4m). Moreover, OTUD6A was localized mainly in nuclei in M- and G1-phase cells and translocated to the cytoplasm in S phase (Fig. 4b and Supplementary Fig. 4m). qPCR analysis also showed that the OTUD6A mRNA level was increased in the mitotic phase and decreased in the S phase (Fig. 4c and Supplementary Fig. 4n). Together, these data demonstrate that OTUD6A is expressed in a cell cycle-dependent manner and that its expression coincides with the CDC6 protein level during the cell cycle.

Next, we evaluated the role of OTUD6A in cell proliferation. Knockdown of OTUD6A inhibited the cell proliferation (Fig. 4d, e). Cell cycle analysis showed that inhibition of OTUD6A resulted in G2/M delay (Fig. 4f, g and Supplementary Fig. 4o, p). Similar results were obtained in CDC6-knockdown cells (Fig. 4f and Supplementary Fig. 4o, q). Importantly, overexpression of CDC6 abrogated the G2/M delay induced by knockdown of OTUD6A (Fig. 4h, i and Supplementary Fig. 4r-t), suggesting that OTUD6A regulates cell cycle progression in a CDC6-dependent manner.

### The OTUD6A-CDC6 axis promotes the tumorigenicity of cancer cells

**Fig. 5:**
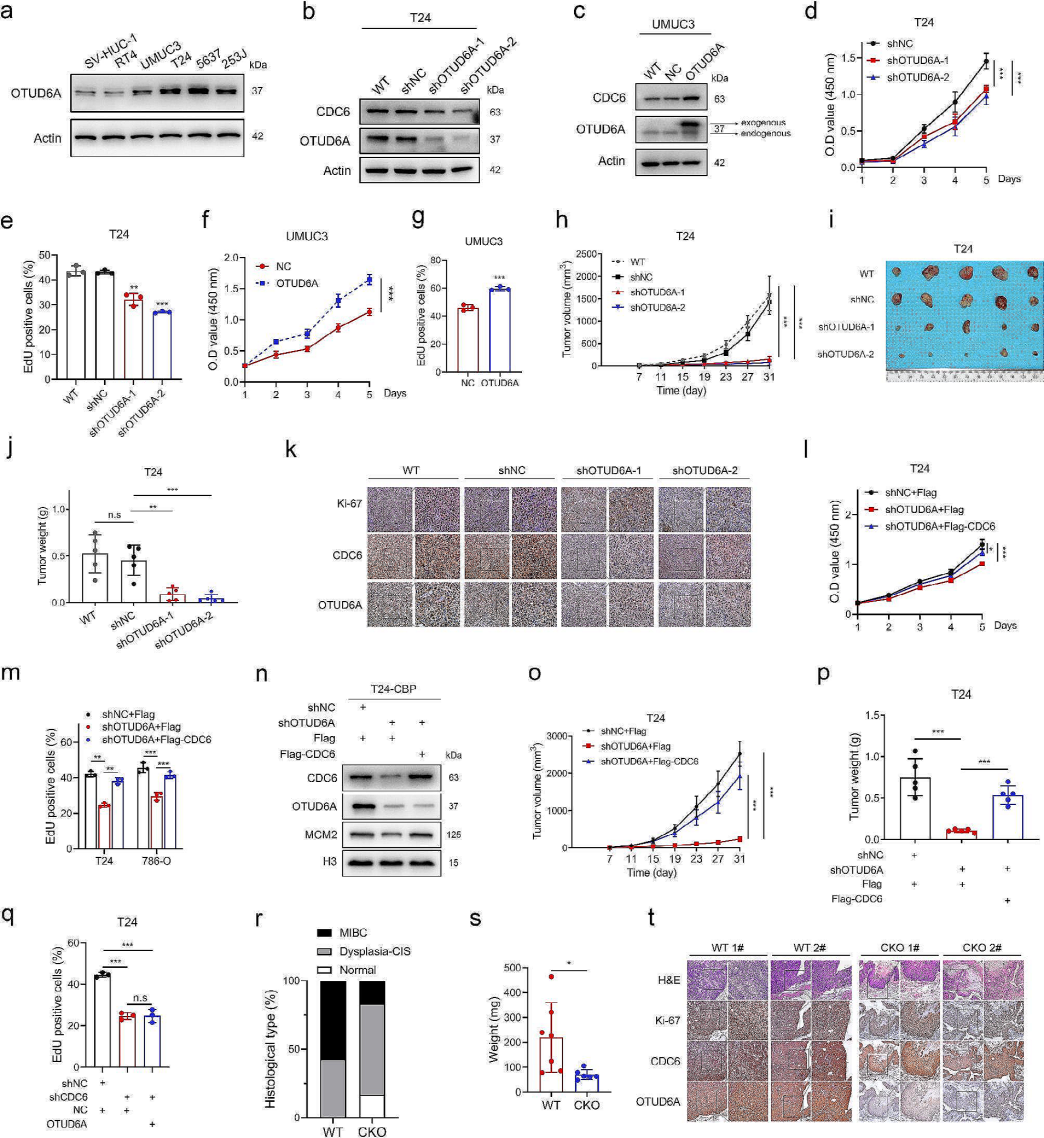
The OTUD6A-CDC6 axis promotes tumour growth. **a**, The protein expression levels of OTUD6A in BCa cells and uroepithelial SV-HUC-1 cells were determined by Western blotting. **b, c**, CDC6 and OTUD6A expression levels in the indicated T24 (**b**) and UMUC3 (**c**) cells were measured by Western blotting. **d, e**, The proliferation of OTUD6A knockdown and control T24 cells was examined by CCK8 assays (**d**) and EdU incorporation assays (**e**). **f, g**, The effect of OTUD6A overexpression on UMUC3 cell proliferation was examined by CCK8 assays (**f**) and EdU incorporation assays (**g**). **h**, Growth curves of the indicated T24 tumours are shown. Tumours were measured every 4 days. **i**, An image of subcutaneous tumours formed by the indicated T24 cells is shown. **j**, The indicated T24 tumours were weighed. **k**, Representative IHC images indicating Ki-67, CDC6 and OTUD6A expression in the indicated T24 tumours are shown. Scale bars, 50 μm (left) and 20 μm (right). **l, m**, CCK8 assays (**l**) and EdU incorporation assays (**m**) were used to examine the proliferation of the indicated T24 cells. **n**, Chromatin-bound proteins (CBP) were extracted from the indicated T24 cells and analysed by Western blotting. **o**, Growth curves of the indicated T24 tumours are shown. Tumours were measured every 4 days. **p**, The indicated T24 tumours were weighed. **q**, EdU incorporation assays were used to examine the proliferation of the indicated T24 cells. **r**, Different histologic types in the indicated mouse bladders after 20 weeks of BBN treatment are shown (WT mice, *n* = 7; CKO mice, *n* = 6). **s**, The mouse bladders were weighed. **t**, Representative H&E staining images and IHC images indicating Ki-67, CDC6 and OTUD6A expression in the indicated mouse bladders. Scale bars, 100 μm (left) and 50 μm (right). All quantitative analyses were based on three independent experiments. The error bars indicate the SDs. *P < 0.05, **P < 0.01, ***P < 0.001, n.s. not significant, based on two-tailed Student’s *t* test

Owing to its role in cell cycle progression and CDC6 regulation, we speculated that OTUD6A may function as an oncogene. To test this possibility, we first examined endogenous OTUD6A expression in the immortalized human uroepithelial cell line SV-HUC-1 and five bladder cancer (BCa) cell lines. The protein levels of OTUD6A in UMUC3, T24, 5637 and 253 J cells were much higher than those in SV-HUC-1 cells (Fig. 5a). We then established stable OTUD6A knockdown cancer cell lines including T24, 5637, 786-O (clear cell renal carcinoma), KYSE150 (oesophageal squamous carcinoma) and H1299 (non-small cell lung cancer). In all cell lines detected, knockdown of OTUD6A decreased the protein level of CDC6, while the mRNA level was not affected (Fig. 5b and Supplementary Fig. 5a-e). We further established stable OTUD6A overexpressed UMUC3 and 786-O cells. Consistent with findings in OTUD6A knockdown cancer cells, overexpression of OTUD6A increased the protein level but not the mRNA level of CDC6 in UMUC3 and 786-O cells (Fig. 5c and Supplementary Fig. 5f, g).

The Cell Counting Kit-8 (CCK8), colony formation and EdU incorporation assays showed that knockdown of OTUD6A inhibited the proliferation of all the cancer cells detected (Fig. 5d, e and Supplementary Fig. 5h-u). Consistent with these findings, overexpression of wild-type OTUD6A increased the cancer cell proliferation (Fig. 5f, g and Supplementary Fig. 6a-h). However, overexpression of OTUD6A-C-terminus, which could not interact with CDC6, lost its ability to promote cell proliferation (Supplementary Fig. 6f-h). To confirm the role of OTUD6A in cancer cell proliferation regulation in vivo, we subsequently utilized subcutaneous xenograft mouse models. Knockdown of OTUD6A in T24 and 786-O cells significantly inhibited tumour growth (Fig. 5h-j and Supplementary Fig. 6i-k). The decrease in tumour growth coincided with a reduction in the protein levels of Ki-67 and CDC6 (Fig. 5k and Supplementary Fig. 6l). Moreover, the protein but not the mRNA level of CDC6 was decreased in OTUD6A-knockdown T24 tumours (Supplementary Fig. 6m, n). Consistent with these findings, overexpression of OTUD6A accelerated tumour growth and upregulated CDC6 level in UMUC3 and 786-O cells in athymic mice (Supplementary Fig. 6o-v).

We next evaluated the role of CDC6 in OTUD6A-regulated tumorigenicity. Similar to the observations in OTUD6A knockdown and overexpressed cancer cells, knockdown of CDC6 inhibited while overexpression of CDC6 promoted cancer cell proliferation (Supplementary Fig. 7a-k). Importantly, ectopic expression of CDC6 effectively reduced the defects in cell proliferation of cancer cells and restored the decreased level of chromatin-bound MCM2 in vitro caused by OTUD6A knockdown (Fig. 5l-n, Supplementary Fig. 7l and Supplementary Fig. 8a-l). Moreover, ectopic expression of CDC6 effectively reversed the OTUD6A knockdown-mediated tumour growth inhibition in vivo (Fig. 5o, p and Supplementary Fig. 8m, n). In contrast, overexpression of OTUD6A has no effect on cell proliferation of CDC6 knockdown cells (Fig. 5q and Supplementary Fig. 8o-x). Collectively, these results indicated that OTUD6A promotes the tumorigenicity of human cancer cells by upregulating CDC6.

To confirm that OTUD6A acts as an oncogene in the process of tumorigenesis, we employed the BBN-induced BCa mouse model, which is the most common approach for exploring the mechanism of BCa tumorigenesis [35]. N-butyl-N-(3-carboxybutyl) nitrosamine (BCPN) is a major oxidative metabolite of chemical carcinogen BBN in the urine which has mutagenic function and induces bladder carcinogenesis [36]. Individual mice were sacrificed at every 1 month until 6 months and examined for urothelial tumours. Similar to the effects observed in a previous study [35], 12 weeks of BBN treatment resulted in the development of carcinoma in situ (CIS), and 20 weeks of BBN treatment resulted in the development of muscle-invasive BCa (Supplementary Fig. 9a-d). Immunohistochemical (IHC) staining showed that the OTUD6A, CDC6 and Ki-67 protein levels increased with prolonged BBN treatment (Supplementary Fig. 9e-g), demonstrating that OTUD6A and CDC6 might play roles in BCa tumorigenesis. Notably, knockout of OTUD6A resulted in less bladder tumorigenesis, lower malignancy and a lower CDC6 protein level upon BBN treatment (Fig. 5r-t), indicating that knockout of OTUD6A inhibited BCa tumorigenesis and CDC6 expression induced by chemical carcinogen.

### OTUD6A decreases sensitivity to chemotherapy via the CDC6-ATR-Chk1 pathway

**Fig. 6:**
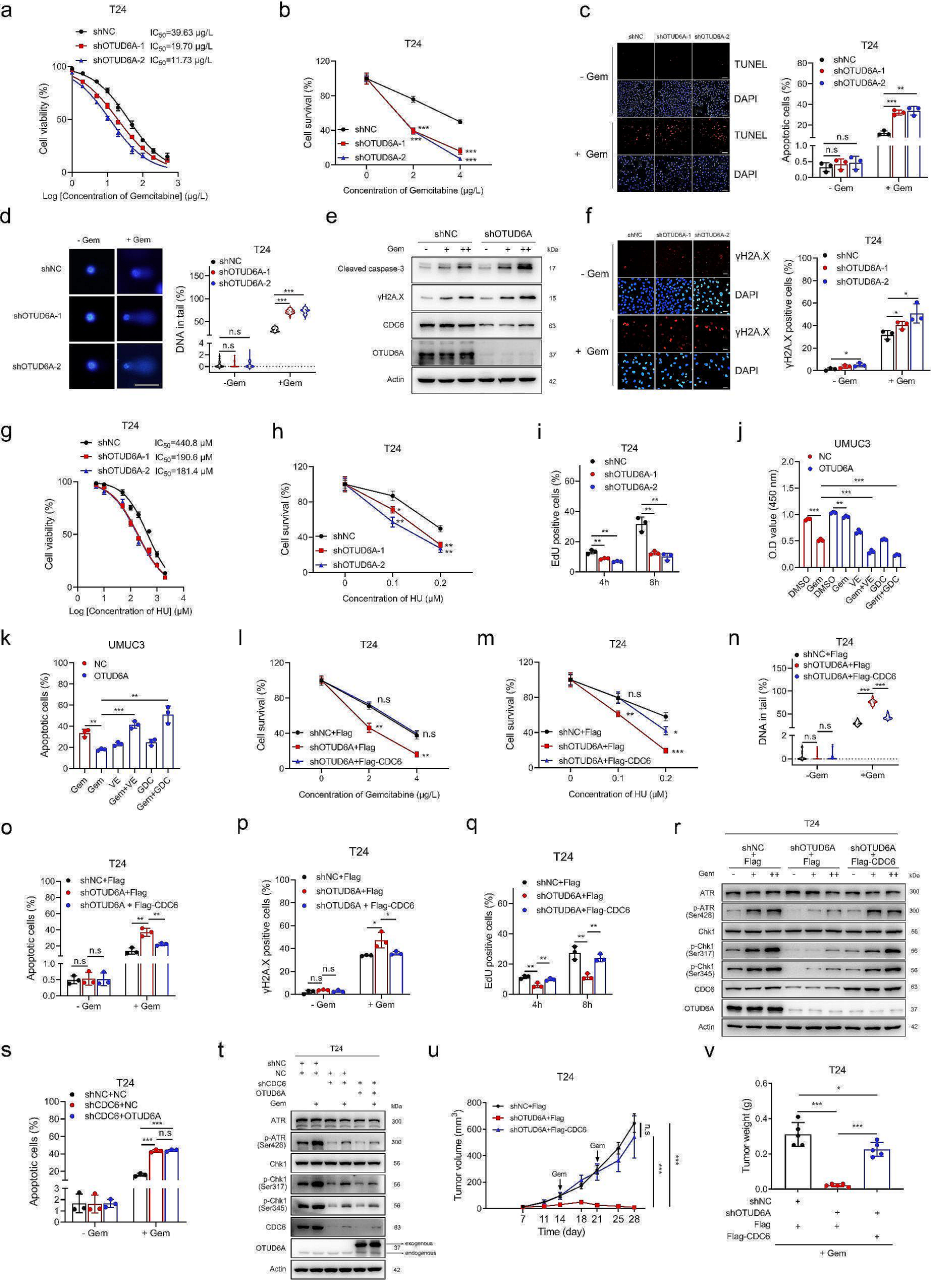
OTUD6A decreases sensitivity to chemotherapy via the CDC6-ATR-Chk1 pathway. **a**, The cell viability of the indicated T24 cells was determined after 48 h of continuous exposure to multiple concentrations of gemcitabine. The IC_50_ value was defined as the concentration causing a 50% decrease in cell viability. The IC_50_ values were estimated by nonlinear regression using a variable Hill slope model. **b**, The indicated T24 cells were treated with different concentrations of gemcitabine. Cell survival was determined by colony formation assays. **c**, Apoptosis was measured by TUNEL assays in the indicated T24 cells treated with or without 20 µg/L gemcitabine for 48 h. Representative images are shown (left). Scale bars, 50 μm. **d**, The amount of DNA strand breaks was quantified by alkaline comet assays in the indicated T24 cells treated with or without 20 µg/L gemcitabine for 48 h. Representative images are shown (left). Scale bars, 20 μm. **e**, The indicated T24 cells were treated with different concentrations of gemcitabine. Cleaved caspase-3 and γH2A.X protein levels were determined by Western blotting. **f**, The γH2A.X protein level was measured by immunofluorescence staining in the indicated T24 cells treated with or without 20 µg/L gemcitabine for 48 h. Representative immunofluorescence images are shown (left). Scale bars, 20 μm. **g**, The cell viability of the indicated T24 cells was determined after 48 h of continuous exposure to multiple concentrations of hydroxyurea (HU). **h**, The indicated T24 cells were treated with different concentrations of HU. Cell survival was determined by colony formation assays. **i**, Recovery of DNA replication activity was quantified by replication reinitiation assays. The indicated T24 cells were treated with 2 mM HU for 24 h and released into fresh medium for 4 h and 8 h. **j, k**, The effects of ATR and Chk1 inhibitors (VE-821 and GDC-0575) on regulating the sensitivity of OTUD6A-overexpressing UMUC3 cells to gemcitabine was determined by CCK8 (**j**) and TUNEL (**k**) assays. **l**, **m**, The indicated T24 cells were treated with different concentrations of gemcitabine (**l**) and HU (**m**). Cell survival was determined by colony formation assays. **n**, The amount of DNA strand breaks was quantified by alkaline comet assays in the indicated T24 cells treated with or without 20 µg/L gemcitabine for 48 h. **o**, Apoptosis was measured by TUNEL assays in the indicated T24 cells treated with or without 20 µg/L gemcitabine for 48 h. **p**, The γH2A.X protein level was measured by immunofluorescence staining in the indicated T24 cells treated with or without 20 µg/L gemcitabine for 48 h. **q**, Recovery of DNA replication activity was quantified by replication reinitiation assays. The indicated T24 cells were treated with 2 mM HU for 24 h and released into fresh medium for 4 h and 8 h. **r**, ATR-Chk1 pathway protein levels in the indicated T24 cells treated with 20 µg/L gemcitabine for 6 h were determined by Western blotting. **s**, Apoptosis was measured by TUNEL assays in the indicated T24 cells treated with or without 20 µg/L gemcitabine for 48 h. **t**, ATR-Chk1 pathway protein levels in the indicated T24 cells treated with 20 µg/L gemcitabine for 6 h were determined by Western blotting. **u**, Growth curves of the indicated subcutaneous T24 tumours treated with 50 mg/kg gemcitabine are shown. **v**, The indicated subcutaneous T24 tumours were weighed. All quantitative analyses were based on three independent experiments. The error bars indicate the SDs. *P < 0.05, **P < 0.01, ***P < 0.001, n.s. not significant, based on two-tailed Student’s *t* test

Enhancement of the DDR is one of the most important mechanisms of cellular chemoresistance [37, 38]. CDC6 is reported to be an important factor in promoting DDR activation [39, 40]. Given that OTUD6A stabilizes the CDC6 protein, we next explored whether OTUD6A also functions in the DDR and the response to chemotherapy. Knockdown of OTUD6A caused hypersensitivity to gemcitabine and methotrexate in different cancer cell lines detected (Fig. 6a, b and Supplementary Fig. 10a-d). Moreover, the number of apoptotic cells was increased in OTUD6A-knockdown T24 cells treated with gemcitabine, not in untreated cells (Fig. 6c). The results of alkaline comet assays showed that the comet tailing was not different between OTUD6A-knockdown and control T24 cells without gemcitabine treatment. However, comet tailing was significantly increased in OTUD6A-knockdown T24 cells after treatment with gemcitabine (Fig. 6d). The level of cleaved caspase-3 and γH2A.X, a marker of DDR activation [41], was also increased in OTUD6A-knockdown T24 cells exposed to gemcitabine (Fig. 6e, f). In contrast, OTUD6A overexpression increased the chemoresistance of UMUC3 cells to gemcitabine and methotrexate (Supplementary Fig. 10e, f). Together, these data indicated that OTUD6A decreases the chemosensitivity of BCa cells.

To confirm the role of OTUD6A in the DDR, we induced DNA damage with HU. Knockdown of OTUD6A increased but overexpression of OTUD6A decreased the sensitivity of BCa cells to HU (Fig. 6g, h and Supplementary Fig. 10g, h). Immunofluorescence staining of γH2A.X also showed that the level of DNA damage was increased in OTUD6A-knockdown T24 cells treated with HU (Supplementary Fig. 10i). We next assessed the role of OTUD6A in DNA replication reinitiation after HU treatment, and the results showed that the DNA replication reinitiation rate was decreased in OTUD6A-knockdown T24 cells after release from HU (Fig. 6i and Supplementary Fig. 10j), suggesting that inhibition of OTUD6A increases DNA damage and reduces DNA repair caused by HU in BCa cells.

The ATR-Chk1 pathway is a key signalling axis driving the DDR [2], and CDC6 has been identified as a critical regulator of ATR-Chk1 activation [2, 8, 9, 42]. We proposed that OTUD6A might regulate the sensitivity of BCa cells to chemotherapy by activating the ATR-Chk1 pathway. Knockdown of OTUD6A decreased the levels of phosphorylated ATR and Chk1 but not Chk2 in T24 cells (Supplementary Fig. 10k), while OTUD6A overexpression increased the levels of phosphorylated ATR and Chk1 in T24 and UMUC3 cells (Supplementary Fig. 10l, m). Moreover, knockdown of OTUD6A inhibited gemcitabine-induced ATR and Chk1 phosphorylation (Supplementary Fig. 10n). Notably, treatment with the ATR inhibitor VE-821 and the Chk1 inhibitor GDC-0575 abrogated the decreased gemcitabine sensitivity induced by overexpression of OTUD6A in UMUC3 cells (Fig. 6j, k and Supplementary Fig. 10o-q). Furthermore, knockdown of ATR results in more apoptotic cells and DNA damage with or without gemcitabine treatment (Supplementary Fig. 10r, s). Knockdown of ATR also reduced Chk1 phosphorylation levels (Supplementary Fig. 10t). And knockdown of ATR and ATR inhibition caused a decrease in ATR and Chk1 activation upon gemcitabine treatment in UMUC3 cells (Supplementary Fig. 10u, v).

We further evaluated whether OTUD6A regulates chemosensitivity through modulation of CDC6. Similar to the results obtained in OTUD6A-knockdown cells, knockdown of CDC6 increased the sensitivity of cancer cells to anticancer chemotherapeutic drugs and HU (Supplementary Fig. 11a-e). Moreover, knockdown of CDC6 resulted in more DNA damage induced by gemcitabine (Supplementary Fig. 11f). Similar to that of OTUD6A overexpression, CDC6 overexpression decreased the chemosensitivity and promoted gemcitabine-induced ATR and Chk1 activation in UMUC3 cells (Supplementary Fig. 11g-k). Notably, ectopic expression of CDC6 rescued the hypersensitivity to gemcitabine, methotrexate and HU caused by OTUD6A knockdown (Fig. 6l, m, Supplementary Fig. 10c, d and Supplementary Fig. 12a-e). The increases in DNA damage and apoptosis induced by OTUD6A knockdown were also significantly ameliorated by CDC6 overexpression in T24 cells treated with gemcitabine and HU (Fig. 6n-p and Supplementary Fig. 12f-i). Moreover, CDC6 overexpression effectively restored the decrease in DNA replication reinitiation and ATR and Chk1 activation in gemcitabine-treated cells caused by OTUD6A knockdown (Fig. 6q, r and Supplementary Fig. 12j). In contrast, ectopic expression of OTUD6A could not ameliorate the increased sensitivity and the decreased ATR and Chk1 activation to HU or gemcitabine treatment caused by CDC6 knockdown (Fig. 6s, t, Supplementary Fig. 11d, e and Supplementary Fig. 12k-o).

Tumour xenograft models were used to further examine the role of the OTUD6A-CDC6 axis in regulating sensitivity to chemotherapy in vivo. Consistent with the in vitro results, enhanced chemosensitivity to gemcitabine and increased DNA damage induced by gemcitabine were observed in OTUD6A-knockdown T24 tumours (Fig. 6u, v and Supplementary Fig. 12p, q). Consistent with the observation from in vitro experiments, overexpression of CDC6 rescued the increased chemosensitivity caused by OTUD6A knockdown in gemcitabine-treated T24 tumours (Fig. 6u, v and Supplementary Fig. 12p, q). Taken together, these data demonstrated that OTUD6A promotes CDC6-ATR-Chk1 signalling pathway activity to confer chemoresistance on tumour cells.

### OTUD6A level is correlated with the level of the CDC6 protein in cancer cells

**Fig. 7:**
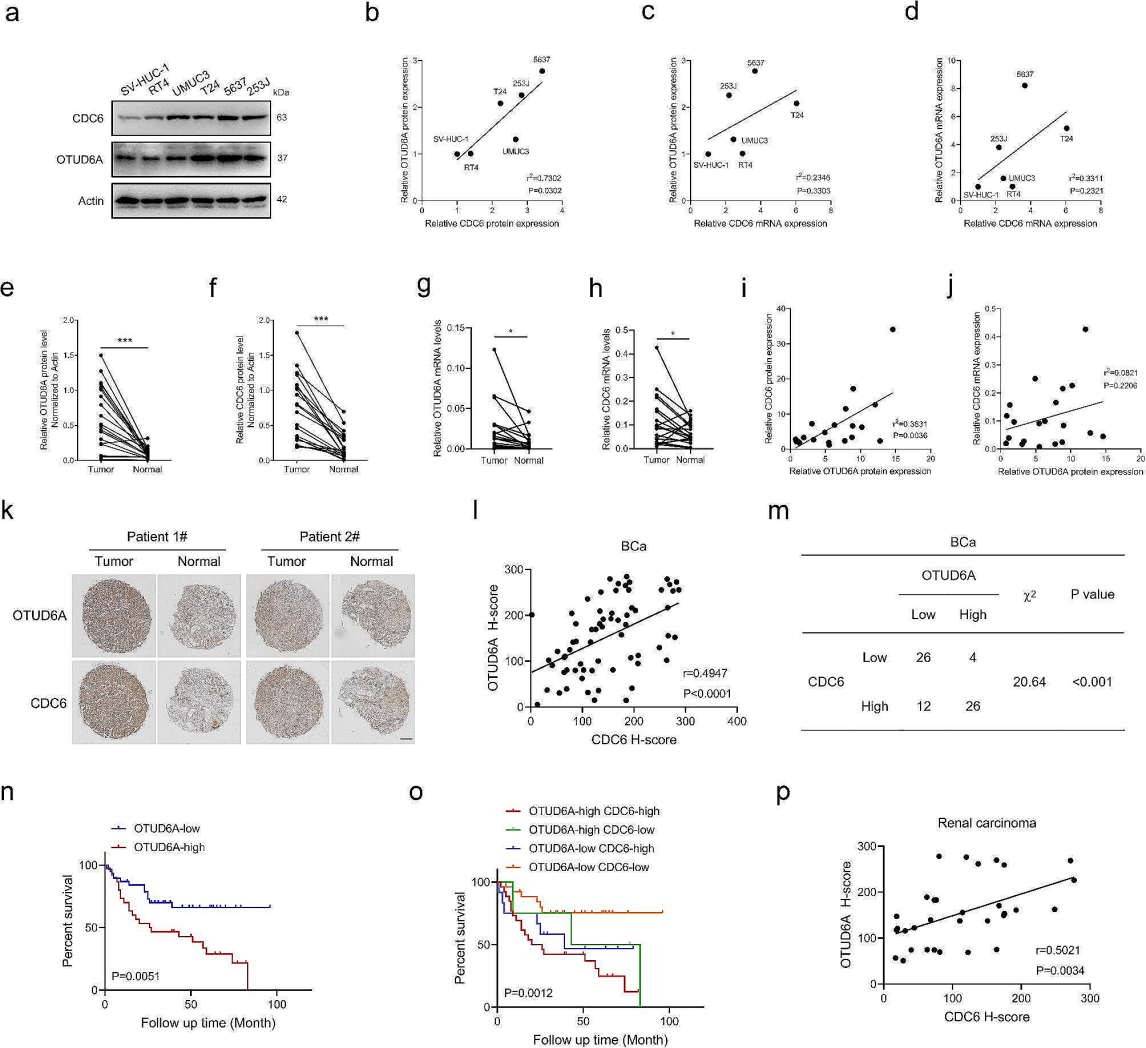
CDC6 protein level is correlated with the OTUD6A protein level in tumour tissue. **a**, The protein levels of OTUD6A and CDC6 in the indicated cells were determined by Western blotting. **b-d**, The correlation between OTUD6A and CDC6 levels in the indicated cells was assessed by Pearson correlation analysis. OTUD6A and CDC6 protein levels (**b**); OTUD6A protein and CDC6 mRNA levels (**c**); OTUD6A and CDC6 mRNA levels (**d**). **e**, **f**, The intensities of the OTUD6A (**e**) and CDC6 (**f**) bands in BCa tissues were quantified and compared with those in matched normal tissues. Data were analysed using two-tailed paired Student’s *t* test. **g**, **h**, Quantitative PCR analysis of OTUD6A (**g**) and CDC6 (**h**) mRNA level in BCa tissues compared with matched normal tissues. Data were analysed using two-tailed paired Student’s *t* test. **i**, The correlation between OTUD6A and CDC6 protein levels in BCa tissues was assessed by Pearson correlation analysis. **j**, The correlation between OTUD6A protein and CDC6 mRNA levels in BCa tissues was assessed by Pearson correlation analysis. **k**, Representative IHC images indicating OTUD6A and CDC6 expression in the tissue microarray are shown. Scale bar, 500 μm. **l**, The correlation between OTUD6A and CDC6 protein levels in the BCa tissue microarray was assessed by Pearson correlation analysis. **m**, The correlation between OTUD6A and CDC6 protein levels in the BCa tissue microarray was assessed by the chi-square test. **n**, Kaplan-Meier curves of overall survival for patients with BCa stratified by the OTUD6A expression level in the tissue microarray are shown. Data were analysed using the log-rank test. **o**, Kaplan-Meier curves of overall survival for patients with BCa stratified by OTUD6A and CDC6 expression levels in the tissue microarray are shown (the numbers of patients with high and low OTUD6A and CDC6 expression are shown in Fig. 7m). Data were analysed using the log-rank test. **p**, The correlation between OTUD6A and CDC6 protein levels in the renal carcinoma tissue microarray was assessed by Pearson correlation analysis. All quantitative analyses were based on three independent experiments. The error bars indicate the SDs. *P < 0.05, ***P < 0.001

We then determined whether OTUD6A-mediated upregulation of CDC6 is of potential clinical significance. A positive correlation between the OTUD6A and CDC6 protein levels was observed in BCa cell lines, whereas no correlation was found between the CDC6 mRNA level and the OTUD6A protein or mRNA level (Fig. 7a-d). We further examined the expression level of OTUD6A and CDC6 in 20 fresh human BCa tissues and matched adjacent normal bladder tissues. Both the protein and mRNA levels of OTUD6A and CDC6 were higher in BCa tissues than in the matched normal bladder tissues (Fig. 7e-h and Supplementary Fig. 13a). CDC6 protein levels in BCa tissues were inconsistent with the CDC6 mRNA levels, indicating the important role of posttranscriptional regulation in CDC6 protein level (Supplementary Fig. 13b). Importantly, CDC6 protein level was positively correlated with OTUD6A protein level in BCa tissues (Fig. 7i). However, there was no correlation between the OTUD6A protein and CDC6 mRNA levels in BCa tissues (Fig. 7j). We then performed IHC staining of OTUD6A and CDC6 in a BCa tissue microarray, and the results confirmed that the protein levels of OTUD6A and CDC6 were highly consistent in BCa tissues (Fig. 7k-m and Supplementary Fig. 13c). Patients bearing BCa tumours with relatively high levels of OTUD6A or CDC6 showed poorer overall survival (OS) outcomes (Fig. 7n and Supplementary Fig. 13d). Notably, combined high level of OTUD6A and CDC6 was more strongly correlated with worse outcomes in BCa patients, and patients with high OTUD6A and low CDC6 level had better OS outcomes than patients with low OTUD6A and high CDC6 level (Fig. 7o). However, no discernible difference in OS between patients with high OTUD6A and high CDC6 level and patients with low OTUD6A and high CDC6 level was observed (Fig. 7o). The protein levels of OTUD6A and CDC6 were also consistent in renal carcinoma tissues (Fig. 7p and Supplementary Fig. 13e). Collectively, these data demonstrated that the OTUD6A-CDC6 axis is preferentially activated in BCa and renal carcinoma and that its level is correlated with poor survival in BCa patients. Therefore, OTUD6A and CDC6 may serve as a new set of prognostic biomarkers in BCa patients.

## Discussion

**Fig. 8:**
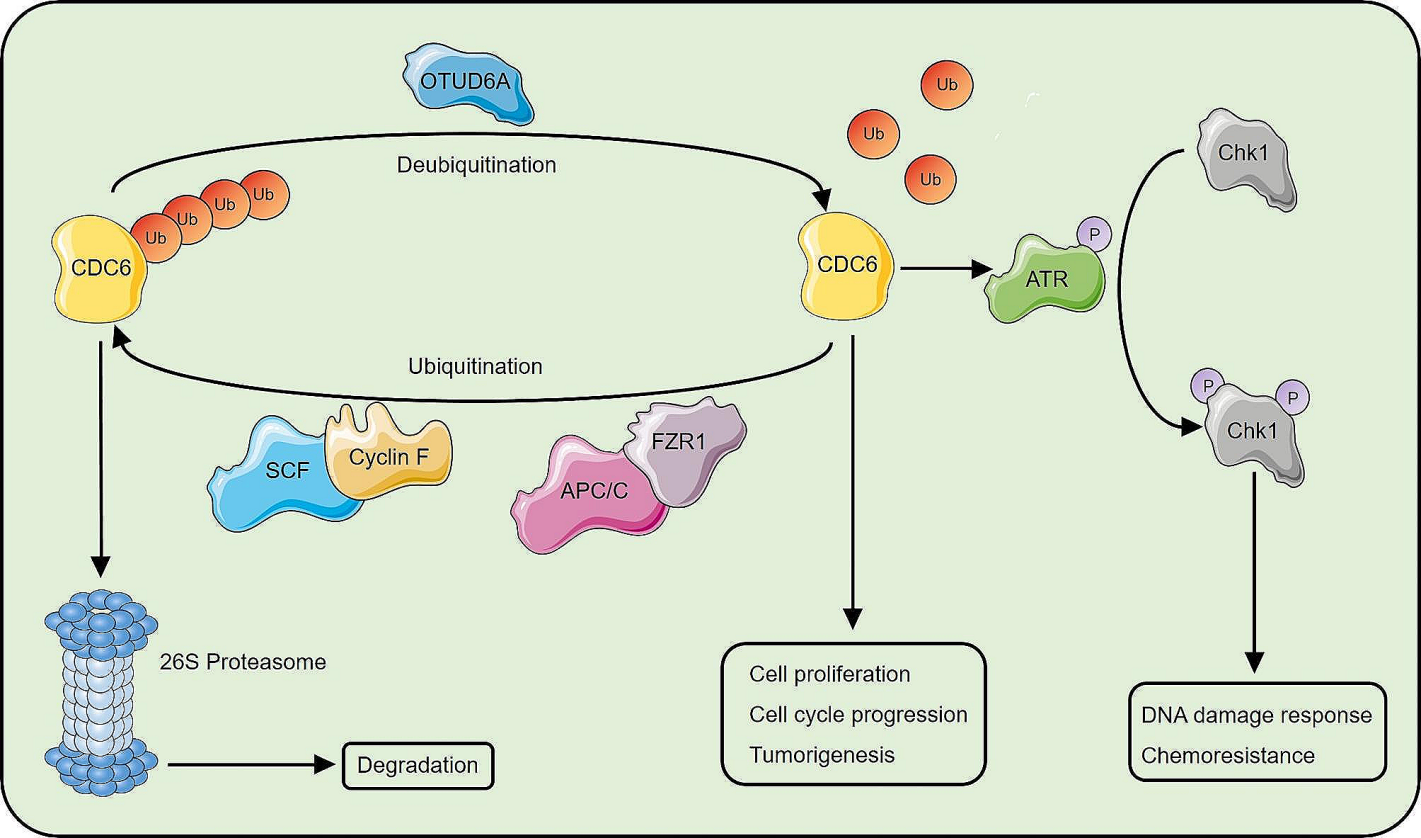
A schematic model showing the mechanism by which the OTUD6A-CDC6 axis regulates cell proliferation and the DNA damage response. OTUD6A directly binds to and deubiquitinates CDC6, reverses CDC6 degradation mediated by APC/C-CDH1 and SCF-Cyclin F, and consequently promotes cell proliferation and induces chemoresistance

Precise regulation of the cell cycle is essential for living organisms. As CDC6 is one of the key factors in the cell cycle, its expression and localization during the cell cycle are regulated by multiple posttranslational regulatory pathways [14, 20, 21, 25, 32, 43]. The CDC6 protein can be degraded by the ubiquitin‒proteasome system mediated by the E3 ubiquitin ligases APC/C-CDH1, SCF-CDC4, CRL4-CDT2 and SCF-Cyclin F [16, 19–21, 25]. Ubiquitination can be reversed by DUBs that cleave ubiquitin chains from the substrate protein [22]. Through a screening of DUBs, we found that OTUD6A interacts with CDC6 and increases the CDC6 protein level by promoting the stability of CDC6 through removing polyubiquitin chains, whose attachment was mediated by SCF-Cyclin F and APC/C-CDH1 (Fig. 8). CDC6 expression fluctuates during the cell cycle [32]. Our results confirmed that the changes in the levels of CDC6 protein caused by OTUD6A were not results from changes in the cell cycle. The OTUD6A protein level pattern is similar to that of CDC6 in cell cycle progression. Importantly, the interaction pattern of OTUD6A and CDC6 is in line with the CDC6 protein level during cell cycle progression. Moreover, OTUD6A translocates into the cytoplasm in the S phase, indicating that the cytoplasmic translocation of OTUD6A permits the degradation of CDC6 and prevents the re-replication of DNA. Therefore, OTUD6A cooperates with the ubiquitination system to regulate CDC6 protein level during cell cycle progression.

OTUD6A belongs to the ovarian tumour protease (OTU) family and the OTUD subfamily, which contains OTUD1, OTUD2, OTUD3, OTUD4, OTUD5, OTUD6A, OTUD6B and ALG13. The OTUD subfamily members have emerged as important factors in various human diseases and pathological processes. OTUD1 binds to and deubiquitinates apoptosis-inducing factor (AIF), thereby activating both caspase-independent and caspase-dependent apoptotic signalling [44]. OTUD3 inhibits the activation of the AKT pathway by deubiquitinating PTEN and suppresses tumour progression in breast cancer (BC), colon cancer and cervical cancer [45]. OTUD6B has the highest homology with OTUD6A among the OTUD subfamily members. Two OTUD6B splice isoforms recognize different substrates and have completely opposite effects on non-small cell lung cancer cell proliferation [46]. Mice with homozygous *Otud6b* knockout show embryonic lethality, and biallelic mutations cause human mental retardation syndrome [47]. However, the physiological function of OTUD6A remains to be fully elucidated. Here, we demonstrated that OTUD6A plays a vital role in organism growth. Depletion of OTUD6A results in downregulation of CDC6 protein level and proliferation defects in MEFs. Importantly, similar to Meier-Gorlin syndrome in humans, which can be caused by CDC6 mutation, depletion of OTUD6A in mice causes postnatal growth retardation, suggesting that OTUD6A is required for normal development at least partially through maintenance of CDC6 protein stability.

CDC6 is overexpressed and acts as a proto-oncogene in various cancers [42, 48]. Here, we found that OTUD6A upregulated CDC6 protein level in several types of cancer cells. OTUD6A was previously reported to be an oncogene in colorectal cancer (CRC), PCa and BC, and OTUD6A is overexpressed in CRC, PCa and BC tissues [24, 49–51]. OTUD6A interacts with and deubiquitinates Drp1 to enhance mitochondrial fission [49]. OTUD6A deubiquitinates Brg1 and AR to promote PCa progression, and knockdown of OTUD6A suppresses prostate tumorigenesis by reversing Myc-driven metabolic remodelling [24, 50]. In addition, OTUD6A inhibits TopBP1 ubiquitination by disrupting the interaction between TopBP1 and UBR5 and promotes tumour cell resistance to chemoradiotherapy [51]. Despite several substrates of OTUD6A were identified recently, herein, we identified the first deubiquitinase, OTUD6A, targeting CDC6 for deubiquitination. OTUD6A protein level is upregulated during the progression of BCa and is positively associated with CDC6 level induced by BBN treatment in mice. Depletion of OTUD6A inhibits CDC6 expression as well as BBN-induced BCa tumorigenesis and tumour progression. We further showed that OTUD6A, through upregulation of CDC6, promotes the proliferation of multiple types of human tumour cells in vitro and tumour growth in vivo, suggesting the importance of OTUD6A-CDC6 axis in these cancer cells. Moreover, both the OTUD6A and CDC6 protein levels are increased in BCa and renal carcinoma tumour tissues, and the trend in OTUD6A protein level is consistent with the trend in CDC6 protein level in both BCa and renal carcinoma tissues. In addition, BCa patients with high CDC6 and high OTUD6A protein levels have the worst OS outcomes, and patients with low CDC6 and high OTUD6A protein levels exhibited improved OS compared with patients with high CDC6 and low OTUD6A protein levels, suggesting that OTUD6A exerts its oncogenic effects mainly through upregulation of CDC6 in BCa cells. These findings broaden our understanding of OTUD6A under both physiological and pathological conditions.

Genome instability elicits ongoing DNA damage and DNA replication stress [1, 3]. The DDR is a complex suite of mechanisms that counteract threats to genomic integrity [52]. In response to DNA damage induced by chemotherapeutic drugs and ionizing radiation, the DDR is activated, leading to suppression of DNA damage and promotion of cancer cell survival [53]. DNA damage is first recognized by molecular “sensors”, most notably those in the ATR/Chk1- and ATM/Chk2-mediated signalling pathways [54], which are hyperactivated in various cancers and associated with chemoresistance and poor prognosis [54–56]. CDC6 is also involved in the DDR. CDC6 silencing suppresses ATR function and increases genomic instability and DNA damage-induced cell death. In addition, CDC6 promotes the DDR by activating the ATR-Chk1 pathway in PCa and BCa [9, 42]. We showed that DNA damage promotes the binding of OTUD6A to CDC6, subsequently upregulating CDC6 protein level, activating the ATR-Chk1 pathway and resulting in chemoresistance (Fig. 8). Our results confirmed the synergistic effect of ATR/Chk1 inhibitors and gemcitabine in OTUD6A-overexpressing BCa cells, suggesting the possible beneficial effects of targeting ATR/Chk1 in cancers with high expression of OTUD6A and CDC6.

## Conclusions

In conclusion, our study identifies OTUD6A as a novel positive regulator of CDC6, which plays an important role in the cell cycle, cell proliferation, organism growth, tumorigenesis, the DDR and chemosensitivity, providing global insight into the physiological and pathological functions of OTUD6A. Our data suggest that the OTUD6A-CDC6 axis may be exploited as a promising clinical target for cancer therapy. In addition, the combination of ATR/Chk1 inhibitors with chemotherapy can be effective in patients with high OTUD6A expression.

### Electronic supplementary material

Below is the link to the electronic supplementary material.

Supplementary Material 1

## Data Availability

The proteomics data are uploaded on PRIDE database (https://www.ebi.ac.uk/pride/). Data are available via ProteomeXchange with identifier PXD042935. All data needed to evaluate the conclusions in the paper are present in the main text and the supplementary materials.

## Abbreviations

DDR: DNA damage response
ORC: origin recognition complex
CDC6: cell division cycle 6
CDT1: chromatin licensing and DNA replication factor 1
MCM2: minichromosome maintenance protein 2
pre-RC: pre-replicative complex
ATR: ataxia telangiectasia and Rad3-related protein
MGS: Meier-Gorlin syndrome
E2F: early region 2 binding factor
AR: androgen receptor
FOXM1: forkhead box M1
UPS: ubiquitin‒proteasome system
DUB: deubiquitinase
OTUD6A: OTU domain-containing 6 A
IF: Immunofluorescence
shRNA: short hairpin RNA
CKO: conditional *Otud6a* knockout
MEF: mouse embryonic fibroblast
co-IP: Coimmunoprecipitation
HU: hydroxyurea
KOG: EuKaryotic Orthologous Groups
GO: Gene Ontology
BCa: bladder cancer
CCK8: Cell Counting Kit-8
BCPN: N-butyl-N-(3-carboxybutyl) nitrosamine
CIS: carcinoma in situ.
IHC: Immunohistochemical
OS: overall survival
AIF: apoptosis-inducing factor
BC: breast cancer
CRC: colorectal cancer
