# Supplementary material for: Deubiquitination of CDC6 by OTUD6A promotes tumour progression and chemoresistance
Source: Mol Cancer. 2024 Apr 29;23:86. doi: 10.1186/s12943-024-01996-y (PMC11057083; doi:10.1186/s12943-024-01996-y)
Supplement: Supplementary file 1 — Supplementary Material 1 [file 12943_2024_1996_MOESM1_ESM.docx]

**Supplementary materials**

**Methods**

**Plasmid DNA transfection and RNA interference**

Cells at 70% confluence were transfected with the indicated plasmid DNA using Lipofectamine^TM^ 3000 transfection reagent (Invitrogen, L3000015) according to the manufacturer’s protocol. siRNA duplex oligomers of ATR and the negative control siRNA were synthesized by Shandong Gene&Bio Co. Ltd (Shandong, China) . Cells at 30-40% confluence were transfected with X-tremeGENE siRNA Transfection Reagent (Roche, 4476093001) following the manufacturer’s protocol.

**Lentiviral packaging and infection**

To generate lentiviral shRNA constructs targeting human CDC6, lentiviral vectors carrying a shRNA targeting human CDC6 were purchased from GeneChem Inc. (Shanghai, China). The CDC6 shRNA/negative control shRNA (shNC) and the pMD2.G and psPAX2 plasmids were cotransfected into HEK293T cells for 48 h, and the viral supernatant was filtered through a 0.45 μm filter (Millipore).

For the construction of cells with stable OTUD6A- and CDC6-knockdown/overexpression cells were infected with lentivirus as indicated. OTUD6A knockdown/overexpression and CDC6 overexpression lentiviruses were purchased from Genechem Inc. (Shanghai, China). Lentiviruses containing empty plasmids (NC) and shNC were used as controls.

Viruses were used to infect cells in the presence of polybrene for 48 h, and the cells were cultured in medium containing puromycin (1 mg/mL) for 5 days. Stably transduced cells resistant to puromycin were identified and verified by qRT-PCR and Western blot analysis.

**Subcellular fractionation**

Subcellular fractionation was performed using a Subcellular Protein Fractionation Kit (Thermo Fisher Scientific, 78840) following the manufacturer’s instructions. Briefly, cells were harvested, CEB buffer was added for 10 min, and the supernatant was taken as the cytoplasmic protein fraction after centrifugation. Soluble nuclear proteins were collected after adding NEB buffer. The remaining sample was treated with micrococcal nuclease, which released chromatin-bound nuclear proteins into the supernatant. The supernatant was boiled with SDS-PAGE loading buffer at 99 ℃ for 10 min and analysed by Western blotting.

**Chromatin fractionation**

Chromatin-bound protein was extracted using the Chromatin Extraction Kit (Abcam ab117152) according to the manufacturer’s instructions. Briefly, treated cells were harvested in working lysis buffer on ice for 10 min and centrifuged at 5,000 rpm for 5 min. Then, the supernatant was removed, and working extraction buffer was added on ice for another 10 min, followed by sonication (10 s, 20 cycles). The samples were centrifuged at 12,000 rpm for 10 min at 4 ℃. The samples were boiled with SDS-PAGE loading buffer at 99 ℃ for 10 min and analyzed by Western blotting.

**Proteomic analysis**

The Flag-OTUD6A or Flag vector was transfected into 293 cells for 24 h. No less than 10 million cells were harvested using a cell scraper and were sent to PTM BIO company for 4D label-free quantitative proteomic analysis (Hangzhou, China). Data are uploaded on PRIDE database (https://www.ebi.ac.uk/pride/) and available via ProteomeXchange with identifier PXD042935. Differentially expressed proteins were subjected to further Gene Ontology (GO) and EuKaryotic Orthologous Groups (KOG) analysis.

**Mass spectrometry**

HEK293 cells were transfected with the Flag-OTUD6A or Flag vector, and cell lysates were subjected to IP with an anti-Flag antibody. The immunoprecipitates were separated on 10% gels, which were then stained with Coomassie Brilliant Blue solution (Beyotime, P0017F). The gels were cut into small pieces, destained with 100 mM NH_4_HCO_3_/30% ACN and dried in a vacuum centrifuge. The in-gel proteins were incubated with dithiothreitol (10 mM DTT/100 mM NH_4_HCO_3_) at 56 ℃ for 30 min and were then rinsed in iodoacetamide (200 mM IAA/100 mM NH_4_HCO_3_) in the dark at room temperature for 20 min. The gels were washed sequentially with 100 mM NH_4_HCO_3_ and ACN. The gels were digested with 5 ng/μL trypsin (Promega) at 4 ℃ for 1 h and incubated with 50 mM NH_4_HCO_3_ at 37 ℃ for 18 h. Peptides were extracted three times with 60% ACN/0.1% TFA by ultrasonic shearing. The extracts were pooled and dried completely in a vacuum centrifuge. Peptides were recovered by solid-phase extraction using C18 ZipTips (Millipore) and resuspended in 0.1% TFA/50% ACN for analysis by LC-MS/MS (Orbitrap Elite, Thermo Scientific). All raw files were processed using proteinDiscover (version 1.4, Thermo Scientific) for database searching. MS/MS spectra were searched against the UniProtKB/SwissProt human database (downloaded on December 29, 2017).

**Bimolecular fluorescent complimentary (BiFC) assay**

293 and U2OS cells were transfected with Flag-YFP-N terminus (Flag-YN), HA-YFP-C terminus (HA-YC), Flag-YFP-N terminus-OTUD6A (YN-OTUD6A) or HA-YFP-C terminus-CDC6 (YC-CDC6) plasmids. An additional 24 h later, the cells were stained with Hoechst 33342 and imaged with a confocal microscope (LSM880 NLO, Zeiss, Germany). YFP and Hoechst 33342 were excited by a 514 nm Argon and 405 nm diode laser respectively, and their fluorescence was detected at 527 nm and 461 nm, respectively.

**GST pull-down assay**

The GST-tagged full-length OTUD6A, OTUD6A-N-terminal (1-145 aa) and OTUD6A-C-terminal (129-288 aa) constructs were transfected into *E. coli* BL21 (DE3), and protein expression was induced with 200 μM isopropyl β-D-1-thiogalactopyranoside (IPTG) at 20 ℃ overnight. The bacteria were collected and lysed in lysis buffer (PBS + 0.1 μM PMSF + 0.1 μM DTT) under sonication for 20 min at 4 ℃. The lysate was centrifuged at 15,000 rpm for 15 min at 4 ℃, and the supernatant was incubated with 200 μL of glutathione-sepharose (Cytiva, 17513201) for 2 h at 4 ℃. Then, the beads were washed with lysis buffer four times and incubated with total protein lysate from HEK293 cells for 2 h at 4 ℃. Then, the beads were washed with Western and IP buffer four times, followed by Western blotting.

**Protein half-life assay**

Cells were transfected with the indicated plasmids for 24 h in individual experiments. Then, the cells were treated with cycloheximide (CHX, 20 μg/mL) for the indicated durations before harvesting. Stable OTUD6A-knockdown cells were treated only with CHX (20 μg/mL) for the indicated durations before harvesting.

***In vivo* deubiquitination assay**

The indicated plasmids were transfected into HEK293 cells for 24 h. After incubation with 20 μM MG132 for 6 h, the cells were harvested and lysed in Western and IP buffer with protease inhibitors. Then, an IP assay was used to assess ubiquitination levels by immunoblotting with the indicated antibodies.

***In vitro* deubiquitination assay**

HEK293 cells were transfected together with the Myc-CDC6 and HA-Ub expression plasmids for 48 h. The cells were treated with MG132 for 6 h and lysed. The lysate was purified with an anti-Myc affinity gel, eluted with Myc peptide and subjected to HA affinity gel purification to enrich HA-Ub-conjugated CDC6 (Ub-CDC6). HEK293 cells were transfected with Flag-OTUD6A, and the cell lysates were incubated with anti-Flag affinity gel for 2 h. The beads were then washed five times with lysis buffer. Recombinant OTUD6A proteins were subsequently incubated with Ub-CDC6 in deubiquitination reaction buffer (50 mM Tris-HCl (pH 8.0), 50 mM NaCl, 1 mM EDTA, 10 mM DTT, 5% glycerol) at 37 ℃ for 30 min. The ubiquitination status of CDC6 was analysed by Western blotting.

**Cell cycle synchronization assay**

U2OS cells were treated with 2 mM thymidine for 24 h, washed twice with PBS, and released for 4 h in fresh medium before being treated with 100 ng/mL nocodazole in DMEM for 16 h to obtain a prometaphase population by a “shake-off” procedure. For synchronization in G1/S phase, HeLa cells were cultured in the presence of 2.5 mM thymidine for 16 h, washed two times with PBS, and released in fresh medium without thymidine for 8 h. After another 16 h of culture in medium containing thymidine, the cells were washed two times with PBS and released in fresh RPMI-1640 medium.

**Flow cytometry**

Cells were fixed with 70% ethanol and stained with propidium iodide (PI) using standard methods. In brief, cells were harvested using trypsin, centrifuged at 3,000 rpm for 3 min, and washed once with cold PBS. The cells were fixed with cold 70% ethanol at -20 ℃ overnight. The fixed cells were washed with PBS once and then incubated with 500 μL of PBS containing 100 μg/mL RNase A (Solarbio, R1030) and 50 μg/mL PI (Solarbio, P8080) at room temperature in the dark for 30 minutes. The cell cycle distribution was analysed by using a BD Biosciences FACScan II cytometer (Becton Dickinson, San Jose, CA, USA).

**CCK8, colony formation and EdU incorporation assays**

CCK8, colony formation, and EdU incorporation assays were performed as previously described. Briefly, for the CCK-8 assay, 10 μL of CCK-8 (NCM Biotech, C6005) was added to each well for 1 h. The optical density (OD) values were determined at 450 nm. For half-maximal inhibitory concentration (IC_50_) measurement, cells were plated in 96-well dishes and treated with multiple concentrations of various drugs for 48 h. Then, a CCK8 assay was used, and the OD was determined at 450 nm. GraphPad Prism (version 8.0.2, GraphPad Software, CA, USA) software was used to calculate cell viability. The cell viability (%) is calculated as follows: [OD value (drug treatment)-OD value (blank)/OD value (average control)-OD value (blank)]×100).

For the colony formation assay, eight hundred cells per well were seeded in 6-well plates and cultured for 7-14 days. The cell colonies were fixed with 4% paraformaldehyde, stained with crystal violet and counted.

The EdU (5-ethynyl-2′-deoxyuridine) incorporation assay was performed using the Cell-Light™ EdU Apollo 567 In Vitro Kit (Ribobio, C10310-1, Guangzhou, China) according to the manufacturer’s instructions. Briefly, cells were cultured on coverslips overnight, EdU was added to the medium for 30 min, and the cells were fixed with 4% paraformaldehyde. After fixation, the cells were incubated with EdU staining solution for 1 h and subsequently stained with DAPI. The EdU-labelled cells were imaged with a fluorescence microscope (BX51, Olympus Life Science, Tokyo, Japan).

**Replication reinitiation assays**

Cells were treated with HU (2 mM) for 24 h to arrest at S phase which lost the ability to undergo DNA replication. Subsequently, the cells were released in fresh medium for 0 h, 4 h and 8 h. EdU was added for 30 minutes before harvesting. Then the EdU incorporation assay was performed as described above. The EdU positive cells represent the DNA replication reinitiation cells.

**Tissue specimens**

A total of 20 paired human fresh BCa tumour and matched adjacent normal tissues were obtained at Hospital. All samples were surgical specimens collected from patients with no neoadjuvant chemotherapy or radiotherapy. Informed consent was obtained from all patients or their relatives before surgery. This study was approved by the Medical Ethics Committee, Shandong University of Clinical Medicine. Bladder tissue microarrays containing 68 BCa tissues and 40 normal bladder tissues were obtained from Shanghai Outdo Biotech (Cat No. HBlaU108Su01, Shanghai, China). Renal tissue microarrays containing 32 renal carcinoma tissues and 15 normal renal tissues were obtained from Shanghai Outdo Biotech (Cat No. HKidCRC030PG01 and HKidE030PG02, Shanghai, China).

**Terminal deoxynucleotidyl transferase-mediated dUTP-fluorescein nick end labelling (TUNEL) assay**

The TUNEL assay was performed using a one-step TUNEL Apoptosis Assay Kit (Beyotime, C1089) according to the manufacturer’s instructions. Cells were cultured on coverslips and exposed to gemcitabine for 48 h. The cells were fixed with 4% paraformaldehyde for 30 min, incubated with TUNEL mixture for 1 h and subsequently stained with DAPI. The TUNEL-labelled cells were imaged with a fluorescence microscope (BX51, Olympus Life Science, Tokyo, Japan).

**Alkaline comet assay**

The alkaline comet assay was performed using the Single Cell Gel Electrophoresis Assay Kit (Trevigen, 4250-050-K) according to the manufacturer’s protocol. Briefly, cells were treated with gemcitabine for 48 h, harvested and suspended in ice-cold PBS (Ca^2+^ and Mg^2+^ free). Then, molten LMAgarose (at 37 °C) was combined with the cells at a ratio of 1:10 (v/v), and 50 μL was immediately pipetted onto a CometSlide™. The slides were incubated at 4 °C in the dark for 10 min. Then, the slides were immersed in lysis solution overnight at 4 °C prior to incubation in Alkaline Unwinding Solution for 60 min at 4 °C. Alkaline single-cell gel electrophoresis was performed, and the slides were stained with DAPI and imaged with a fluorescence microscope (BX51, Olympus Life Science, Tokyo, Japan). % DNA in tail was used to quantify the extent of DNA damage using CometScore software (version 1.5).

**MEF cell isolation**

The indicated mice were killed at 13.5-14.5 days of gestation. Mouse embryos were dissected, and the embryonic internal organs, head, tail, and limbs were removed. The carcasses were cut into small pieces. The tissues were incubated in 0.15% trypsin/EDTA solution for 15 min at 37 °C and vortexed every 5 min prior to centrifugation at 1500 rpm for 5 min. The supernatant was removed, and the cells were resuspended and cultured in RPMI-1640 medium. Primary MEFs were used for a maximum of five passages.


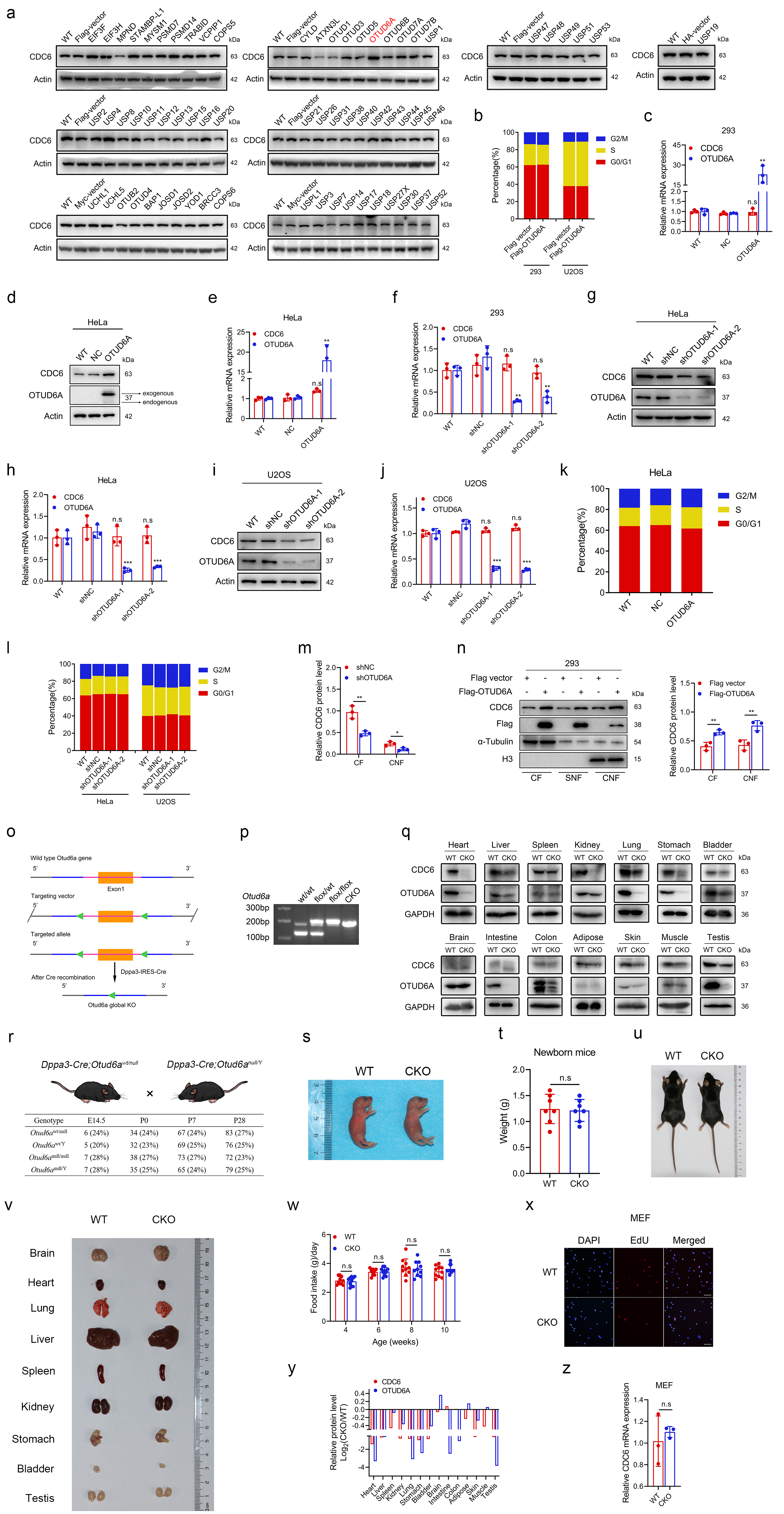


**Supplementary Fig. 1 OTUD6A upregulates CDC6 protein expression.** **a**, HEK293 (293) cells were transfected with the indicated DUBs individually, and the endogenous CDC6 expression level was measured by Western blotting. **b**, Cell cycle distribution of asynchronous 293 and U2OS cells transiently transfected with indicated vectors. **c**, CDC6 and OTUD6A expression levels were measured in the indicated HeLa cells by qPCR; the levels in WT cells were set as 1. **d, e**, CDC6 and OTUD6A expression levels were measured in the indicated HeLa cells by Western blotting (**d**) and qPCR (**e**); the levels in WT cells were set as 1. **f**, CDC6 and OTUD6A expression levels were measured in the indicated 293 cells by qPCR; the levels in WT cells were set as 1. **g, h**, CDC6 and OTUD6A expression levels were measured in the indicated HeLa cells by Western blotting (**g**) and qPCR (**h**); the levels in WT cells were set as 1. **i**, **j**, CDC6 and OTUD6A expression levels were measured in the indicated U2OS cells by Western blotting (**i**) and qPCR (**j**); the levels in WT cells were set as 1. **k, l**, The cell cycle distribution of asynchronous OTUD6A stably overexpressed HeLa (**k**), as well as asynchronous OTUD6A stably knockdown HeLa and U2OS (**l**) cells were analyzed by flow cytometry. **m**, The relative intensities of the CDC6 bands in CF (CDC6/α-Tubulin) and CNF (CDC6/H3) were quantified from three independent repeated Western blotting analysis. The optical density (OD) of protein bands was analyzed by ImageJ. **n**, Cytoplasmic, soluble nuclear and chromatin-bound nuclear fractions were extracted from the indicated cells using subcellular fractionation assay and detected by Western blotting (left). The relative intensities of the CDC6 bands in CF (CDC6/α-Tubulin) and CNF (CDC6/H3) were quantified from three independent repeated Western blotting analysis. (right). CF, cytoplasmic fractions; CNF, chromatin-bound nuclear fractions. **o**, The strategy for the generation of global OTUD6A knockout (CKO) mice. Loxp recombination sites (green triangles) were introduced flanking exon 1. **p**, Genotyping of the indicated mice was performed by PCR analysis. **q**, The protein levels of OTUD6A and CDC6 in tissues from OTUD6A WT and CKO littermates (8 weeks old) were measured by Western blotting. **r**, Numbers of mice of each genotype among the progeny of *Dppa3-Cre;Otud6a^wt/null^* female mice and *Dppa3-Cre;Otud6a^null/Y^* male mice. **s**, Representative image of the indicated newborn mice. **t**, Quantitative analysis of body weight between newborn WT (n = 7) and CKO (n = 7) mice. **u**, Representative image of 8-week-old WT and CKO mice. **v**, Representative images of the indicated tissues from WT and CKO littermates (8 weeks old) are shown. **w**, Food intake in 4, 6, 8 and 10 week-old CKO and WT mice (g/day) (n =10 in each group). **x**, Representative images of EdU staining are shown. Scale bars, 50 μm. **y**, Relative OTUD6A and CDC6 protein levels shown in Supplementary Fig. 1q from the indicated tissues of 8-week-old WT and CKO mice. **z**, CDC6 and OTUD6A expression levels were measured in WT and CKO mouse-derived mouse embryonic fibroblasts (MEFs) by qPCR; the levels in WT mice were set as 1. All quantitative analyses were based on three independent experiments. The error bars indicate the SDs. **P* < 0.05, ***P* < 0.01, ****P* < 0.001, n.s. not significant, based on two-tailed Student’s *t* test.


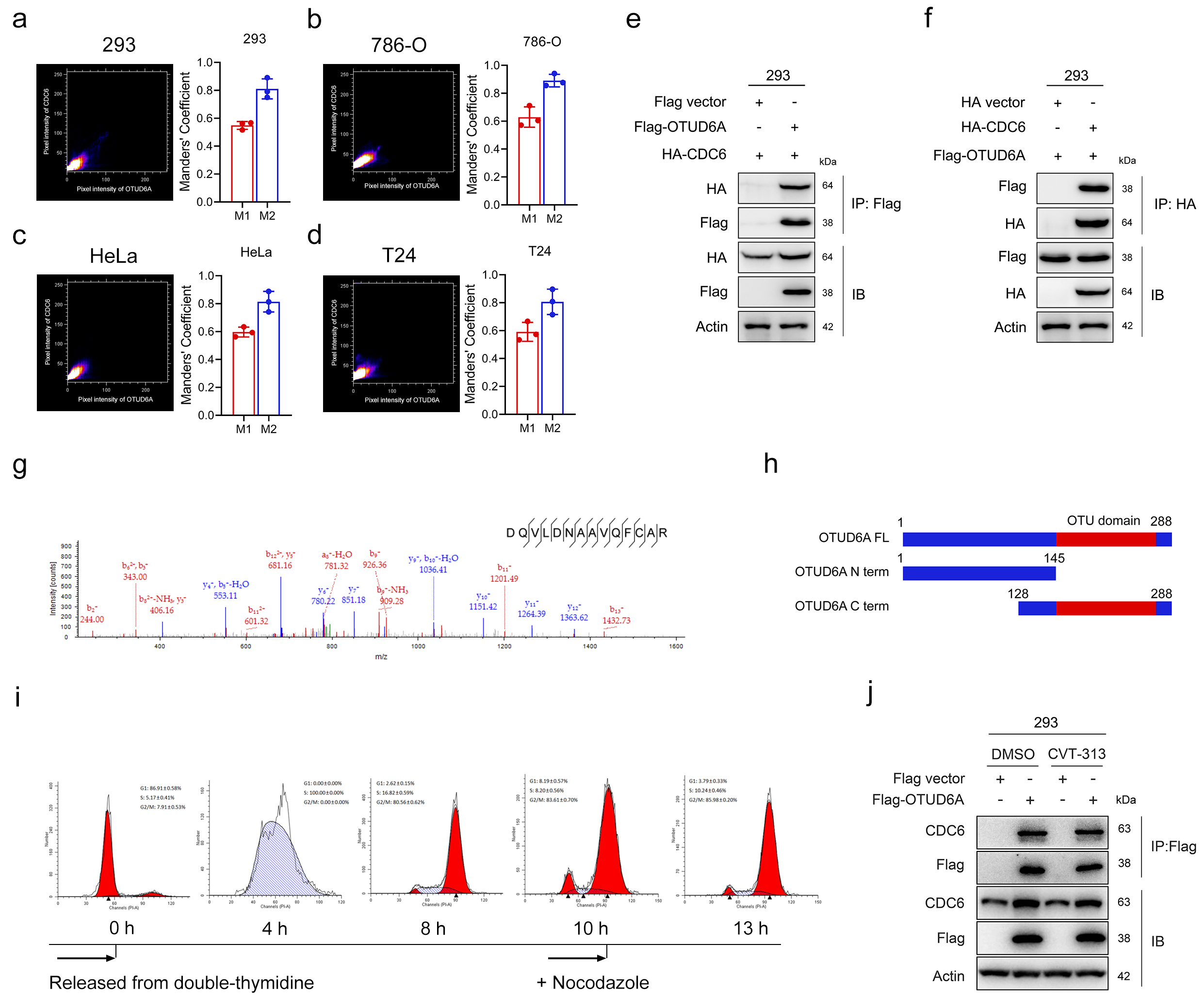


**Supplementary Fig. 2 OTUD6A interacts with CDC6.** **a-d**, The colocalization analysis of OTUD6A and CDC6 in 293 (**a**), 786-O (**b**), HeLa (**c**) and T24 (**d**) cells using Manders’ coefficient analysis. M1 represents the proportion of overlapping pixel intensity of CDC6 and OTUD6A in CDC6 pixel intensity. M2 represents the proportion of overlapping pixel intensity of CDC6 and OTUD6A in OTUD6A pixel intensity. **e, f**, Flag-OTUD6A and HA-CDC6 were cotransfected into 293 cells for 24 h. Whole-cell lysates were prepared and subjected to immunoprecipitation (IP) with anti-Flag (**e**) or anti-HA (**f**) antibodies. The immunoprecipitates were analysed by Western blotting. **g**, Representative fragmentation spectrum of the identified CDC6 peptides by mass spectrometry. **h**, Schematic illustration of OTUD6A constructs. **i**, Representative flow cytometric plots of the cell cycle distribution at the indicated time points. **j**, 293 cells transfected with the indicated vector were treated with CVT-313 (2 mM) or DMSO for 24 h. Whole-cell lysates were prepared and subjected to IP with an anti-Flag antibody. The immunoprecipitates were analysed by Western blotting.


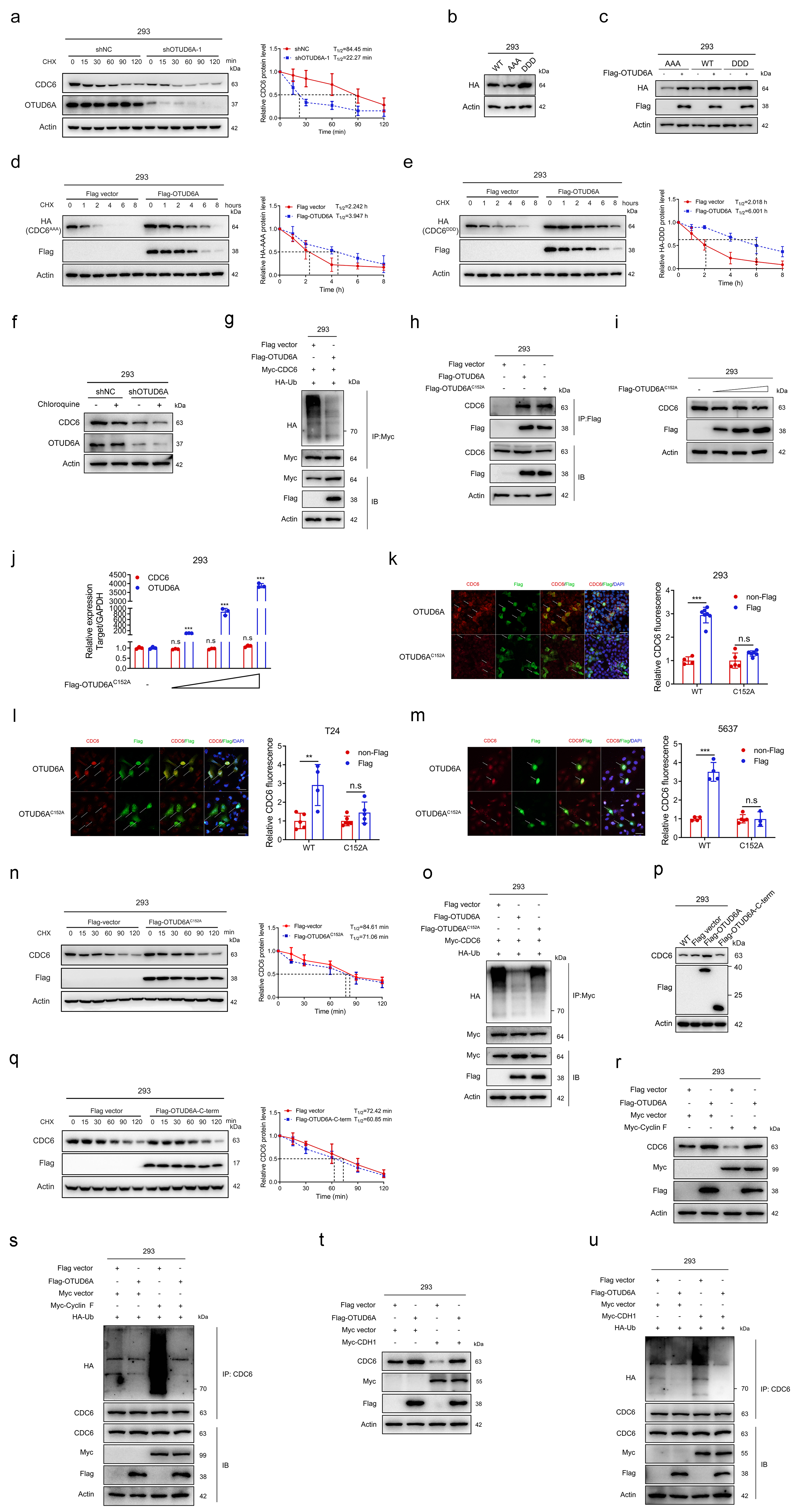


**Supplementary Fig. 3 OTUD6A deubiquitinates CDC6 and promotes CDC6 stability.** **a**, 293 cells with stable OTUD6A knockdown were treated with CHX (20 μg/mL) and harvested at the indicated time points prior to Western blotting (left). The intensities of the CDC6 bands were quantified from three independent repeated Western blotting analysis (right), and the intensity at 0 min was set as 1. **b**, Western blot analysis of lysates from 293 cells transfected with HA-CDC6^WT^, HA-CDC6^AAA^ and HA-CDC6^DDD^. **c**, 293 cells transfected with the indicated vectors were harvested and subjected to Western blotting. **d**, **e**, 293 cells were cotransfected with Flag-OTUD6A or Flag vector with HA-CDC6^AAA^ (**d**) or HA-CDC6^DDD^ (**e**) for 24 h. Cells were treated with CHX and harvested at the indicated time points prior to Western blotting (left). The intensities of the CDC6 bands were quantified from three independent repeated Western blotting analysis (right), and the intensity at 0 h was set as 1. **f**, 293 cells transfected with the indicated vectors were treated with chloroquine (20 μM) or DMSO for 6 h. Cell lysates were prepared, and the CDC6 protein level was measured by Western blotting. **g**, 293 cells transfected with the indicated vectors were treated with MG132 for 6 h. Whole-cell lysates were prepared and subjected to IP with an anti-Myc antibody. The immunoprecipitates were analysed by Western blotting. **h**, 293 cells were transfected with the indicated vectors for 24 h. Whole-cell lysates were prepared and subjected to IP with an anti-Flag antibody. The immunoprecipitates were analysed by Western blotting. **i**, **j**, Increasing amounts of Flag-OTUD6A^C152A^ plasmids were transfected into 293 cells, and the protein levels of endogenous CDC6 and exogenous OTUD6A were determined by Western blotting (**i**). The mRNA levels of CDC6 and OTUD6A were determined by qPCR (**j**), and the levels in Flag vector cells were set as 1. **k-m**, Flag-OTUD6A or Flag-OTUD6A^C152A^ was transfected into 293 (**k**), T24 (**l**) and 5637 (**m**) cells. An additional 24 h later, the cells were fixed and stained as indicated. Representative immunofluorescence images are shown (left). Scale bars, 20 μm. Quantification of the relative fluorescence intensity of CDC6 is shown (right), and the fluorescence intensity of CDC6 in Flag-OTUD6A untransfected cells was set as 1. **n**, 293 cells transfected with the indicated vector were treated with CHX and harvested at the indicated time points prior to Western blotting (left). The intensities of the CDC6 bands were quantified from three independent repeated Western blotting analysis (right), and the intensity at 0 min was set as 1. **o**, 293 cells transfected with the indicated vectors were treated with MG132 for 6 h. Whole-cell lysates were prepared and subjected to IP with an anti-Myc antibody. The immunoprecipitates were analysed by Western blotting. **p**, 293 cells transfected with the indicated plasmids were harvested and subjected to Western blotting. **q**, 293 cells were transfected with indicated vectors for 24 h. Cells were treated with CHX and harvested at the indicated time points prior to Western blotting (left). The intensities of the CDC6 bands were quantified from three independent repeated Western blotting analysis (right), and the intensity at 0 h was set as 1. **r**, 293 cells were cotransfected with Flag-OTUD6A or Flag vector along with Myc-Cyclin F or Myc vector for 24 h, and the indicated protein levels were determined by Western blotting. **s**, 293 cells transfected with the indicated vectors were treated with MG132 for 6 h. Whole-cell lysates were prepared and subjected to IP with an anti-CDC6 antibody. The immunoprecipitates were analysed by Western blotting. **t**, 293 cells were cotransfected with Flag-OTUD6A or Flag vector along with Myc-CDH1 or Myc vector for 24 h, and the indicated protein levels were determined by Western blotting. **u**, 293 cells transfected with the indicated vectors were treated with MG132 for 6 h. Whole-cell lysates were prepared and subjected to IP with an anti-CDC6 antibody. The immunoprecipitates were analysed by Western blotting. All quantitative analyses were based on three independent experiments. The error bars indicate the SDs. ***P* < 0.01, ****P* < 0.001, n.s. not significant, based on two-tailed Student’s *t* test.


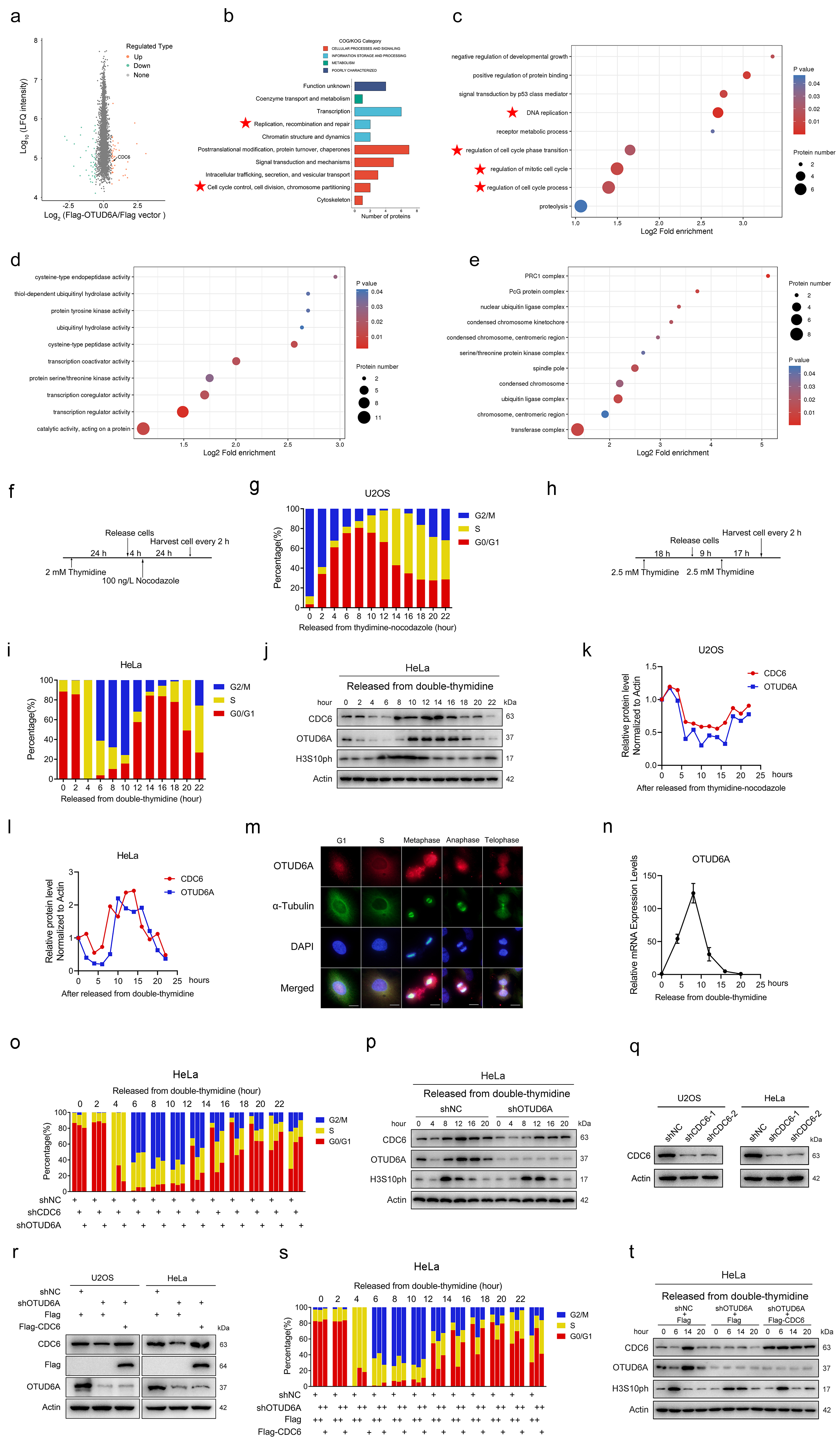


**Supplementary Fig. 4 OTUD6A fluctuates during the cell cycle and regulates cell cycle progression.** **a**, Volcano plot showing the differentially expressed proteins between Flag-OTUD6A and Flag vector groups (fold change > 1.5) based on 4D label-free quantitative proteomics analysis. Significantly downregulated proteins are represented as ‘green’ dots, and significantly upregulated proteins are represented as ‘orange’ dots. **b**, EuKaryotic Orthologous Groups (KOG) analysis of the 41 differentially upregulated proteins in Flag-OTUD6A group from 4D label-free quantitative proteomic analysis. **c-e**, Gene Ontology (GO) analysis of the 41 differentially upregulated proteins in Flag-OTUD6A group from 4D label-free quantitative proteomic analysis in the biological process (**c**), molecular function (**d**) and cellular component (**e**) categories. **f**, A diagram of the procedure for cell cycle synchronization with thymidine and nocodazole is shown. **g**, U2OS cells were synchronized in prometaphase and released into fresh medium. Cells were analysed at the indicated time points by flow cytometry. **h**, A diagram of the process for cell cycle synchronization with a double-thymidine block is shown. **i**, **j**, HeLa cells were synchronized in G1/S phase with a double thymidine block and released into fresh medium. The cells were analysed at the indicated time points by flow cytometry (**i**) and Western blotting (**j**). **k,** **l**, The intensities of the OTUD6A and CDC6 bands in U2OS (**k**) and HeLa (**l**) cells were quantified, and the intensities at 0 h were set as 1. **m**, Representative images of immunofluorescence HeLa cells stained with an anti-OTUD6A antibody (red), α-tubulin antibody (green), and DAPI (blue) are shown. Scale bars, 10 μm. **n**, HeLa cells were synchronized in G1/S phase with a double-thymidine block and released into fresh medium. The cells were analysed at the indicated time points by qPCR. The levels at 0 h were set as 1. **o, p**, The indicated HeLa cells were synchronized in G1/S phase with a double-thymidine block and released into fresh medium. The cells were analysed at the indicated time points by flow cytometry (**o**) and Western blotting (**p**). **q**, The protein expression levels of CDC6 in the indicated cells were determined by Western blotting. **r**, The protein expression levels of OTUD6A and CDC6 in the indicated U2OS and HeLa cells were determined by Western blotting. **s**, **t**, The indicated HeLa cells were synchronized in G1/S phase with a double-thymidine block and released into fresh medium. The cells were analysed at the indicated time points by flow cytometry (**s**) and Western blotting (**t**). All quantitative analyses were based on three independent experiments. The error bars indicate the SDs.


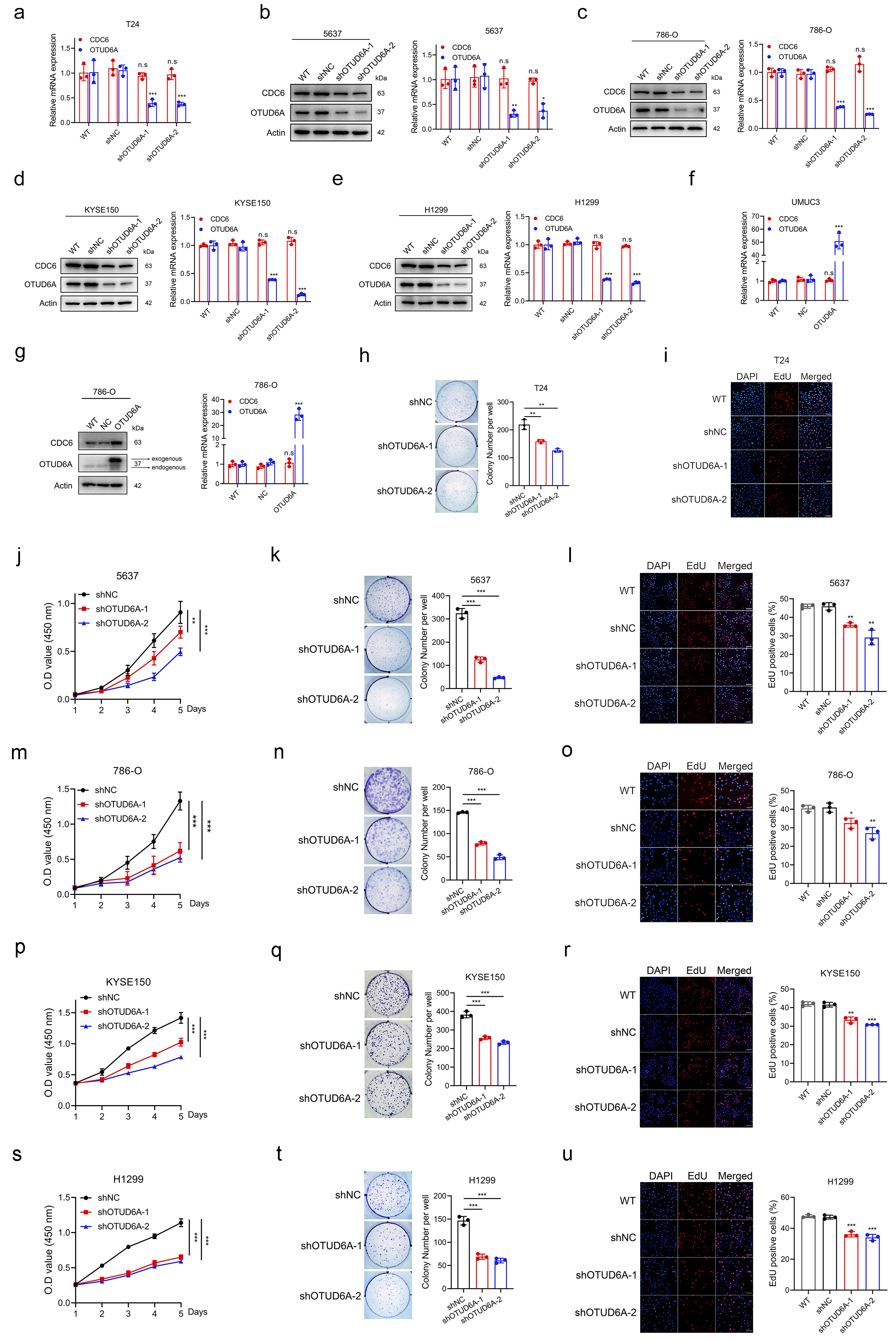


**Supplementary Fig. 5 OTUD6A increases the cancer cell proliferation. a**, CDC6 and OTUD6A expression levels were measured in T24 cells by qPCR; the levels in WT cells were set as 1. **b-e**, CDC6 and OTUD6A expression levels were measured in 5637 (**b**), 786-O (**c**), KYSE150 (**d**) and H1299 (**e**) cells by Western blotting (left) and qPCR (right); the levels in WT cells were set as 1. **f**, CDC6 and OTUD6A expression levels were measured in UMUC3 cells by qPCR; the levels in WT cells were set as 1. **g**, CDC6 and OTUD6A expression levels were measured in 786-O cells by Western blotting (left) and qPCR (right); the levels in WT cells were set as 1. **h, i**, The effect of OTUD6A knockdown on T24 cell proliferation was examined by colony formation assays (**h**) and EdU incorporation assays (**i**). Scale bars, 50 μm. **j-l**, The effect of OTUD6A knockdown on 5637 cell proliferation was examined by CCK8 assays (**j**), colony formation assays (**k**) and EdU incorporation assays (**l**). Scale bars, 50 μm. **m-o**, The effect of OTUD6A knockdown on 786-O cell proliferation was examined by CCK8 assays (**m**), colony formation assays (**n**) and EdU incorporation assays (**o**). Scale bars, 50 μm. **p-r**, The effect of OTUD6A knockdown on KYSE150 cell proliferation was examined by CCK8 assays (**p**), colony formation assays (**q**) and EdU incorporation assays (**r**). Scale bars, 50 μm. **s-u**, The effect of OTUD6A knockdown on H1299 cell proliferation was examined by CCK8 assays (**s**), colony formation assays (**t**) and EdU incorporation assays (**u**). Scale bars, 50 μm. All quantitative analyses were based on three independent experiments. The error bars indicate the SDs. **P* < 0.05, ***P* < 0.01, ****P* < 0.001, n.s. not significant, based on two-tailed Student’s *t* test.


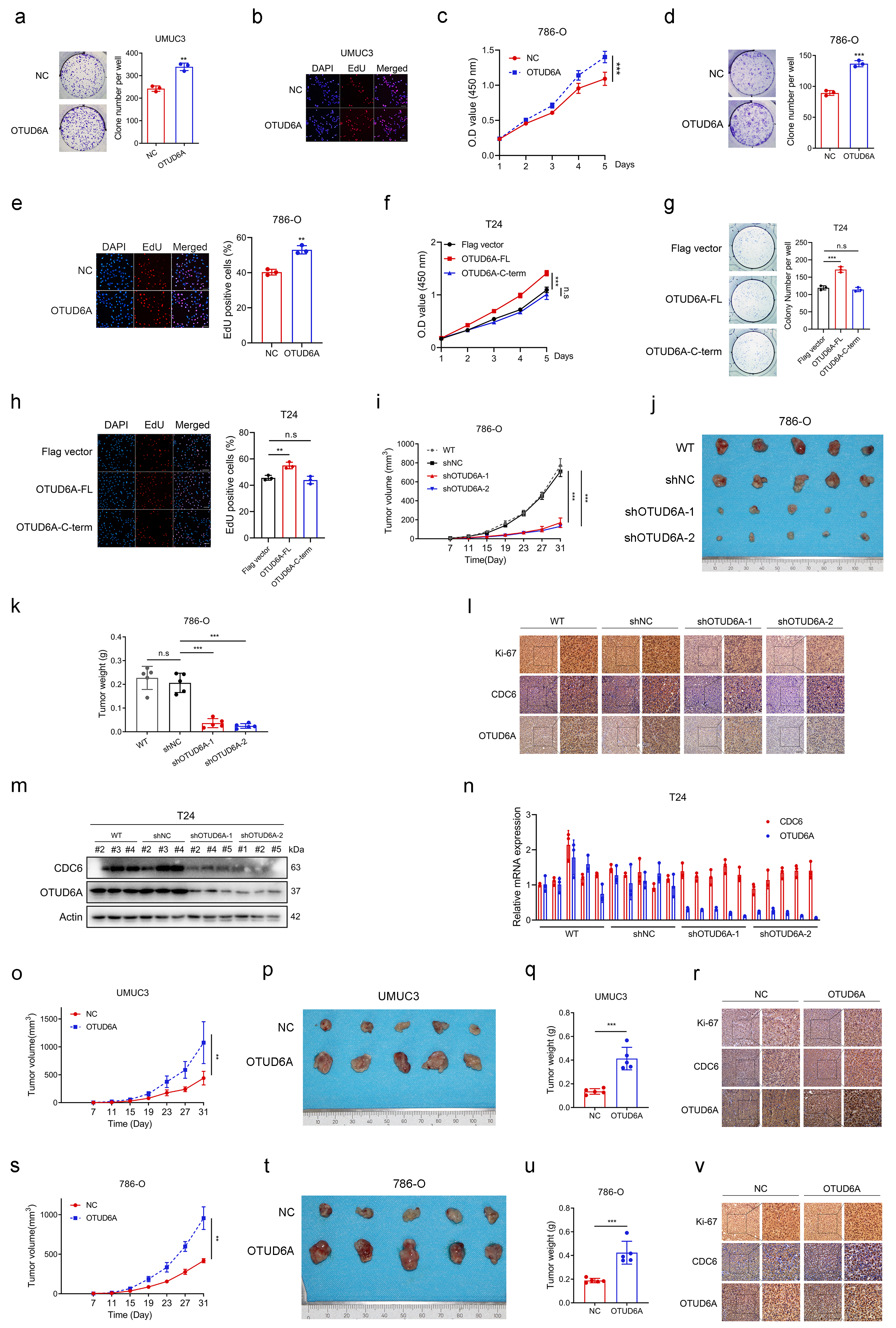


**Supplementary Fig. 6 OTUD6A promotes tumour growth.** **a, b**, The effect of OTUD6A overexpression on UMUC3 cell proliferation was examined by colony formation assays (**a**) and EdU incorporation assays (**b**). Scale bars, 50 μm. **c-e**, The effect of OTUD6A overexpression on 786-O cell proliferation was examined by CCK8 assays (**c**), colony formation assays (**d**) and EdU incorporation assays (**e**). Scale bars, 50 μm. **f-h**, The effect of the C terminus of OTUD6A on T24 cell proliferation was examined by CCK8 assays (**f**), colony formation assays (**g**) and EdU incorporation assays (**h**). Scale bars, 50 μm. **i**, Growth curves of the indicated subcutaneous 786-O tumours are shown. Tumours were measured every 4 days. **j**, An image of subcutaneous tumours formed by the indicated 786-O cells is shown. **k**, The indicated subcutaneous 786-O tumours were weighed. **l**, Representative IHC images indicating Ki-67, CDC6 and OTUD6A expression in the indicated 786-O tumours are shown. Scale bars, 50 μm (left) and 20 μm (right). **m**, **n**, CDC6 and OTUD6A expression levels were measured in the indicated subcutaneous T24 tumours by Western blotting (**m**) and qPCR (**n**). The weight of the first WT tumour was set as 1. **o**, Growth curves of the indicated subcutaneous UMUC3 tumours are shown. Tumours were measured every 4 days. **p**, An image of subcutaneous tumours formed by the indicated UMUC3 cells is shown. **q**, The indicated subcutaneous UMUC3 tumours were weighed. **r**, Representative IHC images indicating Ki-67, CDC6 and OTUD6A expression in the indicated UMUC3 tumours are shown. Scale bars, 50 μm (left) and 20 μm (right). **s**, Growth curves of the indicated subcutaneous 786-O tumours are shown. Tumours were measured every 4 days. **t**, An image of subcutaneous tumours formed by the indicated 786-O cells is shown (right). **u**, The indicated subcutaneous 786-O tumours were weighed. **v**, Representative IHC images indicating Ki-67, CDC6 and OTUD6A expression in the indicated 786-O tumours are shown. Scale bars, 50 μm (left) and 20 μm (right). All quantitative analyses were based on three independent experiments. The error bars indicate the SDs. ***P* < 0.01, ****P* < 0.001, n.s. not significant, based on two-tailed Student’s *t* test.


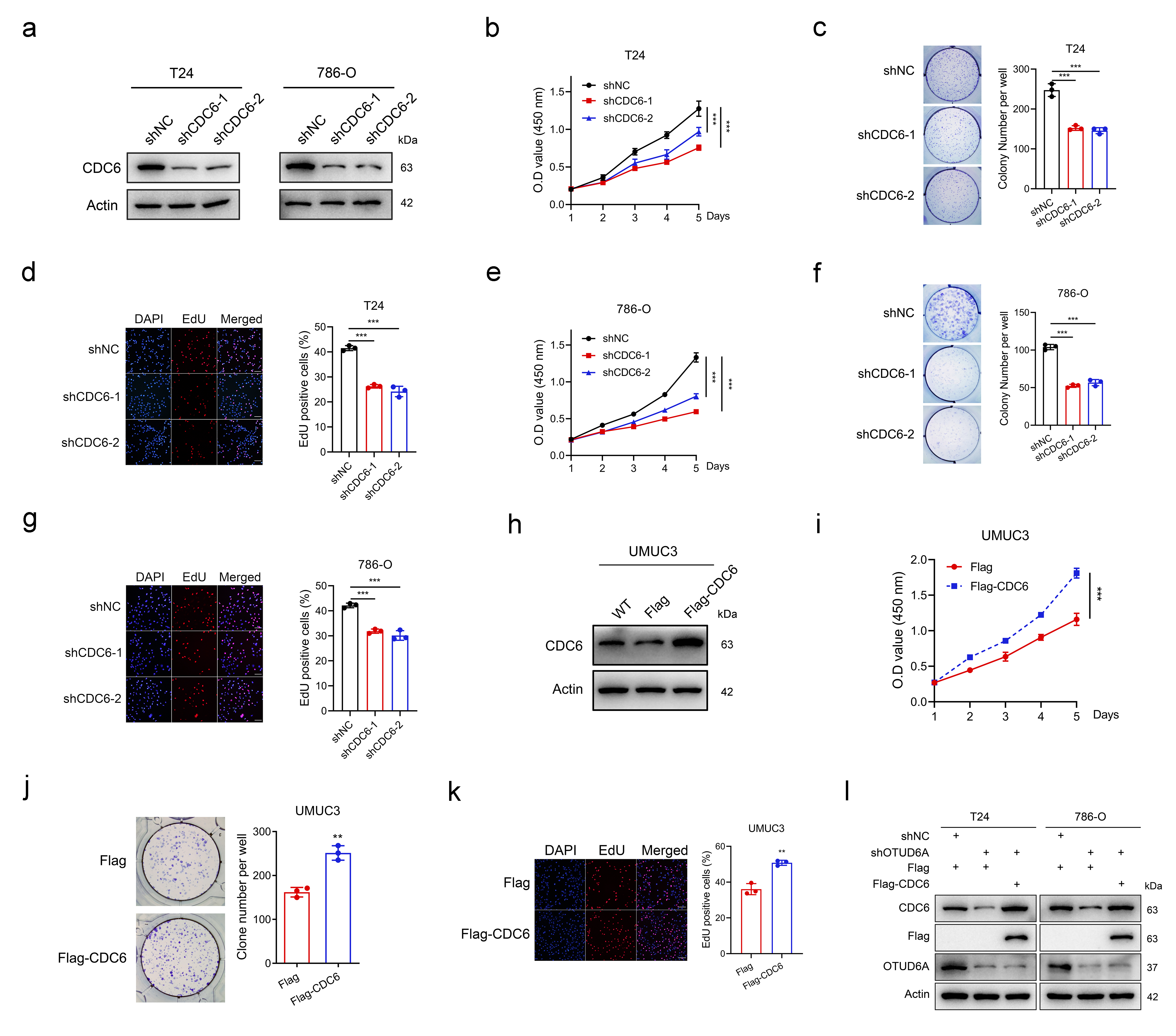


**Supplementary Fig. 7 CDC6 increases the cancer cell proliferation. a,** The protein expression level of CDC6 in the indicated cells was determined by Western blotting. **b-d**, The effect of CDC6 knockdown on T24 cell proliferation was examined by CCK8 assays (**b**), colony formation assays (**c**) and EdU incorporation assays (**d**). Scale bars, 50 μm. **e-g**, The effect of CDC6 knockdown on 786-O cell proliferation was examined by CCK8 assays (**e**), colony formation assays (**f**) and EdU incorporation assays (**g**). Scale bars, 50 μm. **h**, CDC6 expression levels in UMUC3 cell were measured by Western blotting. **i-k**, The effect of CDC6 overexpression on UMUC3 cell proliferation was examined by CCK8 assays (**i**), colony formation assays (**j**) and EdU incorporation assays (**k**). Scale bars, 50 μm. **l**, The protein expression levels of OTUD6A and CDC6 in the indicated T24 and 786-O cells were determined by Western blotting. All quantitative analyses were based on three independent experiments. The error bars indicate the SDs. ***P* < 0.01, ****P* < 0.001, based on two-tailed Student’s *t* test.


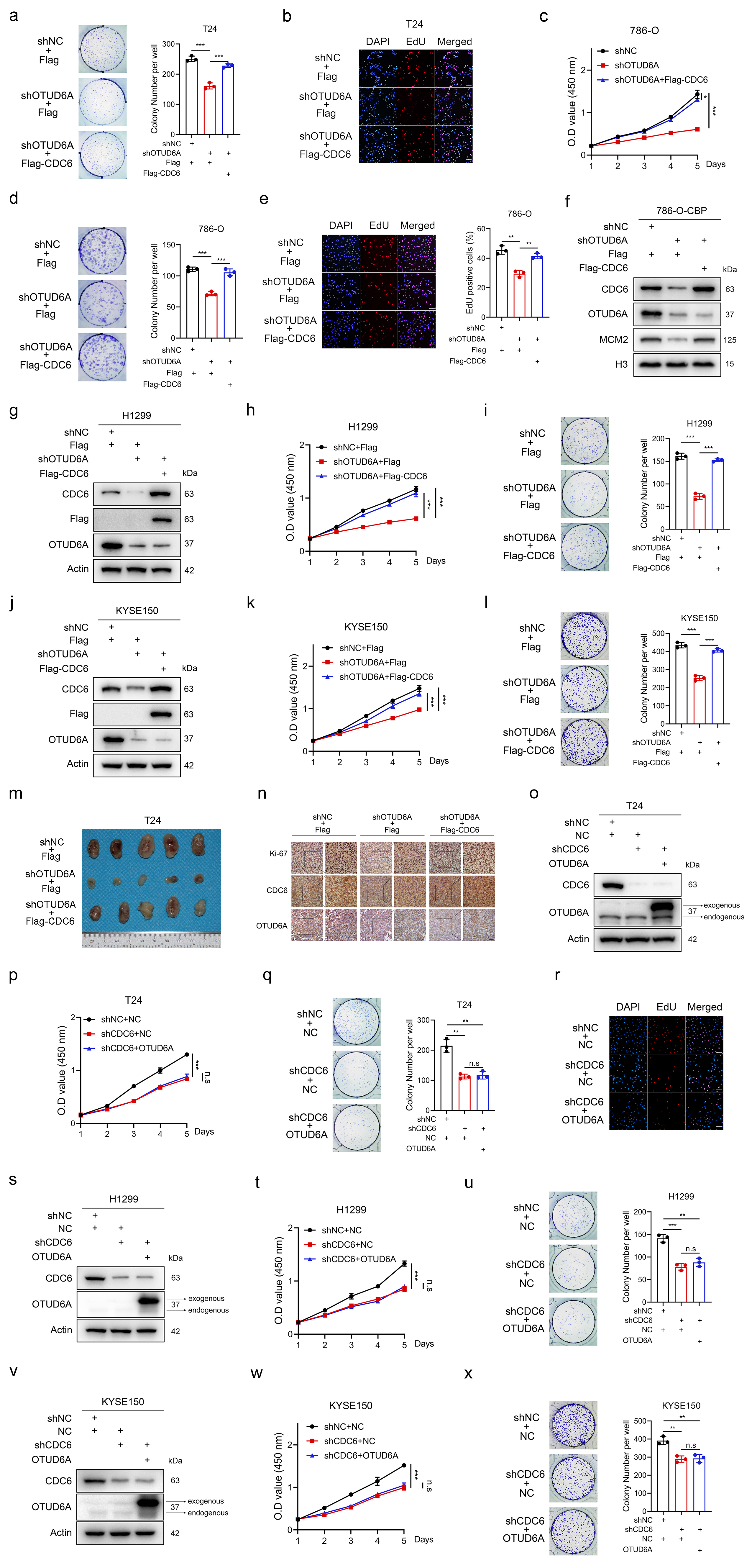


**Supplementary Fig. 8 The OTUD6A-CDC6 axis promotes tumour growth. a, b**, Colony formation assays (**a**) and EdU incorporation assays (**b**) were used to examine proliferation in the indicated T24 cells. Scale bars, 50 μm. **c-e**, CCK8 assays (**c**), colony formation assays (**d**) and EdU incorporation assays (**e**) were used to examine proliferation in the indicated 786-O cells. Scale bars, 50 μm. **f**, Chromatin-bound proteins (CBP) were extracted from the indicated 786-O cells and analysed by Western blotting. **g**, The protein levels of OTUD6A and CDC6 in the indicated H1299 cells were determined by Western blotting. **h, i**, CCK8 assays (**h**) and colony formation assays (**i**) were used to examine the proliferation of the indicated H1299 cells. **j**, The protein levels of OTUD6A and CDC6 in the indicated KYSE150 cells were determined by Western blotting. **k, l**, CCK8 assays (**k**) and colony formation assays (**l**) were used to examine the proliferation of the indicated KYSE150 cells. **m**, Images of subcutaneous tumours formed by the indicated T24 cells are shown. **n**, Representative IHC images indicating Ki-67, CDC6 and OTUD6A expression in the indicated T24 tumours are shown. Scale bars, 50 μm (left) and 20 μm (right). **o**, The protein levels of OTUD6A and CDC6 in the indicated T24 cells were determined by Western blotting. **p-r**, CCK8 assays (**p**), colony formation assays (**q**) and EdU incorporation assays (**r**) were used to examine the proliferation of the indicated T24 cells. Scale bars, 50 μm. **s**, The protein levels of OTUD6A and CDC6 in the indicated H1299 cells were determined by Western blotting. **t, u**, CCK8 assays (**t**) and colony formation assays (**u**) were used to examine the proliferation of the indicated H1299 cells. **v**, The protein levels of OTUD6A and CDC6 in the indicated KYSE150 cells were determined by Western blotting. **w, x**, CCK8 assays (**w**) and colony formation assays (**x**) were used to examine the proliferation of the indicated KYSE150 cells. All quantitative analyses were based on three independent experiments. The error bars indicate the SDs. **P* < 0.05, ***P* < 0.01, ****P* < 0.001, n.s. not significant, based on two-tailed Student’s *t* test.


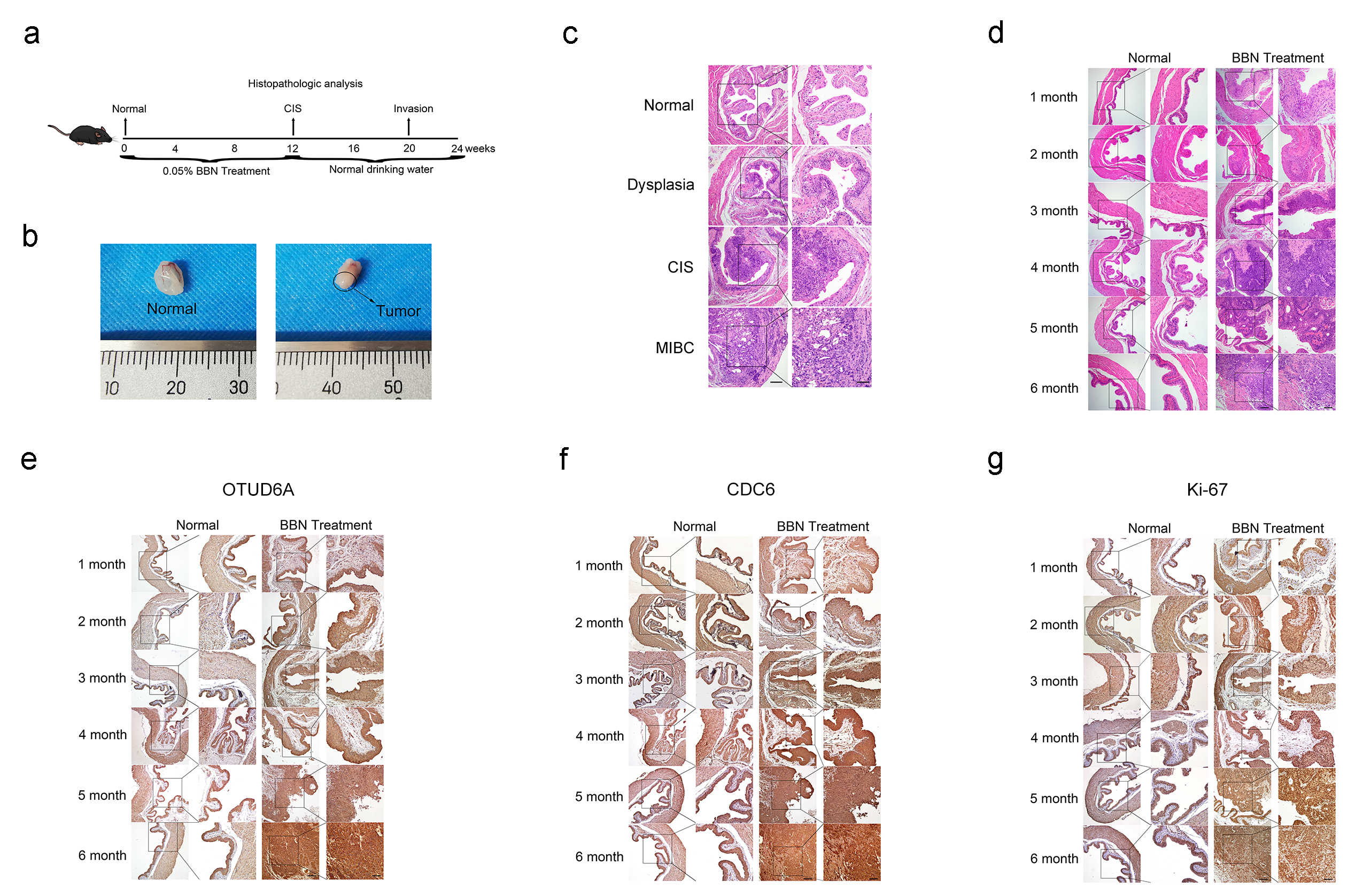


**Supplementary Fig. 9 Histopathology of BBN-induced BCa.** **a**, Diagram of the BBN treatment strategy. **b**, Representative images of normal mouse bladders and mouse bladders after 20 weeks of BBN treatment are shown. **c**, Representative images of normal bladder, dysplasia, carcinoma *in situ* (CIS), muscle invasive bladder cancers are indicated. **d-g**, Histological and protein expression changes were observed at different time points during BBN treatment. Representative images of H&E staining (**d**) and IHC staining of OTUD6A (**e**), CDC6 (**f**) and Ki-67 (**g**) are shown. Scale bars, 100 μm (left) and 50 μm (right).


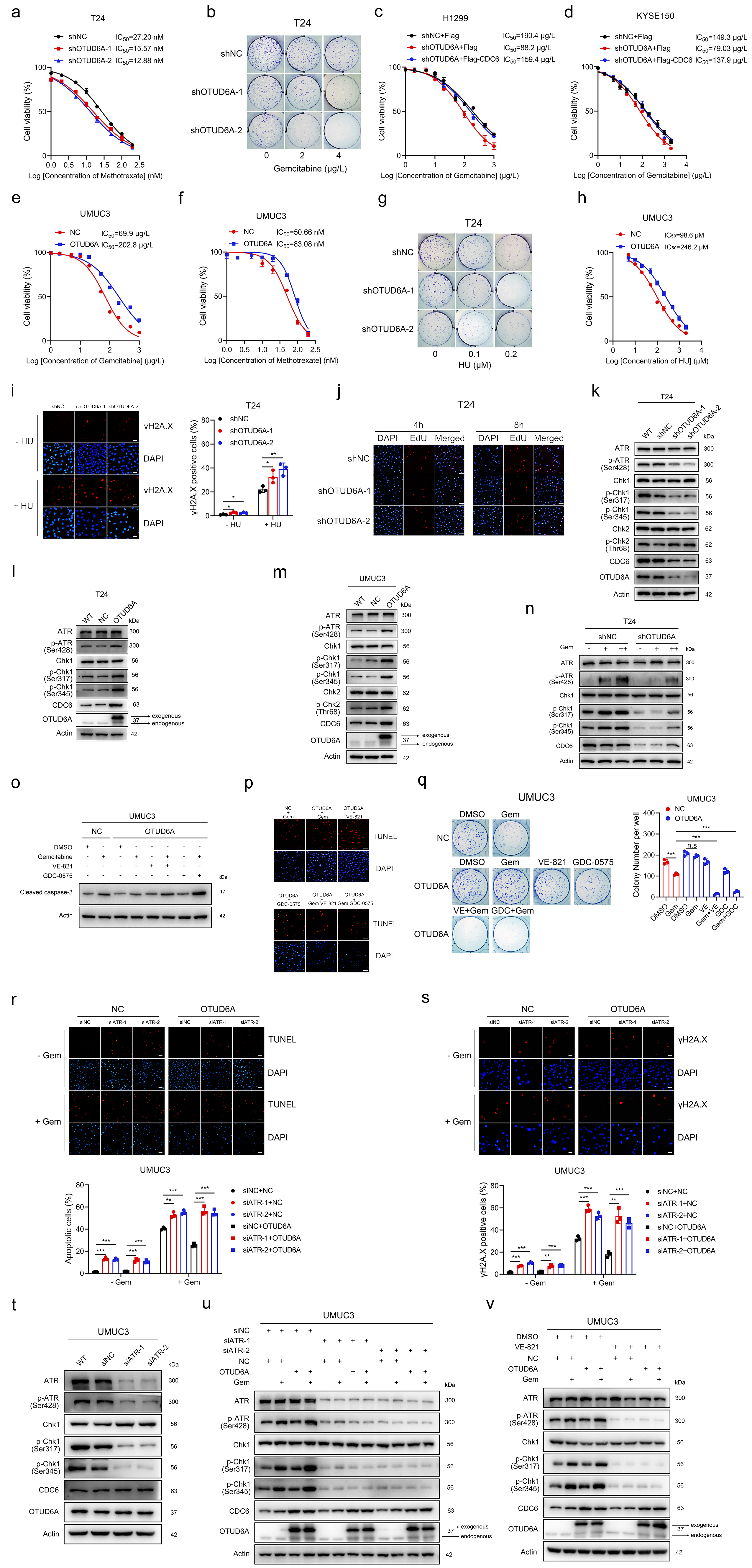


**Supplementary Fig. 10 OTUD6A regulates chemosensitivity via the ATR-Chk1 pathway.** **a**, The cell viability of the indicated T24 cells was determined after 48 h of continuous exposure to multiple concentrations of methotrexate. The IC_50_ value was defined as the concentration causing a 50% decrease in cell viability. The IC_50_ values were estimated by nonlinear regression using a variable Hill slope model. **b**, Representative images of colony formation assays of the indicated T24 cells treated with different concentrations of gemcitabine are shown. **c, d**, The cell viability of the indicated H1299 (**c**) and KYSE150 (**d**) cells was determined after 48 h of continuous exposure to multiple concentrations of gemcitabine. **e**, **f**, The cell viability of the indicated UMUC3 cells was determined after 48 h of continuous exposure to multiple concentrations of gemcitabine (**e**) and methotrexate (**f**). **g**, Representative images of colony formation assays of the indicated T24 cells treated with different concentrations of hydroxyurea (HU) are shown. **h**, The cell viability of the indicated UMUC3 cells was determined after 48 h of continuous exposure to multiple concentrations of HU. **i**, The γH2A.X protein level was measured by immunofluorescence staining in the indicated T24 cells treated with or without 200 μM HU for 48 h. Scale bars, 20 μm. **j**, Representative images of EdU staining are shown. Scale bars, 50 μm. **k**, The effect of OTUD6A knockdown on the ATR-Chk1 signalling pathway in T24 cells was determined by Western blotting. **l, m**, The effect of OTUD6A overexpression on the ATR-Chk1 signalling pathway in T24 (**l**) and UMUC3 (**m**) cells was determined by Western blotting. **n**, ATR-Chk1 pathway protein levels in OTUD6A-knockdown T24 cells treated with multiple concentrations of gemcitabine for 6 h were determined by Western blotting. **o**, The effect of ATR and Chk1 inhibitors on regulating the sensitivity of OTUD6A-overexpressing UMUC3 cells to gemcitabine was determined by Western blotting. **p**, Representative images of TUNEL assays of the indicated UMUC3 cells treated with or without gemcitabine, ATR inhibitors and Chk1 inhibitors are shown. Scale bars, 50 μm. **q**, The effects of ATR and Chk1 inhibitors (VE-821 and GDC-0575) on regulating the sensitivity of OTUD6A-overexpressing UMUC3 cells to gemcitabine was determined by colony formation assays. **r**, Apoptosis was measured by TUNEL assays in the indicated UMUC3 cells treated with 100 μg/L gemcitabine for 48 h. Representative images are shown (top). Scale bars, 50 μm. **s**, The γH2A.X protein level was measured by immunofluorescence staining in the indicated UMUC3 cells treated with or without 100 μg/L gemcitabine for 48 h. Representative immunofluorescence images are shown (top). Scale bars, 20 μm. **t**, The effect of ATR knockown on the ATR-Chk1 signalling pathway in UMUC3 cells was determined by Western blotting. **u**, ATR-Chk1 pathway protein levels in the indicated UMUC3 cells treated with 200 μg/L gemcitabine for 6 h were determined by Western blotting. **v**, The effect of ATR inhibitor on regulating the sensitivity of OTUD6A-overexpressing UMUC3 cells to gemcitabine was determined by Western blotting. All quantitative analyses were based on three independent experiments. The error bars indicate the SDs. **P* < 0.05, ***P* < 0.01, ****P* < 0.001, n.s. not significant, based on two-tailed Student’s *t* test.


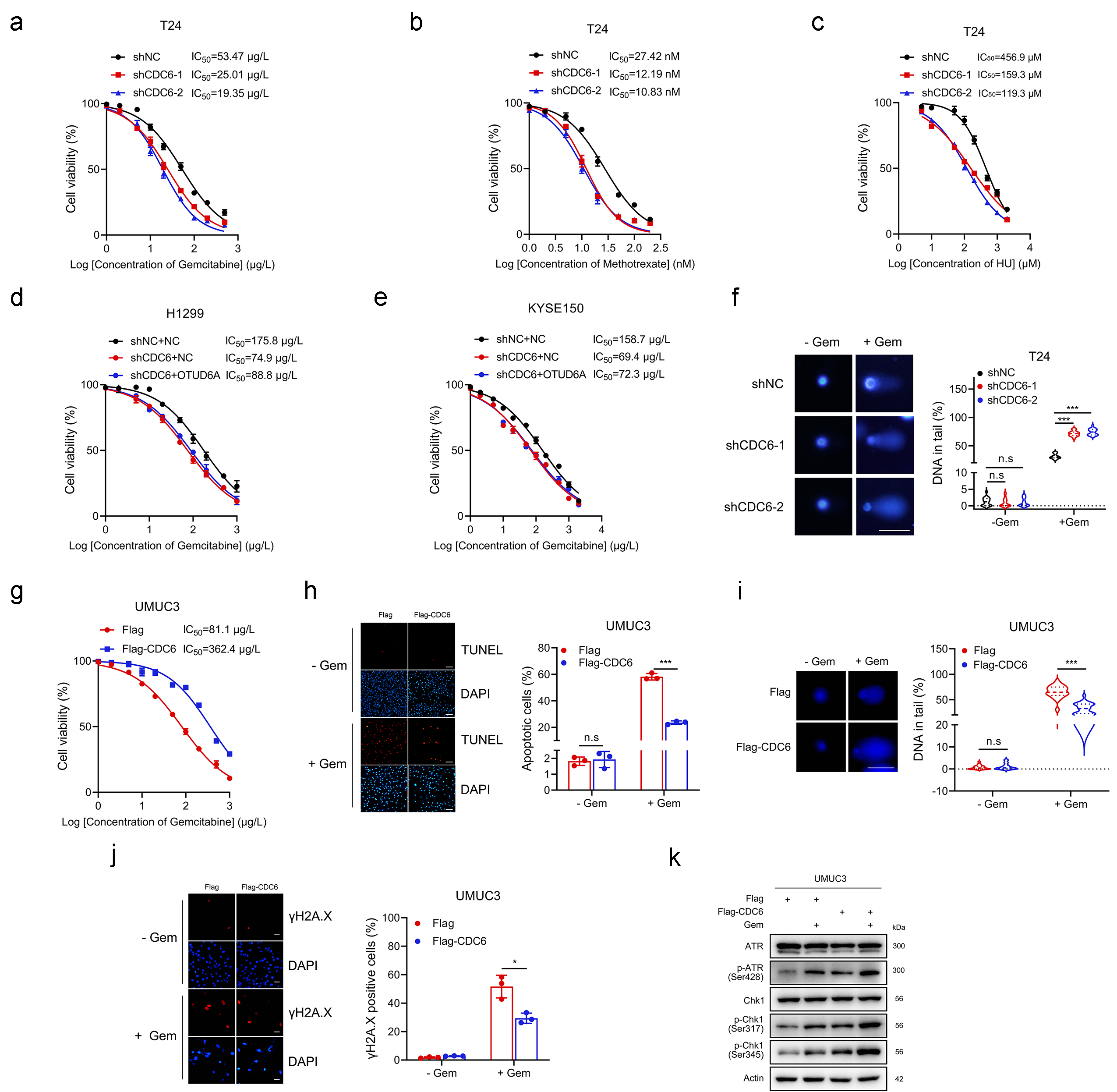


**Supplementary Fig. 11 CDC6 decreases sensitivity to chemotherapy via the ATR-Chk1 pathway.** **a-c**, The cell viability of the indicated T24 cells was determined after 48 h of continuous exposure to multiple concentrations of gemcitabine (**a**), methotrexate (**b**) and HU (**c**). **d, e**, The cell viability of the indicated H1299 (**d**) and KYSE150 (**e**) cells was determined after 48 h of continuous exposure to multiple concentrations of gemcitabine. **f**, The amount of DNA strand breaks was quantified by alkaline comet assays in the indicated T24 cells treated with or without 20 μg/L gemcitabine for 48 h. Scale bars, 20 μm. **g**, The cell viability of the indicated UMUC3 cells was determined after 48 h of continuous exposure to multiple concentrations of gemcitabine. **h**, Apoptosis was measured by TUNEL assays in the indicated UMUC3 cells treated with or without 100 μg/L gemcitabine for 48 h. Representative images are shown (left). Scale bars, 50 μm. **i**, The amount of DNA strand breaks was quantified by alkaline comet assays in the indicated UMUC3 cells treated with or without 100 μg/L gemcitabine for 48 h. Representative images are shown (left). Scale bars, 20 μm. **j**, The γH2A.X protein level was measured by immunofluorescence staining in the indicated UMUC3 cells treated with or without 100 μg/L gemcitabine for 48 h. Representative immunofluorescence images are shown (left). Scale bars, 20 μm. **k**, ATR-Chk1 pathway protein levels in the indicated UMUC3 cells treated with 200 μg/L gemcitabine for 6 h were determined by Western blotting. All quantitative analyses were based on three independent experiments. The error bars indicate the SDs. **P* < 0.05, ****P* < 0.001, n.s. not significant, based on two-tailed Student’s *t* test.


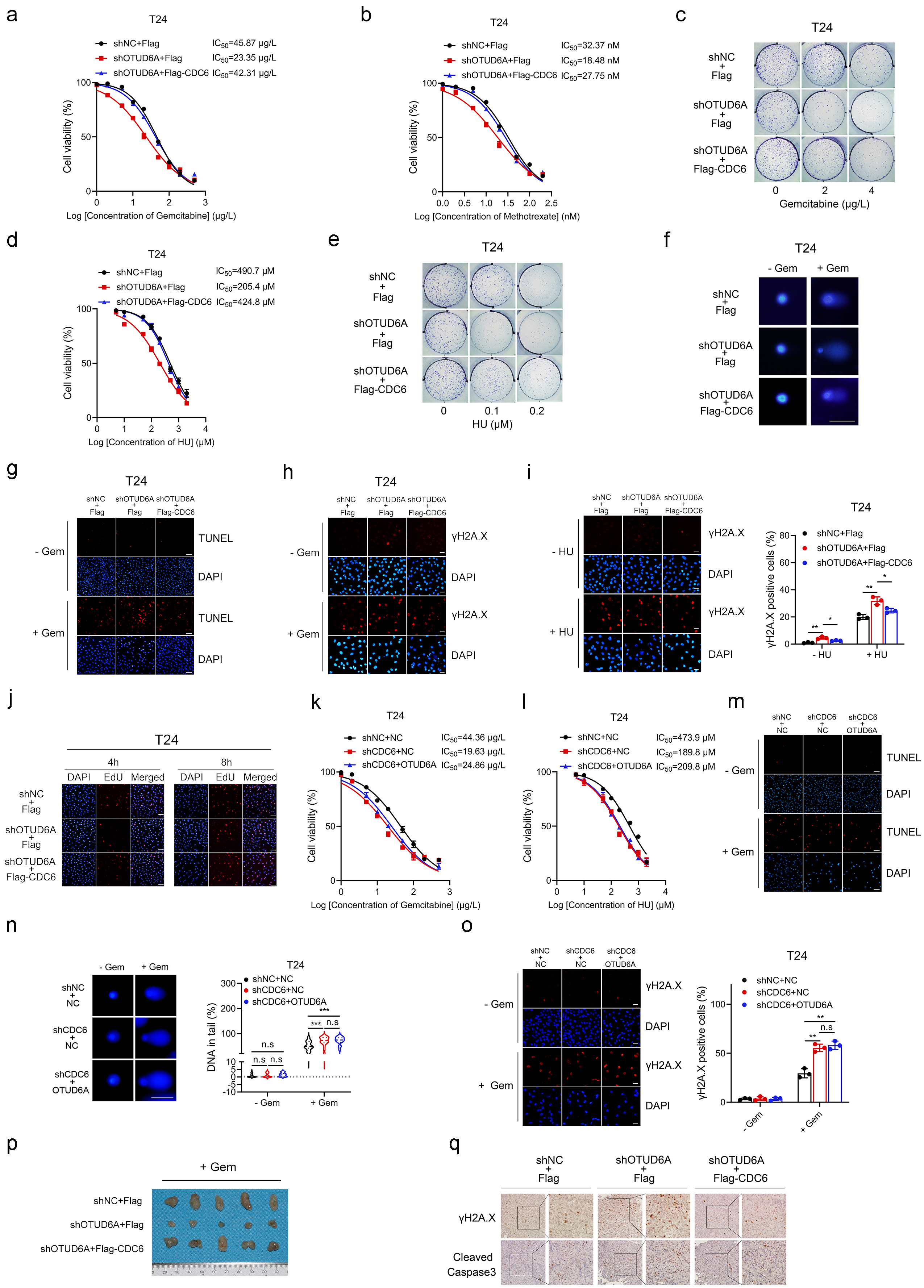


**Supplementary Fig. 12 OTUD6A decreases sensitivity to chemotherapy via the CDC6-ATR-Chk1 pathway. a**, **b**, The cell viability of the indicated T24 cells was determined after 48 h of continuous exposure to multiple concentrations of gemcitabine (**a**) and methotrexate (**b**). **c**, Representative images of colony formation assays of the indicated T24 cells treated with different concentrations of gemcitabine are shown. **d**, The cell viability of the indicated T24 cells was determined after 48 h of continuous exposure to multiple concentrations of HU. **e,** Representative images of colony formation assays of the indicated T24 cells treated with different concentrations of HU are shown. **f-h**, Representative images of alkaline comet assays (**f**), TUNEL assays (**g**) and immunofluorescence staining of γH2A.X (**h**) of the indicated T24 cells treated with or without gemcitabine are shown. Scale bars, 50 μm (**g**), 20 μm (**f, h**). **i**, The γH2A.X protein level was measured by immunofluorescence staining in the indicated T24 cells treated with or without 200 μM HU for 48 h. Representative immunofluorescence images are shown (left). Scale bars, 20 μm. **j**, Representative images of EdU staining are shown. Scale bars, 50 μm. **k, l**, The cell viability of the indicated T24 cells was determined after 48 h of continuous exposure to multiple concentrations of gemcitabine (**k**) and HU (**l**). **m**, Representative images of TUNEL assays of the indicated T24 cells treated with or without gemcitabine are shown. Scale bars, 50 μm. **n**, The amount of DNA strand breaks was quantified by alkaline comet assays in the indicated T24 cells treated with or without 20 μg/L gemcitabine for 48 h. Representative images are shown (left). Scale bars, 20 μm. **o**, The γH2A.X protein level was measured by immunofluorescence staining in the indicated T24 cells treated with or without 20 μg/L gemcitabine for 48 h. Representative immunofluorescence images are shown (left). Scale bars, 20 μm. **p**, Images of subcutaneous tumours formed by the indicated T24 cells treated with gemcitabine are shown. **q**, Representative IHC images showing γH2A.X and cleaved caspase-3 proteins in the indicated subcutaneous T24 tumours are shown. Scale bars, 50 μm (left) and 20 μm (right). All quantitative analyses were based on three independent experiments. The error bars indicate the SDs. **P* < 0.05, ***P* < 0.01, ****P* < 0.001, n.s. not significant, based on two-tailed Student’s *t* test.


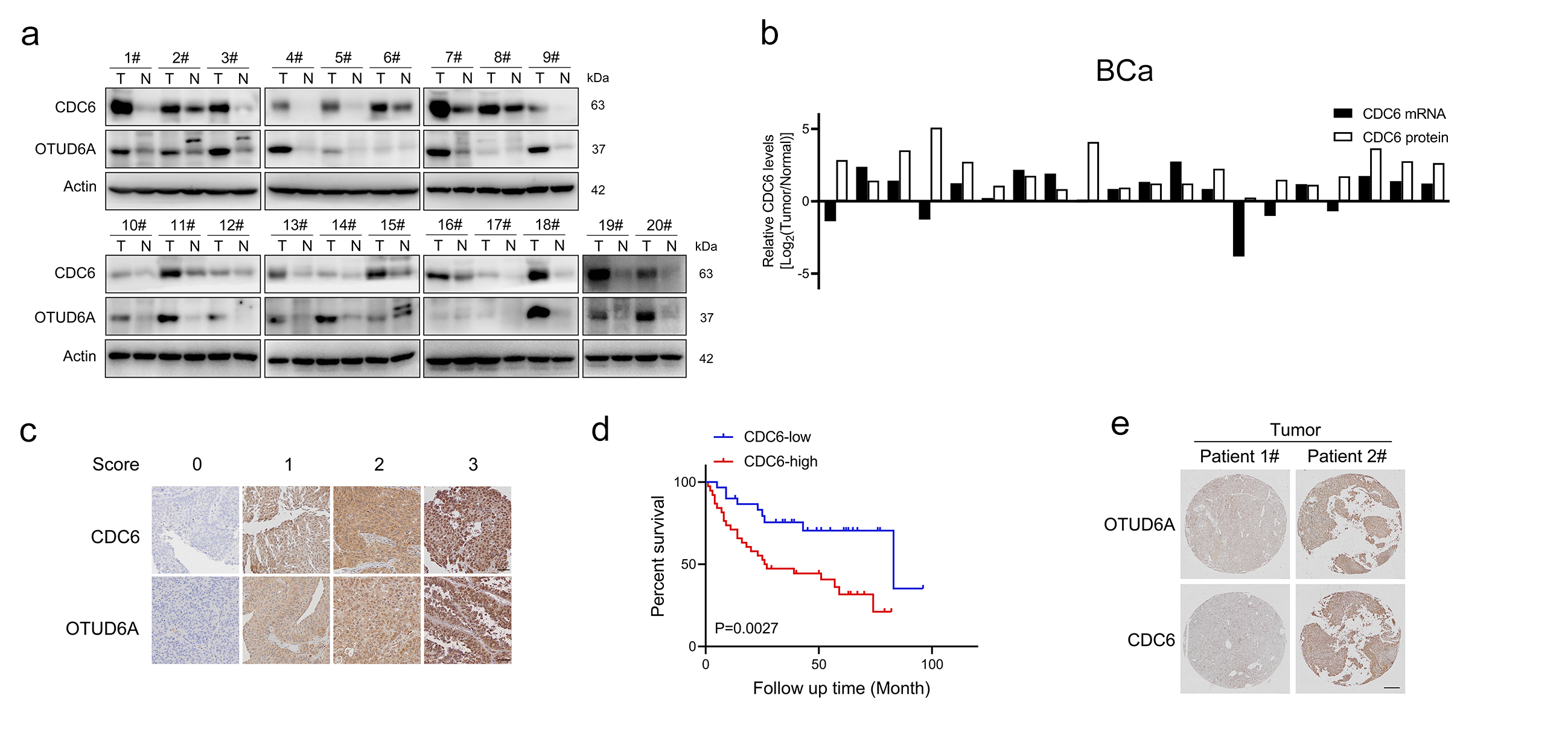


**Supplementary Fig. 13 CDC6 protein expression is correlated with the OTUD6A protein level in tumour tissues.** **a**, The protein expression levels of OTUD6A and CDC6 in 20 human BCa tissues and matched adjacent normal bladder tissues were determined by Western blotting. T, BCa tissue; N, Normal bladder tissue. **b**, Relative CDC6 protein and mRNA levels in BCa tissues are shown. **c**, The representative images of score from 0 to 3 for IHC staining intensity are shown. Scale bars, 50 μm. **d**, Kaplan-Meier curves of overall survival for patients with BCa stratified by the CDC6 expression level in the tissue microarray are shown. Data were analysed using the log-rank test. **e**, Representative IHC images showing OTUD6A and CDC6 expression in the renal carcinoma tissue microarray are shown. Scale bar, 500 μm.


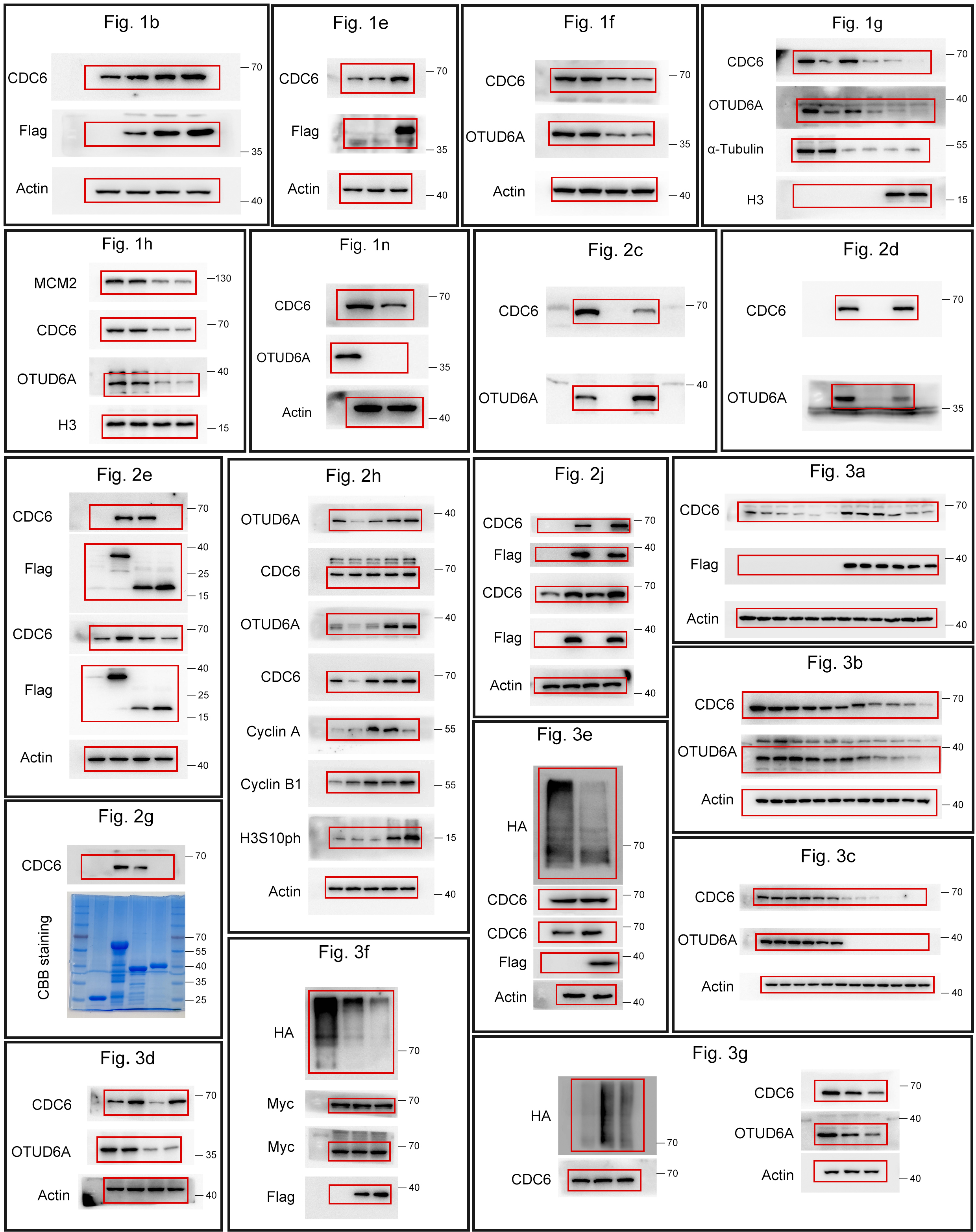


**Supplementary Fig. 14** The original WB figures in Fig. 1-3.


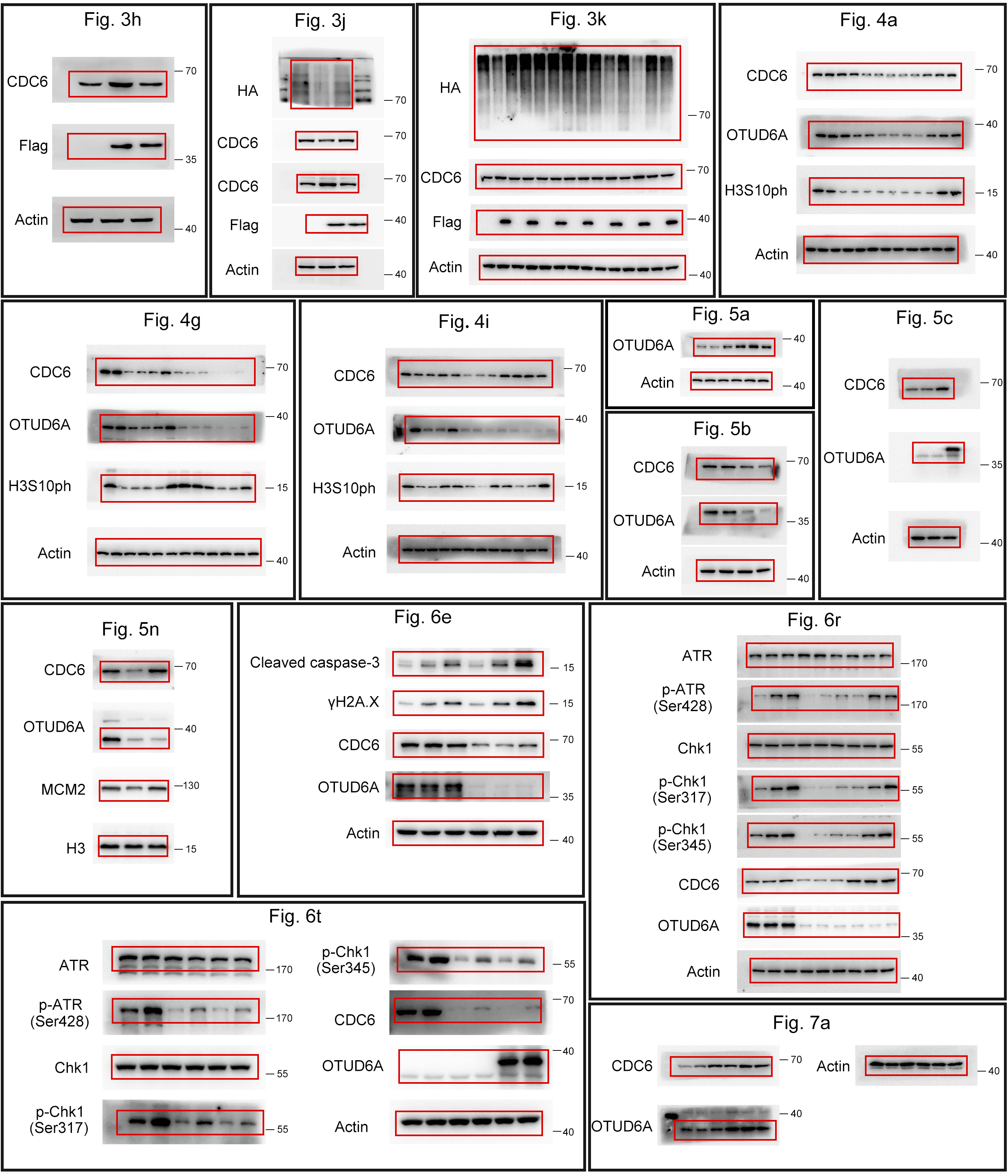


**Supplementary Fig. 15** The original WB figures in Fig. 3-7.


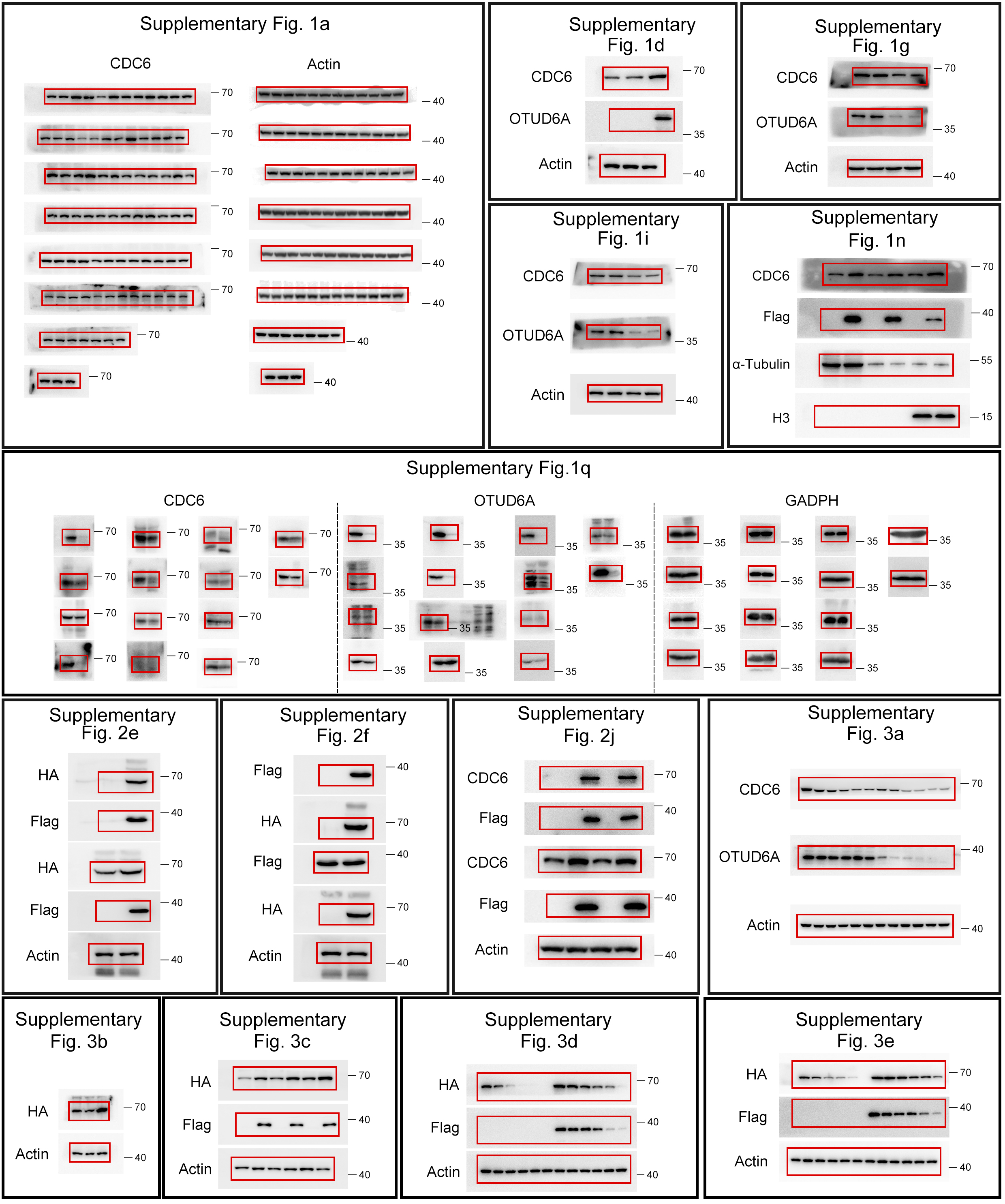


**Supplementary Fig. 16** The original WB figures in Supplementary Fig. 1-3.


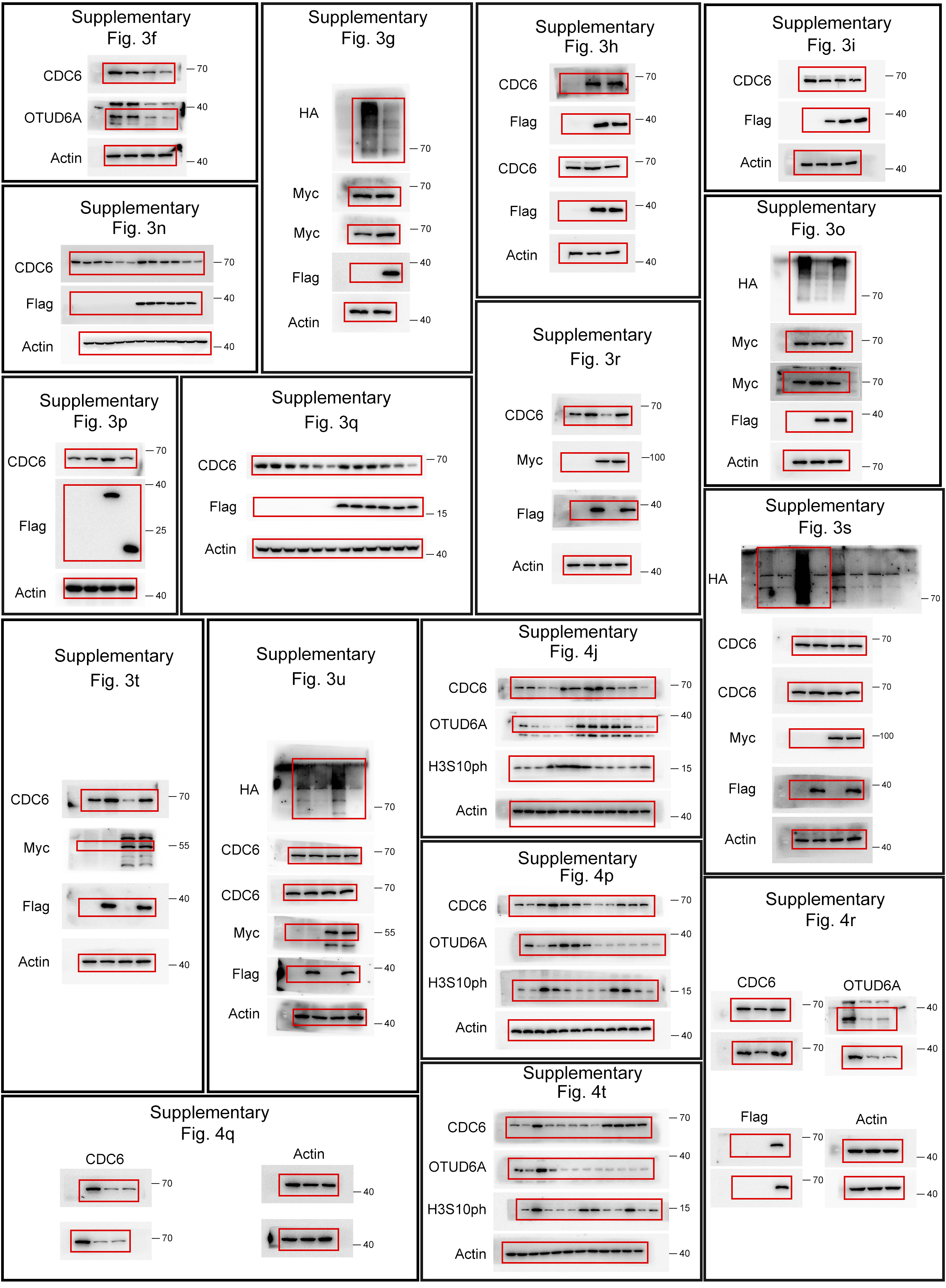


**Supplementary Fig. 17** The original WB figures in Supplementary Fig. 3, 4.


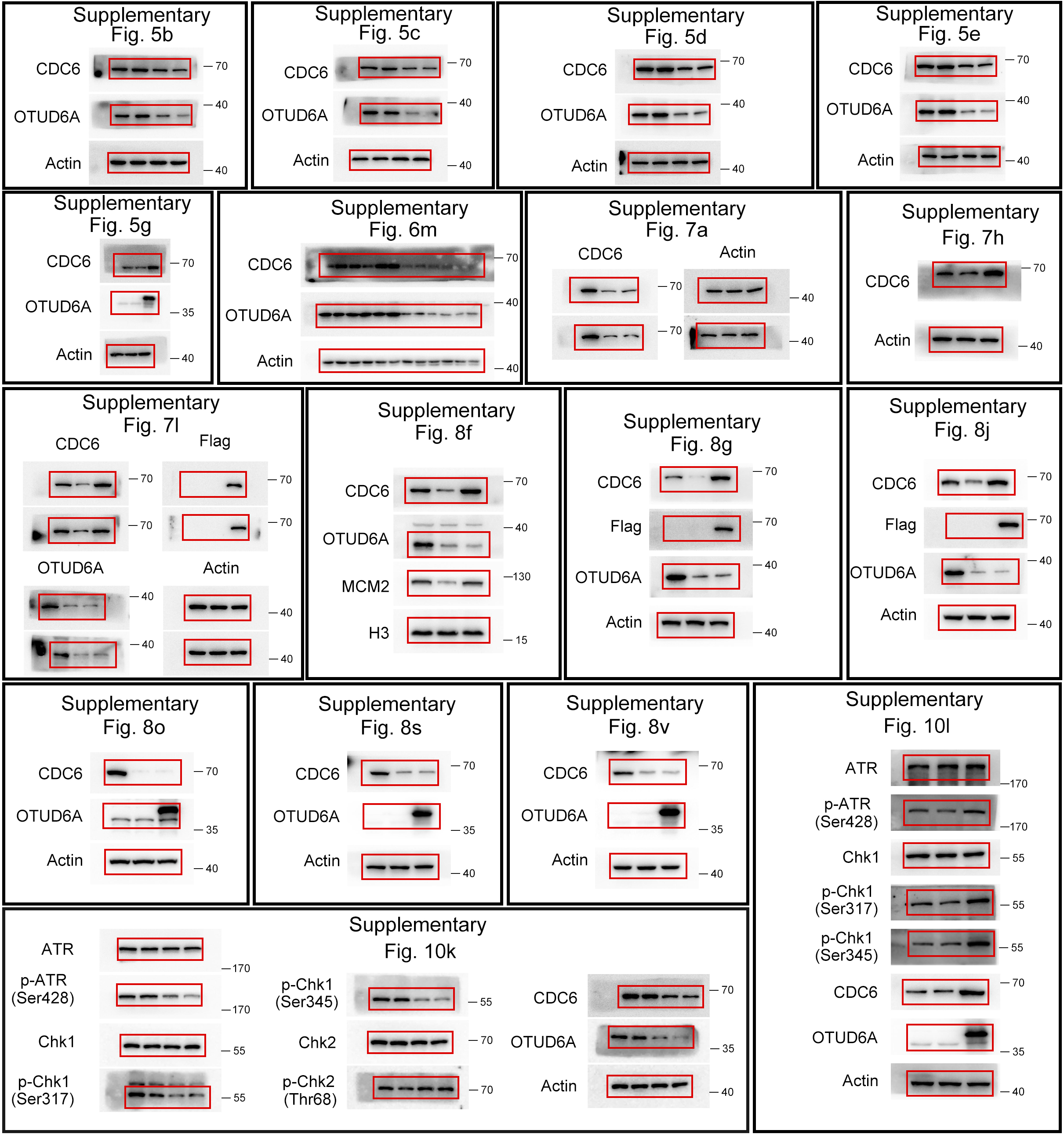


**Supplementary Fig. 18** The original WB figures in Supplementary Fig. 5-10.


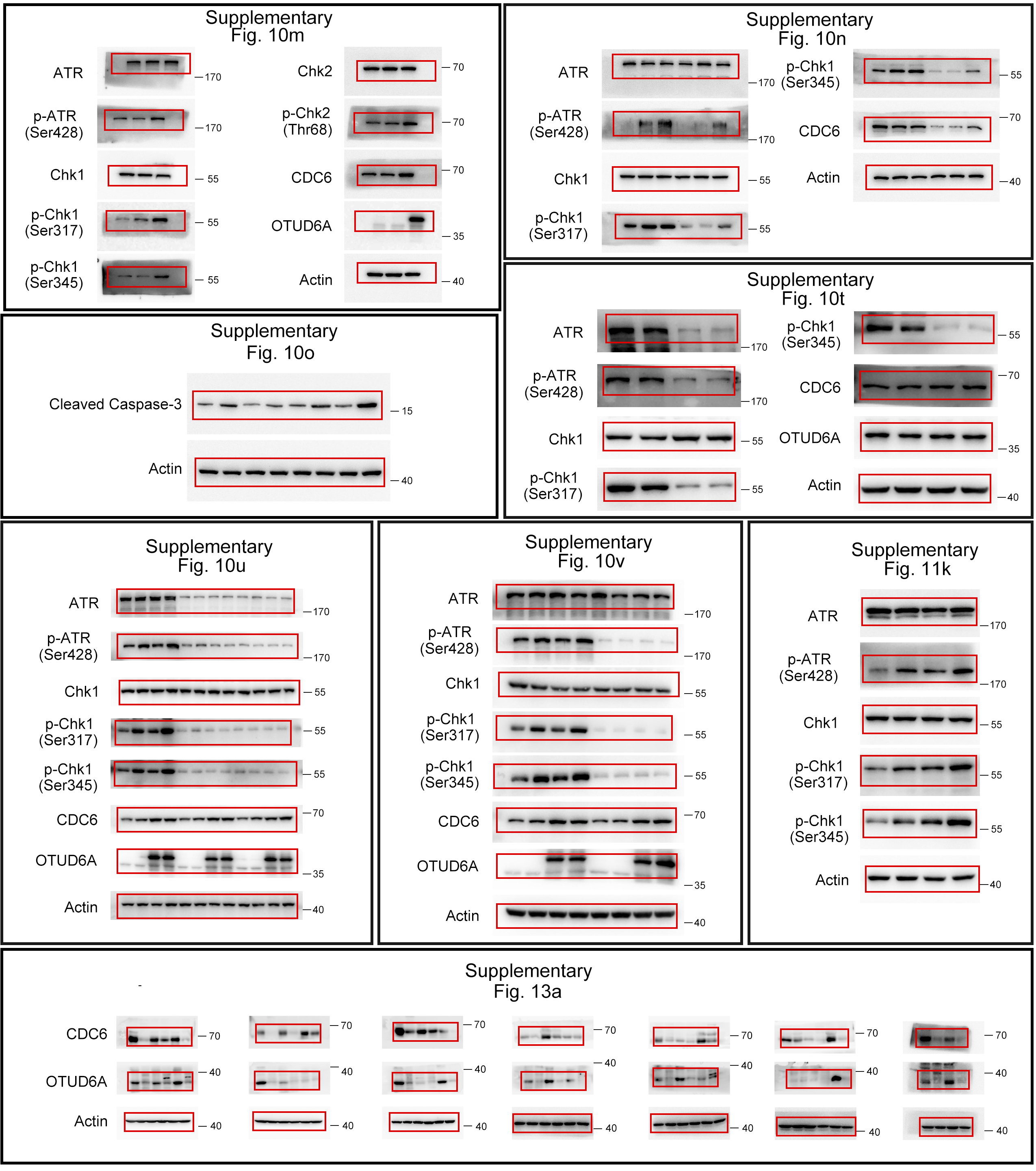


**Supplementary Fig. 19** The original WB figures in Supplementary Fig. 10-13.

| Supplementary Table 1 Primer sequences used for construction of mutant vectors | | |
| --- | --- | --- |
| Mutated sites | Primers | Sequences |
| OTUD6A^C152A^ | Forward | TCCCGGCCGACGGCCACGCCATGTACCGCGCCATCCAAGA |
| OTUD6A^C152A^ | Reverse | TCTTGGATGGCGCGGTACATGGCGTGGCCGTCGGCCGGGA |

| Supplementary Table 2 qRT-PCR primer sequences | | | |
| --- | --- | --- | --- |
| Gene name | Primer | Sequences | Product length（bp） |
| CDC6 | Forward | ACCTATGCAACACTCCCCATT | 153 |
|  | Reverse | TGGCTAGTTCTCTTTTGCTAGGA |  |
| OTUD6A | Forward | GAAGTTCCAAGACGACAGTAGC | 172 |
|  | Reverse | CAGGTGCTCCGACATCTCA |  |
| GAPDH | Forward | GGAGCGAGATCCCTCCAAAAT | 197 |
|  | Reverse | GGCTGTTGTCATACTTCTCATGG |  |

| Supplementary Table 3 Antibodies used in Western blotting, immunofluorescence, immunohistochemistry, chromatin immunoprecipitation and immunoprecipitation | | | |
| --- | --- | --- | --- |
| Antibody | Company | Catalog number | Application |
| anti-Flag-Tag | Sigma Aldrich | F1804 | WB, IP, IF |
| anti-CDC6 | Santa Cruz | sc-9964 | WB |
| anti-CDC6 | Abcam | ab109315 | IF, IHC |
| anti-CDC6 | Abcam | ab188423 | IP |
| anti-CDC6 | ProteinTech | 66021-1-Ig | IF |
| anti-OTUD6A | ProteinTech | 24486-1-AP | WB, IF |
| anti-OTUD6A | Invitrogen | PA5-110066 | WB, IHC |
| anti-MCM2 | BD Biosciences | 610701 | WB |
| anti-γ-tubulin | Santa Cruz | sc-17787 | IF |
| anti-HA-Tag | Abcam | ab18181 | WB, IP |
| anti-Myc-Tag | CST | 2276 | WB, IP |
| anti-β-actin | Sigma Aldrich | A5441 | WB |
| anti-Cyclin A | Invitrogen | MA1-154 | WB |
| anti-Cyclin B1 | Abcam | ab32053 | WB |
| anti-phospho-Histone H3 (Ser10) | ProteinTech | 66863-1-Ig | WB |
| anti-GAPDH | Abways | AB0037 | WB |
| anti-c-myc | CST | 9402 | WB |
| anti-α-Tubulin | Santa Cruz | sc-32293 | WB, IF |
| anti-Histone H3 | Abways | CY6587 | WB |
| anti-ATR | CST | 13934 | WB |
| anti-p-ATR (Ser428) | CST | 2853 | WB |
| anti-Chk1 | CST | 2360 | WB |
| anti-p-Chk1 (Ser317) | CST | 12302 | WB |
| anti-p-Chk1 (Ser345) | CST | 2348 | WB |
| anti-Chk2 | CST | 6334 | WB |
| anti-p-Chk2 (Thr68) | CST | 2197 | WB |
| anti-γ-H2A.X | CST | 9718 | WB, IF, IHC |
| anti-cleaved caspase-3 | Affinity | AF7022 | WB |
| anti-Ki-67 | Invitrogen | PA5-19462 | IHC, IF |
| GAPDH | ProteinTech | 60004-1-Ig | WB |

| Supplementary Table 4 Primer sequences used for identifying mice genotypes | | | |
| --- | --- | --- | --- |
| Application | Primer | Sequences | Product length（bp） |
| Otud6a without Cre activity | Forward | TAGGCTGAAAATGGAAATTGTGGG | Flox: 203 WT: 123 |
|  | Reverse | TAAGTCATTTCAGCCACACCAACT |  |
| Otud6a with Cre activity | Forward | GGGAATGGAGCAGTATCTAAAGGC | 187 |
|  | Reverse | TAAGTCATTTCAGCCACACCAACT |  |

| Supplementary Table 5 Proteomic analysis of 84 differentially expressed proteins | | | | | | | | | |
| --- | --- | --- | --- | --- | --- | --- | --- | --- | --- |
| Protein accession | Gene name | OTUD6A/Flag Ratio | Regulated Type | MW [kDa] | Coverage [%] | Peptides | Unique peptides | Flag | OTUD6A |
| O00257 | CBX4 | 1.507 | Up | 61.367 | 10.4 | 4 | 4 | 0.797913 | 1.202087 |
| O00401 | WASL | 0.522 | Down | 54.826 | 12.1 | 3 | 3 | 1.314437 | 0.685563 |
| O00488 | ZNF593 | 1.547 | Up | 15.199 | 45.5 | 4 | 4 | 0.785289 | 1.214711 |
| O60831 | PRAF2 | 0.312 | Down | 19.258 | 22.5 | 3 | 3 | 1.524578 | 0.475422 |
| O75385 | ULK1 | 1.601 | Up | 112.63 | 1.9 | 2 | 2 | 0.768824 | 1.231176 |
| O95905 | ECD | 1.502 | Up | 72.757 | 11.5 | 5 | 5 | 0.79938 | 1.20062 |
| P11274 | BCR | 1.557 | Up | 142.82 | 2.8 | 3 | 2 | 0.782137 | 1.217863 |
| P12236 | SLC25A6 | 0.652 | Down | 32.866 | 51.3 | 13 | 2 | 1.210928 | 0.789072 |
| P22492 | H1-6 | 0.412 | Down | 22.019 | 15.9 | 3 | 2 | 1.416283 | 0.583717 |
| P27448 | MARK3 | 3.132 | Up | 84.428 | 14.9 | 7 | 5 | 0.484028 | 1.515972 |
| P31949 | S100A11 | 0.662 | Down | 11.74 | 34.3 | 3 | 3 | 1.203152 | 0.796848 |
| P39060 | COL18A1 | 0.601 | Down | 178.19 | 3.2 | 4 | 4 | 1.248833 | 0.751167 |
| P43121 | MCAM | 1.78 | Up | 71.607 | 5.7 | 3 | 3 | 0.719549 | 1.280451 |
| P46013 | MKI67 | 1.625 | Up | 358.69 | 14.6 | 28 | 28 | 0.761886 | 1.238114 |
| P49759 | CLK1 | 1.523 | Up | 57.29 | 11 | 4 | 3 | 0.792732 | 1.207268 |
| P49760 | CLK2 | 0.306 | Down | 60.089 | 8 | 3 | 2 | 1.53145 | 0.46855 |
| P54725 | RAD23A | 0.663 | Down | 39.609 | 30.6 | 8 | 5 | 1.202543 | 0.797457 |
| P57105 | SYNJ2BP | 0.462 | Down | 15.928 | 24.8 | 3 | 3 | 1.368189 | 0.631811 |
| Q13315 | ATM | 0.642 | Down | 350.68 | 1.4 | 3 | 3 | 1.218302 | 0.781698 |
| Q13641 | TPBG | 0.644 | Down | 46.031 | 6.7 | 2 | 2 | 1.216179 | 0.783821 |
| Q14653 | IRF3 | 1.615 | Up | 47.219 | 17.1 | 4 | 4 | 0.764901 | 1.235099 |
| Q16854 | DGUOK | 0.572 | Down | 32.055 | 16.6 | 2 | 2 | 1.27234 | 0.72766 |
| Q1L5Z9 | LONRF2 | 1.702 | Up | 83.653 | 9 | 4 | 4 | 0.740064 | 1.259936 |
| Q32NB8 | PGS1 | 1.526 | Up | 62.73 | 4.5 | 2 | 2 | 0.791817 | 1.208183 |
| Q3ZAQ7 | VMA21 | 4.831 | Up | 11.354 | 24.8 | 2 | 2 | 0.343007 | 1.656993 |
| Q460N5 | PARP14 | 0.141 | Down | 202.8 | 4.1 | 5 | 5 | 1.752982 | 0.247018 |
| Q53RE8 | ANKRD39 | 2.189 | Up | 19.651 | 15.3 | 2 | 2 | 0.62714 | 1.37286 |
| Q5XKP0 | MICOS13 | 0.623 | Down | 13.087 | 35.6 | 2 | 2 | 1.231913 | 0.768087 |
| Q6UVK1 | CSPG4 | 0.638 | Down | 250.53 | 1.7 | 3 | 3 | 1.220767 | 0.779233 |
| Q712K3 | UBE2R2 | 1.607 | Up | 27.166 | 10.1 | 2 | 2 | 0.7672 | 1.2328 |
| Q71F23 | CENPU | 1.761 | Up | 47.521 | 17.2 | 5 | 5 | 0.724285 | 1.275715 |
| Q7Z333 | SETX | 0.326 | Down | 302.88 | 0.7 | 2 | 2 | 1.507811 | 0.492189 |
| Q86T24 | ZBTB33 | 0.222 | Down | 74.484 | 5.1 | 2 | 2 | 1.636778 | 0.363222 |
| Q86UT6 | NLRX1 | 0.312 | Down | 107.61 | 2.8 | 2 | 2 | 1.52449 | 0.47551 |
| Q86WV6 | STING1 | 0.53 | Down | 42.192 | 18.7 | 4 | 4 | 1.307197 | 0.692803 |
| Q8IYU8 | MICU2 | 0.664 | Down | 49.666 | 18.9 | 6 | 6 | 1.202021 | 0.797979 |
| Q8N9B5 | JMY | 1.553 | Up | 111.44 | 3.8 | 3 | 3 | 0.783298 | 1.216702 |
| Q8N9N5 | BANP | 0.573 | Down | 56.494 | 4.4 | 2 | 2 | 1.271713 | 0.728287 |
| Q8NFA0 | USP32 | 1.536 | Up | 181.65 | 2.1 | 3 | 3 | 0.788791 | 1.211209 |
| Q8TEY7 | USP33 | 1.566 | Up | 106.73 | 2.2 | 2 | 2 | 0.779562 | 1.220438 |
| Q8WUQ7 | CACTIN | 0.559 | Down | 88.701 | 4.9 | 2 | 2 | 1.283041 | 0.716959 |
| Q8WV22 | NSMCE1 | 0.645 | Down | 30.855 | 15.4 | 3 | 3 | 1.21553 | 0.78447 |
| Q92558 | WASF1 | 0.157 | Down | 61.651 | 7.2 | 3 | 3 | 1.729267 | 0.270733 |
| Q92575 | UBXN4 | 0.624 | Down | 56.777 | 14 | 4 | 4 | 1.231253 | 0.768747 |
| Q92993 | KAT5 | 2.976 | Up | 58.581 | 10.5 | 4 | 4 | 0.503031 | 1.496969 |
| Q969F9 | HPS3 | 0.666 | Down | 113.73 | 8 | 5 | 5 | 1.200587 | 0.799413 |
| Q96CS2 | HAUS1 | 1.758 | Up | 31.863 | 10.1 | 2 | 2 | 0.725232 | 1.274768 |
| Q96E29 | MTERF3 | 0.65 | Down | 47.971 | 6.2 | 2 | 2 | 1.212291 | 0.787709 |
| Q96EA4 | SPDL1 | 2.69 | Up | 70.171 | 9.1 | 4 | 4 | 0.54201 | 1.45799 |
| Q96EC8 | YIPF6 | 5.361 | Up | 26.256 | 8.5 | 2 | 2 | 0.314434 | 1.685566 |
| Q96GE9 | DMAC1 | 0.659 | Down | 12.257 | 32.8 | 2 | 2 | 1.205457 | 0.794543 |
| Q96N21 | TEPSIN | 0.532 | Down | 55.136 | 5.7 | 2 | 2 | 1.30538 | 0.69462 |
| Q96QE3 | ATAD5 | 1.548 | Up | 207.57 | 1.5 | 2 | 2 | 0.785023 | 1.214977 |
| Q96QE5 | TEFM | 0.572 | Down | 41.676 | 5.8 | 2 | 2 | 1.272051 | 0.727949 |
| Q96QR8 | PURB | 1.575 | Up | 33.24 | 10.3 | 3 | 3 | 0.776795 | 1.223205 |
| Q96S94 | CCNL2 | 1.526 | Up | 58.147 | 9.4 | 3 | 3 | 0.791635 | 1.208365 |
| Q99538 | LGMN | 8.464 | Up | 49.411 | 7.2 | 2 | 2 | 0.211321 | 1.788679 |
| Q99741 | CDC6 | 1.611 | Up | 62.72 | 7.3 | 3 | 3 | 0.765943 | 1.234057 |
| Q9BTT4 | MED10 | 1.566 | Up | 15.688 | 22.2 | 2 | 2 | 0.779547 | 1.220453 |
| Q9BW62 | KATNAL1 | 1.535 | Up | 55.392 | 9.4 | 3 | 2 | 0.789038 | 1.210962 |
| Q9BXI6 | TBC1D10A | 1.673 | Up | 57.117 | 7.7 | 4 | 4 | 0.748286 | 1.251714 |
| Q9BYE7 | PCGF6 | 2.199 | Up | 39.047 | 8.6 | 2 | 2 | 0.625131 | 1.374869 |
| Q9C0H2 | TTYH3 | 0.658 | Down | 57.544 | 8.6 | 3 | 3 | 1.206472 | 0.793528 |
| Q9GZN1 | ACTR6 | 1.619 | Up | 45.81 | 6.3 | 2 | 2 | 0.763628 | 1.236372 |
| Q9H082 | RAB33B | 0.643 | Down | 25.717 | 13.1 | 2 | 2 | 1.217582 | 0.782418 |
| Q9H2D6 | TRIOBP | 0.475 | Down | 261.37 | 1 | 2 | 2 | 1.356213 | 0.643787 |
| Q9H7B4 | SMYD3 | 0.228 | Down | 49.097 | 10.3 | 3 | 3 | 1.628002 | 0.371998 |
| Q9H9F9 | ACTR5 | 0.508 | Down | 68.297 | 14.5 | 5 | 5 | 1.326047 | 0.673953 |
| Q9HAW4 | CLSPN | 1.544 | Up | 151.09 | 5.5 | 5 | 5 | 0.786286 | 1.213714 |
| Q9NNX1 | TUFT1 | 1.608 | Up | 44.263 | 13.1 | 3 | 3 | 0.76688 | 1.23312 |
| Q9NP50 | SINHCAF | 3.4 | Up | 24.852 | 11.3 | 2 | 2 | 0.45452 | 1.54548 |
| Q9NUJ3 | TCP11L1 | 0.416 | Down | 57.034 | 9.2 | 3 | 3 | 1.412479 | 0.587521 |
| Q9NVC6 | MED17 | 2.079 | Up | 72.889 | 10 | 5 | 5 | 0.64946 | 1.35054 |
| Q9NVN8 | GNL3L | 1.877 | Up | 65.572 | 21 | 9 | 9 | 0.695226 | 1.304774 |
| Q9NVP2 | ASF1B | 0.551 | Down | 22.433 | 15.3 | 2 | 2 | 1.289164 | 0.710836 |
| Q9NW75 | GPATCH2 | 1.774 | Up | 58.943 | 5.7 | 2 | 2 | 0.720973 | 1.279027 |
| Q9NXX6 | NSMCE4A | 0.624 | Down | 44.301 | 19.2 | 5 | 5 | 1.231685 | 0.768315 |
| Q9P086 | MED11 | 0.652 | Down | 13.129 | 31.6 | 2 | 2 | 1.210515 | 0.789485 |
| Q9UIM3 | FKBPL | 0.634 | Down | 38.176 | 9.2 | 3 | 3 | 1.223902 | 0.776098 |
| Q9UJY1 | HSPB8 | 1.556 | Up | 21.604 | 12.8 | 2 | 2 | 0.782517 | 1.217483 |
| Q9ULJ7 | ANKRD50 | 0.661 | Down | 155.86 | 2.3 | 2 | 2 | 1.203841 | 0.796159 |
| Q9Y3B6 | EMC9 | 3.347 | Up | 23.061 | 13.9 | 2 | 2 | 0.46007 | 1.53993 |
| Q9Y3C0 | WASHC3 | 0.587 | Down | 21.172 | 16 | 2 | 2 | 1.259958 | 0.740042 |
| Q9Y3E7 | CHMP3 | 0.583 | Down | 25.073 | 9 | 2 | 2 | 1.263793 | 0.736207 |
